# Supplementary figures and images for: Identification of hub genes and prediction of the ceRNA network in adult sepsis
Source: PeerJ. 2025 Aug 13;13:e19619. doi: 10.7717/peerj.19619 (PMC12357545; doi:10.7717/peerj.19619)

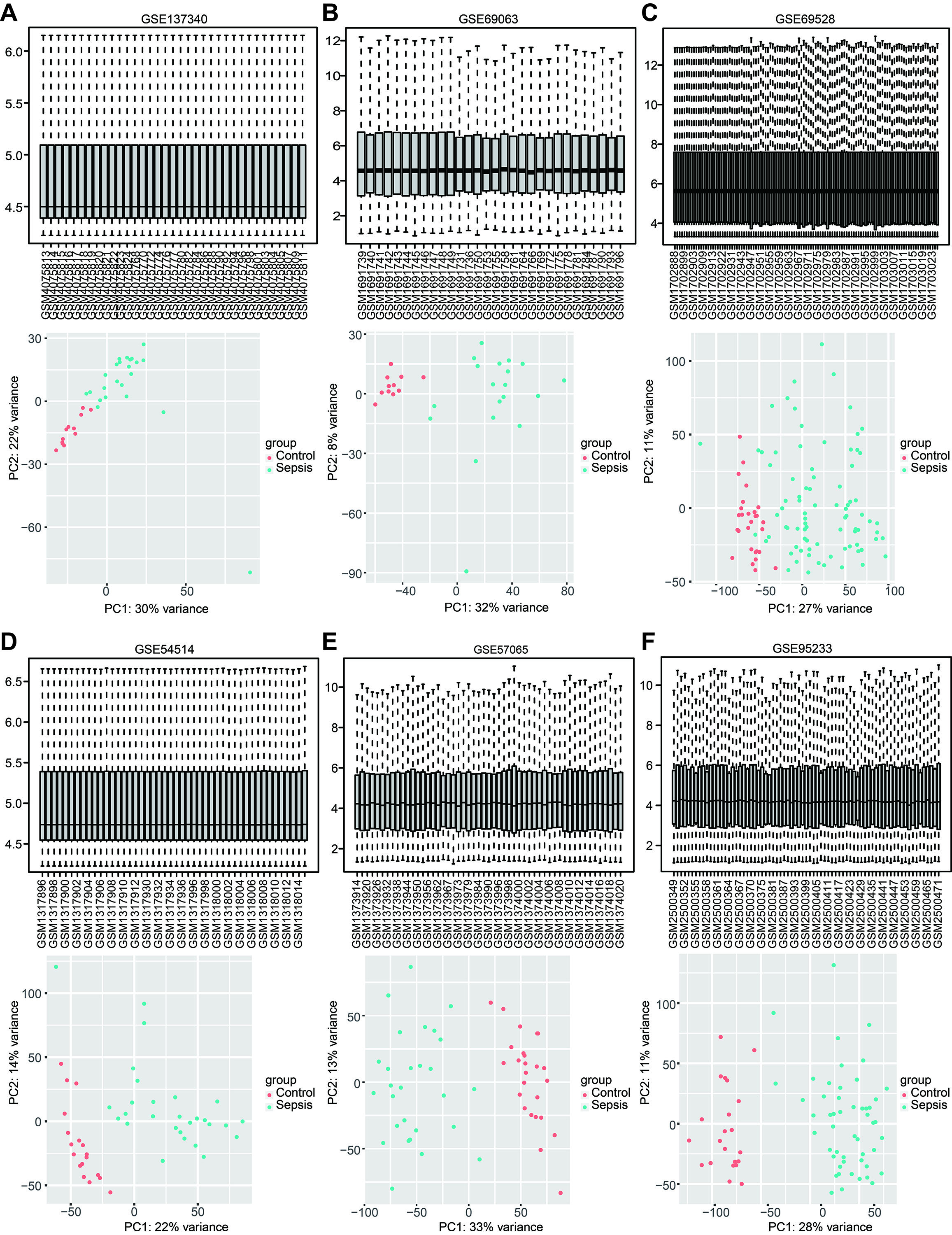

Supplement: Supplemental Information 1 — (A–F) Boxplots displaying the standardized gene expression profiles in each dataset (A) GSE137340, (B) GSE69063, (C) GSE69528, (D) GSE54514, (E) GSE57065 and (F) GSE95233. Scatter plots of principal component analysis revealed significant differences between patients with sepsis and healthy controls. [file peerj-13-19619-s001.jpg]

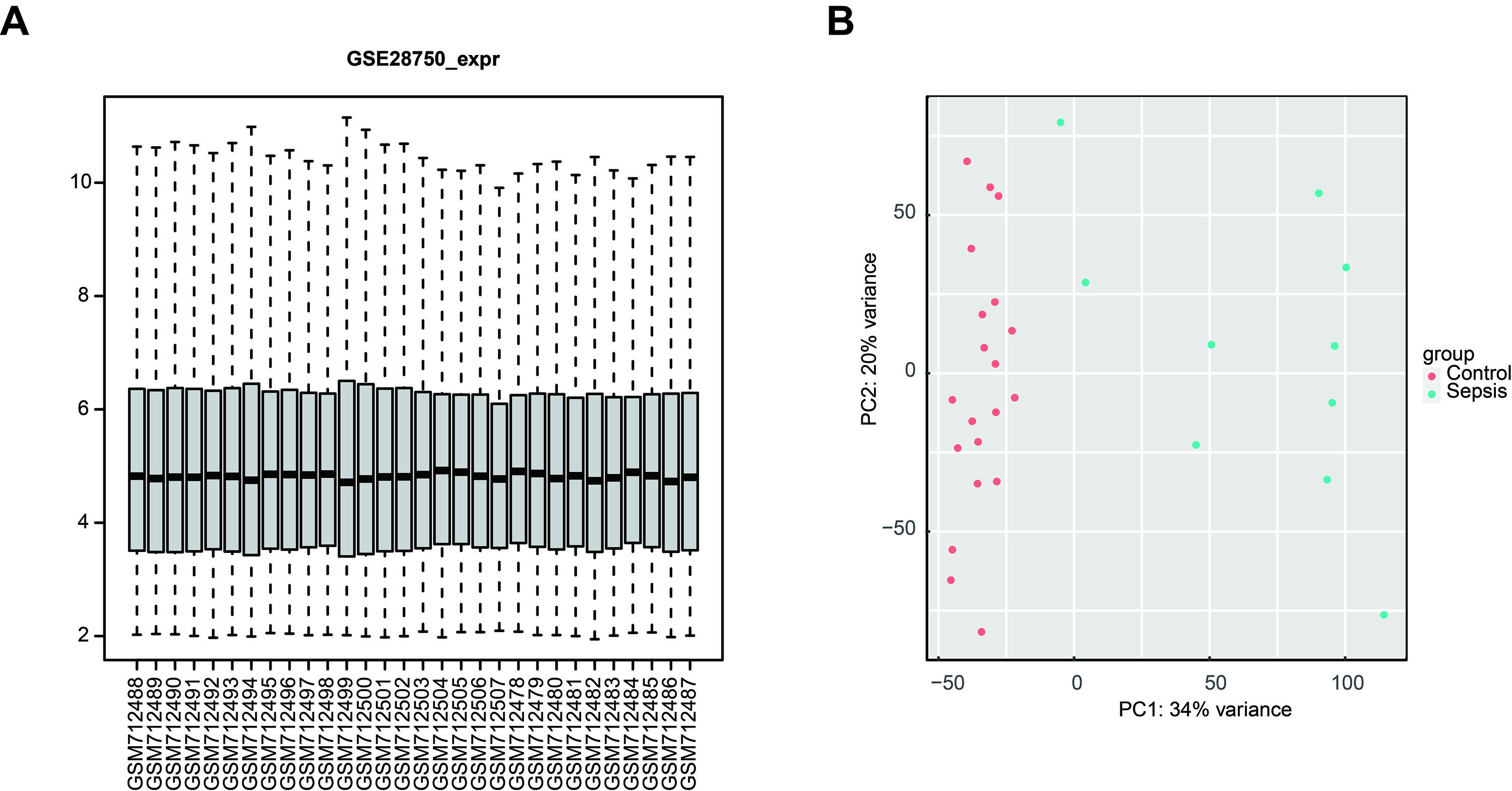

Supplement: Supplemental Information 2 — The mRNA expression of ELF1 in peripheral blood monocytes from (A) patients with sepsis and healthy controls and (B) mice with cecal ligation and puncture and sham controls was detected using reverse transcription-quantitative PCR. [file peerj-13-19619-s002.jpg]

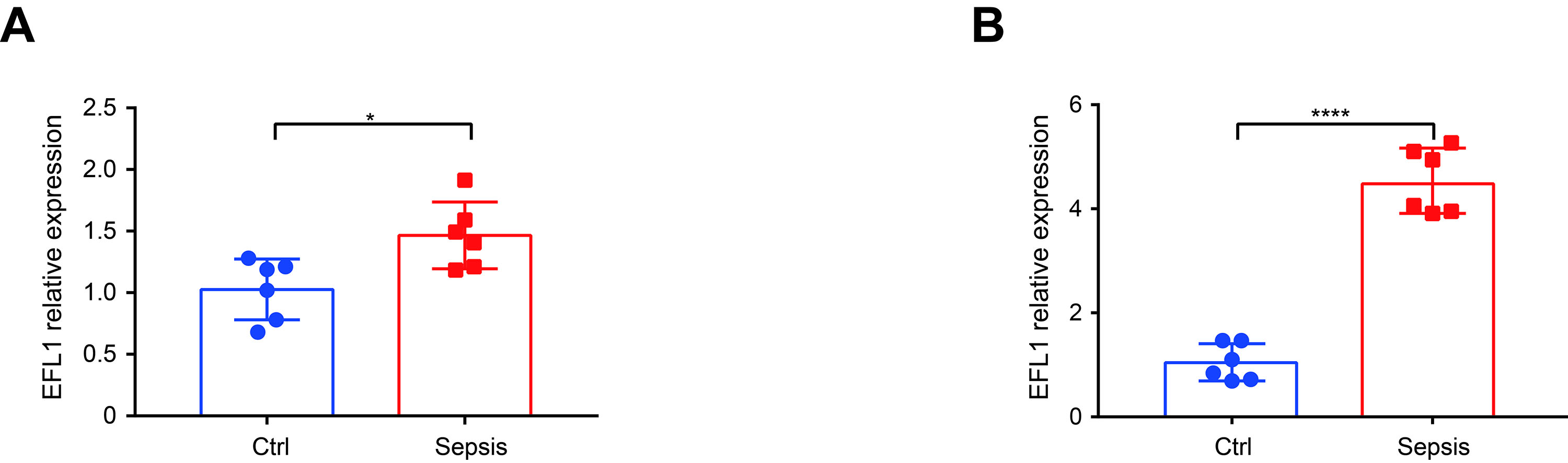

Supplement: Supplemental Information 3 — (A) Boxplot showing the standardized gene expression profiles in the GSE28570 validation dataset. (B) Principal component analysis scatter plot showing significant differences between patients with sepsis and healthy controls in the GSE28570 validation dataset. [file peerj-13-19619-s003.jpg]
