# Supplementary material for: Identification of hub genes and prediction of the ceRNA network in adult sepsis
Source: PeerJ. 2025 Aug 13;13:e19619. doi: 10.7717/peerj.19619 (PMC12357545; doi:10.7717/peerj.19619)
Supplement: Supplemental Information 11 [file peerj-13-19619-s011.docx]

R.version

.libPaths()

getwd()

rm(list = ls())

library(tidyverse)

#### 0 GSEs, GSEs_sm, GSE_expr, GSE_pd ----

setwd("F:\\HelixCourses\\GEOdatasets_spsis1\\DEG_SPSIS")

GSEs <- c("GSE137340",

"GSE137341",

"GSE100159",

"GSE33118",

"GSE9960",

"GSE69063",

"GSE69528",

"GSE54514")

GSE_sm <- paste0(GSEs, "_sm")

GSE_expr <- paste0(GSEs, "_expr")

GSE_pd <- paste0(GSEs, "_pd")

#if (!requireNamespace("BiocManager", quietly = TRUE))

# install.packages("BiocManager")

#BiocManager::install("GEOquery")

library(GEOquery)

for (i in 1:8) {

assign(GSE_sm[i], getGEO(GEO = GSEs[i],

filename = paste0(GSEs[i], "_series_matrix.txt.gz"),

getGPL = F))

assign(GSE_expr[i], exprs(get(GSE_sm[i])))

assign(GSE_pd[i], pData(get(GSE_sm[i])))

print(i)

}

save.image(file='.//alldata.Rdata')

#load("alldata.Rdata")

save(GSE137340_expr, GSE137340_pd, GSE137340_sm, file = "./GSE137340/GSE137340.Rda")

save(GSE100159_expr, GSE100159_pd, GSE100159_sm, file = "./GSE100159/GSE100159.Rda")

save(GSE33118_expr, GSE33118_pd, GSE33118_sm, file = "./GSE33118/GSE33118.Rda")

save(GSE9960_expr, GSE9960_pd, GSE9960_sm, file = "./GSE9960/GSE9960.Rda")

save(GSE69063_expr, GSE69063_pd, GSE69063_sm, file = "./GSE69063/GSE69063.Rda")

save(GSE69528_expr, GSE69528_pd, GSE69528_sm, file = "./GSE69528/GSE69528.Rda")

save(GSE54514_expr, GSE54514_pd, GSE54514_sm, file = "./GSE54514/GSE54514.Rda")

pdf("./figures/Boxplots.PDF", 15, 6)

for (i in 1:8) {

boxplot(get(GSE_expr[i]),

las=2,

outline=FALSE,

main = paste0("Boxplot for ", GSEs[i]))

}

dev.off()

#### 1 GSE137340 ----

### load data

#view(GSE137340_pd)

#fix(GSE137340_pd)

#dir.create("GSE137340")

rm(list = ls())

library(tidyverse)

setwd("F:\\HelixCourses\\GEOdatasets_spsis1\\DEG_SPSIS\\GSE137340")

getwd()

load("GSE137340.Rda")

### organize target data

# load("./GSE137340.Rda")

# write.table(GSE137340_pd, file = "GSE137340_pd_1.txt",sep = "\t",row.names = F,col.names = T,quote = F)

# write.csv(GSE137340_pd, file = "GSE137340_pd_1.csv",sep = "\t",row.names = F,col.names = T,quote = F)

# write.csv(GSE137340_pd, file = "GSE137340_pd_2.csv",quote = F)

## Extract target GSE targets

GSE137340_targets <- GSE137340_pd %>%

dplyr::select(geo_accession, source_name_ch1) %>%

separate(source_name_ch1, into = paste0("x", 1:3), sep = ",", remove = F) %>%

mutate(sample_id = geo_accession,

# sample_name = str_c(x1, x2, sep = "_"),

sample_name = x1,

patiend_id = str_c(x2,str_extract(x1, "\\d+"),sep = "_"),

Day = str_sub(source_name_ch1,-5,-1),

# group = if_else(str_detect(source_name_ch1, 8, 8) %in% c("C", "H"),"Control", "NAFLD"),

group = if_else(str_detect(source_name_ch1, "Healthy"),"Control", "Sepsis")) %>%

filter(!str_detect(source_name_ch1,"Day 2")) %>%

arrange(group, sample_id) %>%

dplyr::select(sample_id:group)

GSE137340_targets$Day <- gsub("althy","Healthy", GSE137340_targets$Day)

dim(GSE137340_expr)

GSE137340_expr <- GSE137340_expr[,GSE137340_targets$sample_id]

# fix(GSE137340_targets)

### check qualifty

source("F:/HelixCourses/GEOdatasets_spsis1/DEG_SPSIS/Custom_Functions.R")

boxplot(GSE137340_expr, las=2, outline=FALSE, main = "GSE137340")

PCA_new(GSE137340_expr,

ntop = nrow(GSE137340_expr),

# ntop = 500,

# show_name = T,

group = GSE137340_targets$group)

dev.off()

# RMA normalized: the standardization of the original data

GSE137340_sm@phenoData@data$data_processing[1]

# GSE137340_pd$data_processing[1] #

library(limma)

GSE137340_expr_n = normalizeBetweenArrays(GSE137340_expr)

dim(GSE137340_expr_n)

if(!dir.exists("figures")) dir.create("figures")

pdf('./figures/GSE137340_normalize_norm_boxplot.pdf',width = 8,height = 8)

boxplot(GSE137340_expr_n,

outline=FALSE,

#notch=T,

#col=group_list,

main = "GSE137340",

las=2)

dev.off()

pdf('./figures/GSE137340_normalize_norm_PCA_new.pdf',width = 8,height = 8)

PCA_new(GSE137340_expr_n,

ntop = 1000,

# show_name = T,

group = GSE137340_targets$group)

dev.off()

range(GSE137340_expr_n)

# exp <- log2(GSE137340_expr+1)

# range(GSE137340_expr)

### annotation

# BiocManager::install("illuminaHumanv4.db",force = TRUE)

library(illuminaHumanv4.db)

ls("package:illuminaHumanv4.db") #query

ls(illuminaHumanv4.db)

keytypes(illuminaHumanv4.db)

# View annotation data

GSE137340_probe2symbol <- as.data.frame(illuminaHumanv4SYMBOL)

# GSE137340_probe2symbol = toTable(illuminaHumanv4SYMBOL)

head(GSE137340_probe2symbol)

GSE137340_anno <- GSE137340_expr_n %>%

as.data.frame() %>%

dplyr::select(GSE137340_targets$sample_id) %>%

rownames_to_column(var = "probe_id") %>%

inner_join(GSE137340_probe2symbol, ., by = "probe_id") %>%

dplyr::select(-1) %>%

as.data.frame() %>%

aggregate(.~ symbol, data = ., mean) %>%

column_to_rownames(var = "symbol")

# View annotation results

GSE137340_anno[1:6,1:6]

dim(GSE137340_expr_n)

dim(GSE137340_anno)

## save data

save(GSE137340_expr_n, GSE137340_targets, GSE137340_anno, file = "GSE137340_processsed.Rda")

library(limma)

group <- factor(GSE137340_targets$group, levels = c("Control", "Sepsis"))

expr <- GSE137340_anno

contrast <- paste0(rev(levels(group)), collapse = "-")

contrast

design <- model.matrix( ~ 0 + group)

colnames(design)

colnames(design) <- levels(group)

contrast.matrix <- makeContrasts(contrast, levels = design)

contrast.matrix

## arrayWeights

aw <- arrayWeights(expr, design = design)

aw

barplot(aw)

fit <- lmFit(expr, design, weights = aw)

fit <- contrasts.fit(fit, contrast.matrix)

fit <- eBayes(fit)

GSE137340_DEG <- topTable(fit, coef = 1, n = Inf) %>%

rownames_to_column(var = "symbol")

#head(GSE137340_DEG)

GSE137340_DEG_p <- GSE137340_DEG %>%

dplyr::filter(abs(logFC) > 1.2, P.Value < 0.05)

head(GSE137340_DEG_p)

# setwd("F:\\HelixCourses\\GEOdatasets_spsis1\\DEG_SPSIS\\GSE137340")

save(GSE137340_DEG, GSE137340_DEG_p, file = "./GSE137340_DEG.Rda")

# write.table(GSE137340_DEG, file = "GSE137340_DEG.txt",sep = "\t",row.names = F,col.names = T,quote = F)

# write.table(GSE137340_DEG_p, file = "GSE137340_DEG_p.txt",sep = "\t",row.names = F,col.names = T,quote = F)

##

# plot_df

plot_df <- GSE137340_DEG

# Define volcano plot thresholds

plot_df$threshold = factor(ifelse(plot_df$P.Value < 0.05 & abs(plot_df$logFC) > 1.2,

ifelse(plot_df$logFC > 1.2 ,'Up','Down'),'NS'),

levels=c('Up','Down','NS'))

#

library(ggplot2)

pdf('./figures/Volcano_GSE137340.pdf',width = 8,height = 8)

ggplot(plot_df, aes(y = logFC, x = -log10(P.Value), color = threshold)) +

geom_point(alpha = 0.8) +# point size

scale_color_manual(values = c("#DC143C", "#143cdc", "#808080")) +# point color

xlab('-log10 (P-value)') +

ylab('log2 (FoldChange)') +

ggtitle("Volcano Plot") +#

geom_hline(yintercept = c(-1.2, 1.2), lty = 3, col = "black", lwd = 0.5) +

geom_vline(xintercept = -log10(0.05), lty = 3, col = "black", lwd = 0.5) +#

theme_bw() +

theme(legend.title = element_blank(),

legend.position = "none",

plot.title = element_text(hjust = 0.5))

dev.off()

p1 <- ggplot(plot_df, aes(y = logFC, x = -log10(P.Value), color = threshold)) +

geom_point(alpha = 0.8) +# point size

scale_color_manual(values = c("#DC143C", "#143cdc", "#808080")) +# point color

xlab('-log10 (P-value)') +

ylab('log2 (FoldChange)') +

ggtitle("Volcano Plot") +

geom_hline(yintercept = c(-1.2, 1.2), lty = 3, col = "black", lwd = 0.5) +

geom_vline(xintercept = -log10(0.05), lty = 3, col = "black", lwd = 0.5) +

theme_bw() +

theme(legend.title = element_blank(),

legend.position = "none",

plot.title = element_text(hjust = 0.5))

p1

dev.off()

save(p1, file = "p1.Rda")

#### 2 GSE69063 ----

rm(list = ls())

### load data

setwd("F:\\HelixCourses\\GEOdatasets_spsis1\\DEG_SPSIS\\GSE69063")

getwd()

load_input <- load("GSE69063.Rda")

load_input

library(tidyverse)

view(GSE69063_pd)

#fix(GSE69063_pd)

setwd("F:\\HelixCourses\\GEOdatasets_spsis1\\DEG_SPSIS")

#dir.create("GSE69063")

if(!dir.exists("GSE69063")) dir.create("GSE69063")

setwd("F:\\HelixCourses\\GEOdatasets_spsis1\\DEG_SPSIS\\GSE69063")

getwd()

### organize target data

GSE69063_targets <- GSE69063_pd %>% dplyr::select(2, 1) %>%

separate(title, into = paste0("x", 1:3), sep = ",", remove = F) %>%

filter(!str_detect(title,"Day 2")) %>%

filter(!str_detect(title,"Day 4")) %>%

filter(!str_detect(title,"Day 5")) %>%

mutate(sample_id = geo_accession,# add several columns

#file_name = paste0(sample_id, ".CEL.gz"),

#sample_name = title,

sample_name = str_c(x1, x2, sep = "_"),# Join multiple strings into a single string.

patient_id = x2,

# patient_id = str_match(x2, "\\d")[, 2],

# strings match the first character

# and see the computer 360 browser collection folder (matching characters)

# and see (13_Reading Affymetrix chip data.R) code: P1455

#str_sub----Extract and replace substrings from a character vector. Extract the substring from the last character

#GSE_num = "GSE69063",

group = if_else(str_detect(title, "Healthy"),"Control", "Sepsis")) %>%

dplyr::select(sample_id:group)

GSE69063_expr <- GSE69063_expr[,GSE69063_targets$sample_id] %>% as.data.frame()

dim(GSE69063_expr)

## View the scope of the data

range(GSE69063_expr)

# exp <- log2(GSE9960_expr+1)

# range(GSE9960_expr)

if(!dir.exists('figures'))dir.create('figures')

### Check qualifty

## boxplot

boxplot(GSE69063_expr, las=2, outline=FALSE,

# col = as.factor(GSE69063_targets$group),

main = "GSE69063_expr")

dev.off()

source("F:/HelixCourses/GEOdatasets_spsis1/DEG_SPSIS/Custom_Functions.R")

PCA_new(GSE69063_expr,

ntop = nrow(GSE69063_expr),

# ntop = 500, #the number of top-ranked genes

# show_name = T,

group = GSE69063_targets$group)

dev.off()

#### 3 GSE69528 ----

rm(list = ls())

### load data

setwd("F:\\HelixCourses\\GEOdatasets_spsis1\\DEG_SPSIS\\GSE69528")

getwd()

load_input <- load("GSE69528.Rda")

load_input

library(tidyverse)

view(GSE69528_pd)

#fix(GSE69528_pd)

### create new folder

setwd("F:\\HelixCourses\\GEOdatasets_spsis1\\DEG_SPSIS")

#dir.create("GSE69528")

if(!dir.exists("GSE69528")) dir.create("GSE69528")

setwd("F:\\HelixCourses\\GEOdatasets_spsis1\\DEG_SPSIS\\GSE69528")

getwd()

### Collate target data

GSE69528_targets <- GSE69528_pd %>% dplyr::select(2, 1, 8) %>% dplyr::select(1, 2) %>%

separate(title, into = paste0("x", 1:7), sep = " ", remove = F) %>%

filter(!str_detect(title,"Day 2")) %>%

mutate(sample_id = geo_accession,# add several columns

#file_name = paste0(sample_id, ".CEL.gz"),

#sample_name = title,

sample_name = str_c(x1, x2, sep = "_"),# Join multiple strings into a single string.

patient_id = x7,

#str_sub----Extract and replace substrings from a character vector. Extract the substring from the last character

#GSE_num = "GSE69063",

group = if_else(str_detect(title, "Healthy"),"Control", "Sepsis")) %>%

dplyr::select(sample_id:group) %>%

arrange(group, sample_id) %>%

dplyr::select(2,3,4,1) %>% arrange(group,sample_id)

GSE69528_expr <- GSE69528_expr[,GSE69528_targets$sample_id] %>% as.data.frame()

## View the scope of the data

range(GSE69528_expr)

# exp <- log2(GSE69528_expr+1)

# range(GSE69528_expr)

if(!dir.exists("figures")) dir.create("figures")

## checkout quality of data

pdf('./figures/boxplot_GSE69528.pdf')

boxplot(GSE69528_expr,

las=2,

outline=FALSE,

main = "GSE69528_expr")

dev.off()

source("F:/HelixCourses/GEOdatasets_spsis1/DEG_SPSIS/Custom_Functions.R")

PCA_new(GSE69528_expr,

ntop = nrow(GSE69528_expr),

# ntop = 500, #the number of top-ranked genes

# show_name = T,

group = GSE69528_targets$group)

dev.off()

## View the original data processing method

GSE69528_sm@phenoData@data$data_processing[1]

# GSE69528_pd$data_processing[1] # This code is equivalent to the above code

#### 4 GSE54514 ----

### 4.4 GSE54514 ----

#### 1 GSE54514 data import

### 1.1 load data

###control group NO.1 day VS sepsis group NO.3 day

rm(list=ls())

setwd("F:\\HelixCourses\\GEOdatasets_spsis1\\DEG_SPSIS\\GSE54514")

getwd()

### load data

load_input <- load("GSE54514.Rda")

load_input

library(tidyverse)

### Since PCA analysis shows that the sepsis group and the control group cannot be well separated,

### we do not use the expression matrix extracted from the Series Matrix, but read the original data to remove batch effects

## Read the original data expression matrix

# See 1: F:/HelixCourses/Full Code Breakthrough in Bioinformatics Data Mining/Part 2 of the Bioinformatics System Course/Level 1/L51_Codes/NAFLD_6Datasets_DEG.R Code: P160~P173

# See 2: F:/HelixCourses/Full Code Breakthrough in Bioinformatics Data Mining/Part 2 of the Bioinformatics System Course/Level 1/L51_Codes/13_Reading Affymetrix Chip Data.R Code: P12~P111

library(oligo)

# In the oligo package, the read.celfiles function reads the CEL file:

# obtain the original file list

cel_files <- list.celfiles("F:/HelixCourses/GEOdatasets_spsis1/DEG_SPSIS/GSE54514/GSE54514_RAW/",

full.name = T,

# full.name = F,

listGzipped = T)

cel_files_1 <- ifelse(str_match(cel_files,"GSM\\d+") %in% GSE54514_targets$sample_id,TRUE,FALSE)

cel_files_2 <- cel_files[cel_files_1]

cel_files1 <- list.celfiles("F:/HelixCourses/GEOdatasets_spsis1/DEG_SPSIS/GSE54514/GSE54514_RAW/",

# full.name = T,

full.name = F,

listGzipped = T)

cel_files_11 <- ifelse(str_match(cel_files1,"GSM\\d+") %in% GSE54514_targets$sample_id,TRUE,FALSE)

cel_files_21 <- cel_files1[cel_files_11]

identical(cel_files_1,cel_files_11)

GSE54514_targets <- GSE54514_targets %>% mutate(FileName = cel_files_2) %>%

relocate(FileName,everything())

## new cel: read the original file to obtain the ExpressionFeatureset

GSE54514_cel <- read.celfiles(filenames = GSE54514_targets$FileName,

phenoData = AnnotatedDataFrame(data = GSE54514_targets),

sampleNames = GSE54514_targets$sample_id)

# See F:/HelixCourses/Full Code Breakthrough in Bioinformatics Data Mining/Part 2 of the Bioinformatics System Course/Level 1/L51_Codes/NAFLD_6Datasets_DEG.R Code: P166

GSE54514_cel1 <- read.celfiles(filenames = paste0("GSE54514_RAW/", cel_files_21))

identical(GSE54514_cel,GSE54514_cel1)

a <- exprs(GSE54514_cel) %>% as.data.frame()

b <- exprs(GSE54514_cel1) %>% as.data.frame()

head(a)[,1:6]

head(b)[,1:6]

identical(a,b)

## Lookup batch effect: The first way to extract batch effect

cel <- GSE54514_cel

cel_pd <- pData(cel)

cel@protocolData@data$dates

cel_dates <- str_split(cel@protocolData@data$dates, pattern = "\\s+", simplify = T)[,1]

# \s matches any whitespace character, and is equivalent to [ \f\n\r\t\v]

class(cel_dates)

table(cel_dates) # Check batches

## Lookup batch effect: The second way to extract batch effect

library(sva)

library(tidyverse)

# Chip scanning date can be considered as an important batch effect factor

GSE54514_targets_batch <- GSE54514_targets

cel_dates1 <- cel@protocolData@data$dates %>%

str_sub(1, 8)

identical(cel_dates,cel_dates1)

GSE54514_targets_batch$batch <- cel@protocolData@data$dates %>%

str_sub(1, 8)# Extract the first 8 characters

## save data

save(GSE54514_cel,GSE54514_targets_batch,

file = ".\\GSE54514_cel.Rda")

## Remove batch effect

## PCA analysis shows that the normal group and the sepsis group have batch effects,

## so the original data is read to remove batch effects

## 4.4.1 Do not include grouping factors

mod <- model.matrix(~1, data= GSE54514_targets_batch)

GSE54514_combat <- ComBat(dat = GSE54514_expr,

batch = GSE54514_targets_batch$batch,

mod = mod,

par.prior=TRUE, # Use parameter estimation, false for non-parametric estimation which is more time-consuming

prior.plots=FALSE)

# ## 4.4.2 Include grouping factors, tell the algorithm what grouping factors we are interested in (It turns out that adding grouping factors is useless)

# mod1 <- model.matrix(~ as.factor(group), data= GSE54514_targets_batch)

# mod2 <- model.matrix(~1 + group, data= GSE54514_targets_batch)

# identical(mod1,mod2)

# GSE54514_combat1 <- ComBat(dat = GSE54514_expr,

# batch = GSE54514_targets_batch$batch,

# mod = mod1,

# par.prior=TRUE,

# prior.plots=FALSE)

## Check the effect of removing batch effects

# boxplot after batch

boxplot(GSE54514_expr,

# ylab = expression(log[2](intensity)),

ylab = expression(intensity),

las = 2,

outline = FALSE)

boxplot(GSE54514_combat,

# ylab = expression(log[2](intensity)),

ylab = expression(intensity),

las = 2,

outline = FALSE)

### PCA after batch

PCA_new(GSE54514_combat,

ntop = nrow(GSE54514_combat),

# show_name = T,

group = GSE54514_targets_batch$group)

dev.off()

# #### After the above analysis, it was found that the data obtained by reading the original data and removing batch effects is the most reasonable,

# so this part of the data is used for subsequent analysis ----

### annotation

GSE54514_sm@annotation

## GPL570

library(hgu133plus2.db)

library(illuminaHumanv3.db)

ls("package:hgu133plus2.db")

p2s <- toTable(hgu133plus2SYMBOL) #%>%

# set_names("probe_id", "symbol")# rename

head(p2s)

## Custom annotation function

annotate_expr <- function(expr, p2s, fun = mean){# Define three parameters, the column names of p2s are fixed

library(dplyr)

library(tibble)

# expr

expr <- expr %>%

as.data.frame() %>%

tibble::rownames_to_column("probe_id")# Change the row name to a column called "probe_id"

# annotate

expr_annotated <- p2s %>%

dplyr::inner_join(expr, by = "probe_id") %>% # Take the intersection of expr and p2s

na.omit() %>% # Remove NAs

dplyr::select(-probe_id) %>% # Remove the "probe_id" column

stats::aggregate(. ~ symbol, data =., FUN = fun) %>% # For multiple probes corresponding to the same symbol, take the mean of these probes

tibble::column_to_rownames("symbol")# Change the "symbol" column to the row name

return(expr_annotated)# Return this expr_annotated, and then assign it to annotate_expr

}

## Annotation

GSE54514_anno <- annotate_expr(GSE54514_expr, p2s)

head(GSE54514_anno)[, 1:3]

GSE54514_expr_n <- GSE54514_expr # This step is to keep all data sets consistent

## save data

save(GSE54514_expr_n, GSE54514_targets, GSE54514_anno,

file = ".\\GSE54514_processed.Rda")

### Differential analysis DEGs

library(limma)

## Load chip data and define groups

group <- factor(GSE54514_targets$group, levels = c("Control", "Sepsis")) # Use factor to define the grouping group

expr <- GSE54514_anno

contrast <- paste0(rev(levels(group)), collapse = "-")

contrast

## Create design matrix

design <- model.matrix( ~ 0 + group)

colnames(design)

colnames(design) <- levels(group)

## Create contrast matrix

contrast.matrix <- makeContrasts(contrast, levels = design)

contrast.matrix

## Use limma for differential analysis

fit <- lmFit(expr, design)

fit <- contrasts.fit(fit, contrast.matrix)

fit <- eBayes(fit)

## Check all results

results <- summary(decideTests(fit, lfc = 1.2))

results

## For design

design <- model.matrix(~ group, data = GSE54514_targets)

colnames(design)

colnames(design) <- c("Control", "SepsisvsControl")

head(design)

## arrayWeights

expr <- GSE54514_anno

aw <- arrayWeights(expr, design = design)

aw

barplot(aw)

## Use limma for differential analysis

fit <- lmFit(expr, design, weights = aw)

fit <- contrasts.fit(fit, contrast.matrix)

fit <- eBayes(fit)

## DEG results

results <- summary(decideTests(fit, lfc = 1.2))

results

# number = Inf: all differential analysis results; SepsisvsControl: represents the type to be compared

res_DEG <- topTable(fit, coef = "SepsisvsControl", number = Inf)

# res_DEG_sig <- topTable(fit, coef = "SepsisvsControl", n = Inf, p.value = 0.05, lfc = 1)# Here p is the corrected p

res_DEG_sig <- topTable(fit, coef = "SepsisvsControl", n = Inf, p.value = 0.05, lfc = 1.2)

GSE54514_DEG1 <- topTable(fit, coef = 2, n = Inf)

identical(res_DEG, GSE54514_DEG1)

GSE54514_DEG <- topTable(fit, coef = 2, n = Inf) %>% # coef = 1 when there is no contrast.matrix()

rownames_to_column(var = "symbol")

# Filter for |logFC|>1.2 and p<0.05 results

GSE54514_DEG_p <- GSE54514_DEG %>%

dplyr::filter(abs(logFC) > 1.2 , P.Value < 0.05)

### save data

save(GSE54514_DEG, GSE54514_DEG_p,

file = ".\\GSE54514_DEG.Rda")

### Draw volcano plot

## plot_df

plot_df <- GSE54514_DEG

# Define volcano plot thresholds

plot_df$threshold = factor(ifelse(plot_df$P.Value < 0.05 & abs(plot_df$logFC) > 1.2,

ifelse(plot_df$logFC > 1.2 ,'Up','Down'),'NS'),

levels=c('Up','Down','NS'))

# Start plotting

library(ggplot2)

pdf('./figures/Volcano_GSE54514.pdf',width = 8,height = 8)

ggplot(plot_df, aes(y = logFC, x = -log10(P.Value), color = threshold)) +

geom_point(alpha = 0.8) +# point size

scale_color_manual(values = c("#DC143C", "#143cdc", "#808080")) +# point color

xlab('-log10 (P-value)') +

ylab('log2 (FoldChange)') +

ggtitle("Volcano Plot") +

geom_hline(yintercept = c(-1.2, 1.2), lty = 3, col = "black", lwd = 0.5) +

geom_vline(xintercept = -log10(0.05), lty = 3, col = "black", lwd = 0.5) +

theme_bw() +

theme(legend.title = element_blank(),

legend.position = "none",

plot.title = element_text(hjust = 0.5))

dev.off()

p4 <- ggplot(plot_df, aes(y = logFC, x = -log10(P.Value), color = threshold)) +

geom_point(alpha = 0.8) +# point size

scale_color_manual(values = c("#DC143C", "#143cdc", "#808080")) +# point color

xlab('-log10 (P-value)') +

ylab('log2 (FoldChange)') +

ggtitle("Volcano Plot") +

geom_hline(yintercept = c(-1.2, 1.2), lty = 3, col = "black", lwd = 0.5) +

geom_vline(xintercept = -log10(0.05), lty = 3, col = "black", lwd = 0.5) +

theme_bw() +

theme(legend.title = element_blank(),

legend.position = "none",

plot.title = element_text(hjust = 0.5))

p4

dev.off()

save(p4, file = "./p4.Rda")

#### 5 GSE57065----

### 1 load packages

# rm(list = ls())

library(GEOquery)

library(readxl)

library(tidyverse)

### 2 Load data

setwd("F:\\HelixCourses\\GEOdatasets_spsis1\\DEG_SPSIS")

getwd()

GSE57065_sm<- getGEO(filename = "GSE57065_series_matrix.txt.gz",

destdir = ".",

getGPL = F )

GSE57065_expr <- exprs(GSE57065_sm)

head(GSE57065_expr)[, 1:6]

GSE57065_pd <- pData(GSE57065_sm)

### Create file

# dir.create("GSE57065")

if(!dir.exists("GSE57065"))dir.create("GSE57065")

setwd("F:\\HelixCourses\\GEOdatasets_spsis1\\DEG_SPSIS\\GSE57065")

getwd()

### Save data

save(GSE57065_sm,GSE57065_expr,GSE57065_pd,

file = ".\\GSE57065.Rda")

# load(".\\GSE57065_data.Rda")

### Organize target data

GSE57065_targets <- GSE57065_pd %>% dplyr::select(2, 1, 13) %>% dplyr::select(1, 2) %>%

separate(title, into = paste0("x", 1:3), sep = "_", remove = F) %>%

mutate(sample_id = geo_accession,# add several columns

#file_name = paste0(sample_id, ".CEL.gz"),

#sample_name = title,

sample_name = str_c(x1, x2, sep = "_"),# Join multiple strings into a single string.

patient_id = x2,

#str_sub----Extract and replace substrings from a character vector. Extract the substring from the last character

#GSE_num = "GSE69063",

group = if_else(str_sub(x2,1,1) == "P","Sepsis","Control")) %>%

filter(!(x3 %in% c("H24", "H48"))) %>% # "F:/HelixCourses/R Language from Scratch to Mastery_7 days/Day 3/Section 3 Live Broadcast" Code: P264

dplyr::slice(-c(1:10)) %>%

dplyr::slice(-c(31:42)) %>%

arrange(group, sample_id) %>%

dplyr::select(sample_id:group)

GSE57065_expr <- GSE57065_expr[,GSE57065_targets$sample_id] %>% as.data.frame()

dim(GSE57065_expr)

## View Data

range(GSE57065_expr)

# exp <- log2(GSE57065_expr+1)

# range(GSE57065_expr)

### Evaluate data

if(!dir.exists("figures")) dir.create("figures")

# boxplot(GSE29450_rma, las = 2, col = rep(c("blue", "red"), each = 10))

# boxplot(GSE57065_expr, las=2, outline=FALSE,

# col = rep(c("blue", "red"), c(22,25)),

# main = "GSE57065_expr")

# Below code see F:/HelixCourses/Full Code Breakthrough in Bioinformatics Data Mining/Part 2 of the Bioinformatics System Course/Level 1/L51_Codes/32_batch_effect_data2.R Code: P29~P32

# boxplot(GSE57065_expr, las=2, outline=FALSE,

# col = rep(c("blue", "red"), c(22,25)), outline = F)

boxplot(GSE57065_expr,

# ylab = expression(log[2](intensity)),

ylab = expression(intensity),

las = 2,

outline = FALSE)

dev.off()

source("F:\\HelixCourses\\GEOdatasets_spsis1\\DEG_SPSIS\\Custom_Functions.R")

targets <- GSE57065_targets

PCA_new(GSE57065_expr, show_name = F)

dev.off()

# View the original data processing method

GSE57065_sm@phenoData@data$data_processing[1]

# GSE57065_pd$data_processing[1] # This code is equivalent to the above code

## save data

# save(GSE57065_expr,GSE57065_targets,

# file = ".\\GSE57065_processed.Rda")

### 5 remove batch effect

## PCA analysis shows that the normal group and the sepsis group have batch effects,

## so the original data is read to remove batch effects

## Use oligo package to read CEL files

# See 1: F:/HelixCourses/Full Code Breakthrough in Bioinformatics Data Mining/Part 2 of the Bioinformatics System Course/Level 1/L51_Codes/NAFLD_6Datasets_DEG.R Code: P160~P173

# See 2: F:/HelixCourses/Full Code Breakthrough in Bioinformatics Data Mining/Part 2 of the Bioinformatics System Course/Level 1/L51_Codes/13_Reading Affymetrix Chip Data.R Code: P12~P111

library(oligo)

cel_files <- list.celfiles("F:/HelixCourses/GEOdatasets_spsis1/DEG_SPSIS/GSE57065/GSE57065_RAW/",

full.name = T,

# full.name = F,

listGzipped = T)

# # str_match returns multiple column matching results, the first column is the overall matching result,

# # the following columns are the matching results for each group.

# c <- str_match(cel_files,"GSM\\d+")[,1] # Extract the vector

# c1 <- str_match(cel_files,"GSM\\d+") # Extract the list

# c2 <- str_match(cel_files,"GSM\\d+")[,2]

# d <- str_match(GSE54514_targets_2$x4, "\\d+")[, 1]

# d <- str_match(GSE54514_targets_2$x4, "\\d+")[, 2]

cel_files_1 <- ifelse(str_match(cel_files,"GSM\\d+") %in% GSE57065_targets$sample_id,TRUE,FALSE)

cel_files <- cel_files[cel_files_1]

cel_files1 <- list.celfiles("F:/HelixCourses/GEOdatasets_spsis1/DEG_SPSIS/GSE57065/GSE57065_RAW/",

# full.name = T,

full.name = F,

listGzipped = T)

cel_files_11 <- ifelse(str_match(cel_files1,"GSM\\d+") %in% GSE57065_targets$sample_id,TRUE,FALSE)

cel_files1 <- cel_files1[cel_files_11]

identical(str_match(cel_files,"GSM\\d+")[,1],GSE57065_targets$sample_id)

identical(cel_files_1,cel_files_11)

GSE57065_targets_FileName <- GSE57065_targets %>% mutate(FileName = cel_files) %>%

relocate(FileName,everything())

## new cel

GSE57065_cel <- read.celfiles(filenames = GSE57065_targets_FileName$FileName,

phenoData = AnnotatedDataFrame(data = GSE57065_targets_FileName),

sampleNames = GSE57065_targets_FileName$sample_id)

# GSE57065_cel1 <- read.celfiles(cel_files) # This reading method only obtains the expression matrix, without target and sampleNames data

## Save raw data

save(GSE57065_cel, file = "./GSE57065_cel.Rdata")

## Lookup batch effect

# cel <- GSE57065_cel

# cel_pd <- pData(cel)

# cel@protocolData@data$dates

# cel_dates <- str_split(cel@protocolData@data$dates, pattern = "\\s+", simplify = T)[,1]

# \s matches any whitespace character, and is equivalent to [ \f\n\r\t\v]

class(cel_dates)

table(cel_dates) # Check batches

# Chip scanning date can be considered as an important batch effect factor

GSE57065_targets_batch <- GSE57065_targets

GSE57065_targets_batch$batch <- GSE57065_cel@protocolData@data$dates %>%

str_sub(1, 8)# Extract the first 8 characters

table(GSE57065_targets_batch$batch)

## 5.9.1 Do not include grouping factors

library(sva)

mod <- model.matrix(~1, data= GSE57065_targets_batch)

batch <- GSE57065_targets_batch$batch

GSE57065_expr <- exprs(GSE57065_cel) %>% as.data.frame()

range(GSE57065_expr)

# GSE57065_expr_rma <- rownames_to_column(GSE57065_expr, "ID")

# GSE57065_expr_rma <- GSE57065_expr_rma[!duplicated(GSE57065_expr_rma$ID), ]

GSE57065_combat <- ComBat(dat = GSE57065_expr,

batch = batch,

mod = mod,

par.prior=TRUE, # Use parameter estimation, false for non-parametric estimation which is more time-consuming

prior.plots=FALSE)

# ## 5.9.2 Include grouping factors, tell the algorithm what grouping factors we are interested in

# mod1 <- model.matrix(~ as.factor(group), data= GSE57065_targets_batch)

# mod2 <- model.matrix(~1 + group, data= GSE57065_targets_batch)

# GSE57065_combat1 <- ComBat(dat = GSE57065_expr,

# batch = batch,

# mod = mod1,

# par.prior=TRUE,

# prior.plots=FALSE)

## Check quality

## boxplot after batch

boxplot(GSE57065_expr ,

# ylab = expression(log[2](intensity)),

ylab = expression(intensity),

las = 2,

outline = FALSE)

boxplot(GSE57065_combat,

# ylab = expression(log[2](intensity)),

ylab = expression(intensity),

las = 2,

outline = FALSE)

dev.off()

## plot

GSE57065_p1 <- PCA_new(GSE57065_expr,

ntop = 10000,

# ntop = nrow(GSE57065_expr), #the number of top-ranked genes

# show_name = T,

group = GSE57065_targets_batch$group )

GSE57065_p1

dev.off()

GSE57065_p2 <- PCA_new(GSE57065_combat,

ntop = 10000,

group = GSE57065_targets_batch$group,

show_name = F)+

ggtitle("PCA before batch effect correction") +

theme(plot.title = element_text(hjust = 0.5))

GSE57065_p2

GSE57065_p3 <- PCA_new(GSE57065_combat1,

ntop = 10000,

group = GSE57065_targets_batch$group,

show_name = T)+

ggtitle("PCA before batch effect correction") +

theme(plot.title = element_text(hjust = 0.5))

GSE57065_p3

dev.off()

library(cowplot)

GSE57065_p123 <- cowplot::plot_grid(GSE57065_p1, GSE57065_p2, GSE57065_p3, nrow = 2)

# pdf("batch/PCA_GSE57065_batch.pdf", height = 7, width = 15)

GSE57065_p123

dev.off()

## save data

# save(GSE57065_combat1,GSE57065_targets_batch,

# file = ".\\GSE57065_batched.Rda")

## Annotation

GSE57065_sm@annotation

## GPL570

library(hgu133plus2.db)

library(illuminaHumanv3.db)

ls("package:hgu133plus2.db")

p2s <- toTable(hgu133plus2SYMBOL) #%>%

# set_names("probe_id", "symbol")# rename

head(p2s)

## Custom annotation function

annotate_expr <- function(expr, p2s, fun = mean){# Define three parameters, the column names of p2s are fixed

library(dplyr)

library(tibble)

# expr

expr <- expr %>%

as.data.frame() %>%

tibble::rownames_to_column("probe_id")# Change the row name to a column called "probe_id"

# annotate

expr_annotated <- p2s %>%

dplyr::inner_join(expr, by = "probe_id") %>% # Take the intersection of expr and p2s

na.omit() %>% # Remove NAs

dplyr::select(-probe_id) %>% # Remove the "probe_id" column

stats::aggregate(. ~ symbol, data =., FUN = fun) %>% # For multiple probes corresponding to the same symbol, take the mean of these probes

tibble::column_to_rownames("symbol")# Change the "symbol" column to the row name

return(expr_annotated)# Return this expr_annotated, and then assign it to annotate_expr

}

## Annotation

GSE57065_expr_anno <- annotate_expr(GSE57065_combat1, p2s)

head(GSE57065_expr_anno)[, 1:3]

## save data

GSE57065_expr_n <- GSE57065_combat1

GSE57065_anno <- GSE57065_expr_anno

save(GSE57065_expr_n, GSE57065_targets, GSE57065_anno,

file = ".\\GSE57065_processed.Rda")

### Differential analysis DEGs

library(limma)

## For design

design <- model.matrix(~ group, data = GSE57065_targets_batch)

colnames(design)

colnames(design) <- c("Control", "SepsisvsControl")

head(design)

## arrayWeights

# if (!requireNamespace("arrayWeights", quietly = TRUE)) {

# BiocManager::install("arrayWeights", ask = F, update = F)

# }

expr <- GSE57065_anno

aw <- arrayWeights(expr, design = design)

aw

barplot(aw)

dev.off()

# fit <- lmFit(expr_anno, design)# Linear fitting

fit <- lmFit(expr, design, weights = aw)

fit <- eBayes(fit)# Bayesian

## DEG results

results <- summary(decideTests(fit, lfc = 1.2))

results

# number = Inf: all differential analysis results; SepsisvsControl: represents the type to be compared

res_DEG <- topTable(fit, coef = "SepsisvsControl", number = Inf)

# res_DEG_sig <- topTable(fit, coef = "SepsisvsControl", n = Inf, p.value = 0.05, lfc = 1)# Here p is the corrected p

res_DEG_sig <- topTable(fit, coef = "SepsisvsControl", n = Inf, p.value = 0.05, lfc = 1.2)

GSE57065_DEG1 <- topTable(fit, coef = 2, n = Inf)

identical(res_DEG, GSE57065_DEG1)

GSE57065_DEG <- topTable(fit, coef = 2, n = Inf) %>% # coef = 1 when there is no contrast.matrix()

rownames_to_column(var = "symbol")

# Filter for |logFC|>1.2 and p<0.05 results

GSE57065_DEG_p <- GSE57065_DEG %>%

dplyr::filter(abs(logFC) > 1.2 , P.Value < 0.05)

### save data

save(GSE57065_DEG, GSE57065_DEG_p,

file = ".\\GSE57065_DEG.Rda")

### 5.12 Draw volcano plot

## plot_df

plot_df <- GSE57065_DEG

# Define volcano plot thresholds

plot_df$threshold = factor(ifelse(plot_df$P.Value < 0.05 & abs(plot_df$logFC) > 1.2,

ifelse(plot_df$logFC > 1.2 ,'Up','Down'),'NS'),

levels=c('Up','Down','NS'))

# Start plotting

library(ggplot2)

pdf('./figures/Volcano_GSE57065.pdf', width = 8, height = 8)

ggplot(plot_df, aes(y = logFC, x = -log10(P.Value), color = threshold)) +

geom_point(alpha = 0.8) +# point size

scale_color_manual(values = c("#DC143C", "#143cdc", "#808080")) +# point color

xlab('-log10 (P-value)') +

ylab('log2 (FoldChange)') +

ggtitle("Volcano Plot") +

geom_hline(yintercept = c(-1.2, 1.2), lty = 3, col = "black", lwd = 0.5) +

geom_vline(xintercept = -log10(0.05), lty = 3, col = "black", lwd = 0.5) +

theme_bw() +

theme(legend.title = element_blank(),

legend.position = "none",

plot.title = element_text(hjust = 0.5))

dev.off()

p5 <- ggplot(plot_df, aes(y = logFC, x = -log10(P.Value), color = threshold)) +

geom_point(alpha = 0.8) +# point size

scale_color_manual(values = c("#DC143C", "#143cdc", "#808080")) +# point color

xlab('-log10 (P-value)') +

ylab('log2 (FoldChange)') +

ggtitle("Volcano Plot") +

geom_hline(yintercept = c(-1.2, 1.2), lty = 3, col = "black", lwd = 0.5) +

geom_vline(xintercept = -log10(0.05), lty = 3, col = "black", lwd = 0.5) +

theme_bw() +

theme(legend.title = element_blank(),

legend.position = "none",

plot.title = element_text(hjust = 0.5))

p5

dev.off()

save(p5, file = "./p5.Rda")

#### 6 GSE95233 ----

### 6.1 load packages

# rm(list = ls())

library(GEOquery)

library(readxl)

library(tidyverse)

### 2 Load data

setwd("F:\\HelixCourses\\GEOdatasets_spsis1\\DEG_SPSIS")

getwd()

GSE95233_sm<- getGEO(filename = "GSE95233_series_matrix.txt.gz",

destdir = ".",

getGPL = F )

GSE95233_expr <- exprs(GSE95233_sm)

head(GSE95233_expr)[, 1:6]

GSE95233_pd <- pData(GSE95233_sm)

### Create file

# dir.create("GSE95233")

if(!dir.exists("GSE95233"))dir.create("GSE95233")

setwd("F:\\HelixCourses\\GEOdatasets_spsis1\\DEG_SPSIS\\GSE95233")

getwd()

### Save data

save(GSE95233_sm, GSE95233_expr, GSE95233_pd,

file = ".\\GSE95233.Rda")

# load(".\\GSE95233_data.Rda")

### Organize target data

GSE95233_pd_1 <- GSE95233_pd # "gsub" means replace characters # "F:/Dr. Chen/Dr. Chen wants to lie down/Xena.R" Code: P1455

GSE95233_pd_1$title <- gsub("-","_",GSE95233_pd_1$title)

GSE95233_targets <- GSE95233_pd_1 %>% dplyr::select(2, 1, 8) %>%

# dplyr::select(1, 2) %>%

separate(title, into = paste0("x", 1:4), sep = "_", remove = F) %>%

filter(!(x4 %in% c("D02", "D03"))) %>% # "F:/HelixCourses/R Language from Scratch to Mastery_7 days/Day 3/Section 3 Live Broadcast" Code: P264

mutate(sample_id = geo_accession,# add several columns

#file_name = paste0(sample_id, ".CEL.gz"),

#sample_name = title,

sample_name = str_c(x1, x2, x3, sep = "_"),# Join multiple strings into a single string.

patient_id = x3 ,

# patient_id = str_match(GSE54514_targets_2$x4, "\\d+")[, 1],

# patient_id_1 = str_match(GSE54514_targets_2$x4, "\\d+")[, 1],

# strings match the first character

# and see the computer 360 browser collection folder (matching characters)

# and see (13_Reading Affymetrix chip data.R) Code: P1455

#str_sub----Extract and replace substrings from a character vector. Extract the substring from the last character

#GSE_num = "GSE69063",

# Below this line of code is seen in: "F:/Dr. Chen/Dr. Chen wants to lie down/GSE84402" Code: P240~P241

# group_list <- ifelse(str_detect(pdata$source_name_ch1, "hepatocellular carcinoma"), "tumor", "normal")

group = ifelse(str_sub(source_name_ch1, 1, 1) == "P","Sepsis","Control")) %>%

dplyr::select(sample_id:group) #%>%

# dplyr::select(2,3,4,1)

GSE95233_expr <- GSE95233_expr[ , GSE95233_targets$sample_id] %>% as.data.frame()

### View Data

range(GSE95233_expr)

# exp <- log2(GSE57065_expr+1)

# range(GSE57065_expr)

### Evaluate data

if(!dir.exists("figures")) dir.create("figures")

# boxplot(GSE29450_rma, las = 2, col = rep(c("blue", "red"), each = 10))

# boxplot(GSE95233_expr, las=2, outline=FALSE,

# col = rep(c("blue", "red"), c(22,51)),

# main = "GSE95233_expr")

boxplot(GSE95233_expr, las=2, outline=FALSE,

# col = rep(c("blue", "red"), c(22,51)),

main = "GSE95233_expr")

dev.off()

source("F:\\HelixCourses\\GEOdatasets_spsis1\\DEG_SPSIS\\Custom_Functions.R")

targets <- GSE95233_targets

pdf('./figures/PCA_GSE95233.pdf')

PCA_new(exprs(GSE95233_rma),

nrow(GSE95233_rma),

group = GSE95233_targets$group)

dev.off()

# View the original data processing method

GSE95233_sm@phenoData@data$data_processing[1]

# GSE95233_pd$data_processing[1] # This code is equivalent to the above code

### 6.5 Remove batch effect

## PCA analysis shows that the normal group and the sepsis group have batch effects,

## so the original data is read to remove batch effects

## Use oligo package to read CEL files

library(oligo)

cel_files <- list.celfiles("F:/HelixCourses/GEOdatasets_spsis1/DEG_SPSIS/GSE95233/GSE95233_RAW/",

full.name = T,

# full.name = F,

listGzipped = T)

# # str_match returns multiple column matching results, the first column is the overall matching result,

# # the following columns are the matching results for each group.

# c <- str_match(cel_files,"GSM\\d+")[,1] # Extract the vector

# c1 <- str_match(cel_files,"GSM\\d+") # Extract the list

# c2 <- str_match(cel_files,"GSM\\d+")[,2]

# d <- str_match(GSE54514_targets_2$x4, "\\d+")[, 1]

# d <- str_match(GSE54514_targets_2$x4, "\\d+")[, 2]

cel_files_1 <- ifelse(str_match(cel_files,"GSM\\d+") %in% GSE95233_targets$sample_id,TRUE,FALSE)

cel_files <- cel_files[cel_files_1]

cel_files1 <- list.celfiles("F:/HelixCourses/GEOdatasets_spsis1/DEG_SPSIS/GSE95233/GSE95233_RAW/",

# full.name = T,

full.name = F,

listGzipped = T)

cel_files_11 <- ifelse(str_match(cel_files1,"GSM\\d+") %in% GSE95233_targets$sample_id,TRUE,FALSE)

cel_files1 <- cel_files1[cel_files_11]

identical(str_match(cel_files,"GSM\\d+")[,1],GSE95233_targets$sample_id)

identical(cel_files_1,cel_files_11)

GSE95233_targets_FileName <- GSE95233_targets %>% mutate(FileName = cel_files) %>%

relocate(FileName,everything())

## new cel

GSE95233_cel <- read.celfiles(filenames = GSE95233_targets_FileName$FileName,

phenoData = AnnotatedDataFrame(data = GSE95233_targets_FileName),

sampleNames = GSE95233_targets_FileName$sample_id)

# GSE95233_cel1 <- read.celfiles(cel_files) # This reading method only obtains the expression matrix, without target and sampleNames data

## Save raw data

save(GSE95233_cel, file = "./GSE95233_cel.Rdata")

## Lookup batch effect

# cel <- GSE95233_cel

# cel_pd <- pData(cel)

# cel@protocolData@data$dates

# cel_dates <- str_split(cel@protocolData@data$dates, pattern = "\\s+", simplify = T)[,1]

# \s matches any whitespace character, and is equivalent to [ \f\n\r\t\v]

class(cel_dates)

table(cel_dates) # Check batches

# Chip scanning date can be considered as an important batch effect factor

GSE95233_targets_batch <- GSE95233_targets

GSE95233_targets_batch$batch <- GSE95233_cel@protocolData@data$dates %>%

str_sub(1, 8)

table(GSE95233_targets_batch$batch)

## 6.5.1 Do not include grouping factors

library(sva)

mod <- model.matrix(~1, data= GSE95233_targets_batch)

batch <- GSE95233_targets_batch$batch

GSE95233_expr <- exprs(GSE95233_rma) %>% as.data.frame()

range(GSE95233_expr)

# GSE95233_expr_rma <- rownames_to_column(GSE95233_expr, "ID")

# GSE95233_expr_rma <- GSE95233_expr_rma[!duplicated(GSE95233_expr_rma$ID), ]

GSE95233_combat <- ComBat(dat = GSE95233_expr,

batch = batch,

mod = mod,

par.prior=TRUE, # Use parameter estimation, false for non-parametric estimation which is more time-consuming

prior.plots=FALSE)

## 6.5.2 Include grouping factors, tell the algorithm what grouping factors we are interested in

mod1 <- model.matrix(~ as.factor(group), data= GSE95233_targets_batch)

mod2 <- model.matrix(~1 + group, data= GSE95233_targets_batch)

GSE95233_combat1 <- ComBat(dat = GSE95233_expr,

batch = batch,

mod = mod1,

par.prior=TRUE,

prior.plots=FALSE)

affy::boxplot(GSE95233_expr, las = 2,

col = rep(c("blue", "red"), c(22,51)), outline = F)

affy::boxplot(GSE95233_combat, las = 2,

col = rep(c("blue", "red"), c(22,51)), outline = F)

affy::boxplot(GSE95233_combat1, las = 2,

col = rep(c("blue", "red"), c(22,51)), outline = F)

dev.off()

## plot

GSE95233_p0 <- PCA_new(GSE95233_expr,

ntop = 10000,

# ntop = nrow(GSE95233_expr_n),

show_name = F)

GSE95233_p0

GSE95233_p1 <- PCA_new(GSE95233_expr_n,

ntop = 10000,

group = GSE95233_targets_batch$group,

show_name = F) +

ggtitle("PCA before batch effect correction") +

theme(plot.title = element_text(hjust = 0.5))

GSE95233_p1

GSE95233_p2 <- PCA_new(GSE95233_combat,

ntop = 10000,

group = GSE95233_targets_batch$group,

show_name = T)+

ggtitle("PCA before batch effect correction") +

theme(plot.title = element_text(hjust = 0.5))

GSE95233_p2

GSE95233_p3 <- PCA_new(GSE95233_combat1,

ntop = 10000,

group = GSE95233_targets_batch$group,

show_name = T)+

ggtitle("PCA before batch effect correction") +

theme(plot.title = element_text(hjust = 0.5))

GSE95233_p3

library(cowplot)

GSE95233_p0123 <- cowplot::plot_grid(GSE95233_p0, GSE95233_p1, GSE95233_p2, GSE95233_p3, nrow = 2)

# pdf("batch/PCA_GSE95233_batch.pdf", height = 7, width = 15)

GSE95233_p0123

dev.off()

# After the above analysis, it was found that the data obtained by reading the original data and removing batch effects is the most reasonable,

# so this part of the data is used for subsequent analysis

### 6.10 Annotation ----

GSE95233_sm@annotation

## GPL570

library(hgu133plus2.db)

library(illuminaHumanv3.db)

ls("package:hgu133plus2.db")

p2s <- toTable(hgu133plus2SYMBOL) #%>%

# set_names("probe_id", "symbol")# rename

head(p2s)

## Custom annotation function

annotate_expr <- function(expr, p2s, fun = mean){# Define three parameters, the column names of p2s are fixed

library(dplyr)

library(tibble)

# expr

expr <- expr %>%

as.data.frame() %>%

tibble::rownames_to_column("probe_id")# Change the row name to a column called "probe_id"

# annotate

expr_annotated <- p2s %>%

dplyr::inner_join(expr, by = "probe_id") %>% # Take the intersection of expr and p2s

na.omit() %>% # Remove NAs

dplyr::select(-probe_id) %>% # Remove the "probe_id" column

stats::aggregate(. ~ symbol, data =., FUN = fun) %>% # For multiple probes corresponding to the same symbol, take the mean of these probes

tibble::column_to_rownames("symbol")# Change the "symbol" column to the row name

return(expr_annotated)# Return this expr_annotated, and then assign it to annotate_expr

}

## Annotation

GSE95233_expr_anno <- annotate_expr(GSE95233_combat1, p2s)

head(GSE95233_expr_anno)[, 1:3]

## save data

GSE95233_expr_n <- GSE95233_combat1

GSE95233_anno <- GSE95233_expr_anno

GSE95233_targets <- GSE95233_targets_batch

save(GSE95233_expr_n,GSE95233_targets, GSE95233_anno,

file = ".\\GSE95233_processed.Rda")

### 6.11 Differential analysis DEGs ----

library(limma)

# For design

design <- model.matrix(~ group, data = GSE95233_targets_batch)

colnames(design)

colnames(design) <- c("Control", "SepsisvsControl")

head(design)

# arrayWeights

expr <- GSE95233_anno

aw <- arrayWeights(expr, design = design)

aw

barplot(aw)

# Use limma for differential analysis

fit <- lmFit(expr, design, weights = aw)

fit <- contrasts.fit(fit, contrast.matrix)

fit <- eBayes(fit)

## DEG results

results <- summary(decideTests(fit, lfc = 1.2))

results

# number = Inf: all differential analysis results; SepsisvsControl: represents the type to be compared

res_DEG <- topTable(fit, coef = "SepsisvsControl", number = Inf)

# res_DEG_sig <- topTable(fit, coef = "SepsisvsControl", n = Inf, p.value = 0.05, lfc = 1)# Here p is the corrected p

res_DEG_sig <- topTable(fit, coef = "SepsisvsControl", n = Inf, p.value = 0.05, lfc = 1.2)

GSE95233_DEG1 <- topTable(fit, coef = 2, n = Inf)

identical(res_DEG, GSE95233_DEG1)

GSE95233_DEG <- topTable(fit, coef = 2, n = Inf) %>% # coef = 1 when there is no contrast.matrix()

rownames_to_column(var = "symbol")

# Filter for |logFC|>1.2 and p<0.05 results

GSE95233_DEG_p <- GSE95233_DEG %>%

dplyr::filter(abs(logFC) > 1.2 , P.Value < 0.05)

### save data

save(GSE95233_DEG, GSE95233_DEG_p,

file = ".\\GSE95233_DEG.Rda")

### 6.12 Draw volcano plot

## plot_df

plot_df <- GSE95233_DEG

# Define volcano plot thresholds

plot_df$threshold = factor(ifelse(plot_df$P.Value < 0.05 & abs(plot_df$logFC) > 1.2,

ifelse(plot_df$logFC > 1.2 ,'Up','Down'),'NS'),

levels=c('Up','Down','NS'))

# Start plotting

library(ggplot2)

pdf('./figures/Volcano_GSE95233.pdf', width = 8, height = 8)

ggplot(plot_df, aes(y = logFC, x = -log10(P.Value), color = threshold)) +

geom_point(alpha = 0.8) +# point size

scale_color_manual(values = c("#DC143C", "#143cdc", "#808080")) +# point color

xlab('-log10 (P-value)') +

ylab('log2 (FoldChange)') +

ggtitle("Volcano Plot") +

geom_hline(yintercept = c(-1.2, 1.2), lty = 3, col = "black", lwd = 0.5) +

geom_vline(xintercept = -log10(0.05), lty = 3, col = "black", lwd = 0.5) +

theme_bw() +

theme(legend.title = element_blank(),

legend.position = "none",

plot.title = element_text(hjust = 0.5))

dev.off()

p6 <- ggplot(plot_df, aes(y = logFC, x = -log10(P.Value), color = threshold)) +

geom_point(alpha = 0.8) +# point size

scale_color_manual(values = c("#DC143C", "#143cdc", "#808080")) +# point color

xlab('-log10 (P-value)') +

ylab('log2 (FoldChange)') +

ggtitle("Volcano Plot") +

geom_hline(yintercept = c(-1.2, 1.2), lty = 3, col = "black", lwd = 0.5) +

geom_vline(xintercept = -log10(0.05), lty = 3, col = "black", lwd = 0.5) +

theme_bw() +

theme(legend.title = element_blank(),

legend.position = "none",

plot.title = element_text(hjust = 0.5))

p6

save(p6,file = "p6.Rda")

dev.off()

# Merge volcano plots----

rm(list = ls())

setwd("F:/HelixCourses/GEOdatasets_spsis1/DEG_SPSIS")

load("./GSE137340/P1.Rda")

load("./GSE69063/P2.Rda")

load("./GSE69528/P3.Rda")

load("./GSE54514/P4.Rda")

load("./GSE57065/P5.Rda")

load("./GSE95233/P6.Rda")

library(cowplot)

# dev.off()

# Coordinate axis horizontal

p <- cowplot::plot_grid(p1, p2, p3, p4, p5, p6, nrow = 2)# grid plot

p

dev.off()

if(!dir.exists("figures"))dir.create("figures")

# tiff("batch/gene_wise.tiff", height = 10, width = 10, unit = "in",res = 300)

pdf('F:/HelixCourses/GEOdatasets_spsis1/DEG_SPSIS/figures/Volcano_batch_cross.pdf',width = 10,height = 10)

cowplot::plot_grid(p1, p2, p3, p4, p5, p6, nrow = 2)

dev.off()

# Coordinate axis vertical

library(ggplot2)

p1_1 <- p1 + coord_fixed(ratio = 1)+coord_flip()+

theme(panel.grid.major =element_blank(), # Remove major grid lines

panel.grid.minor = element_blank(),# Remove minor grid lines

# panel.border = element_blank(),# Remove border

panel.background = element_blank())# Remove background

pdf('./figures/p1_1.pdf')

p1_1

dev.off()

p2_1 <- p2 + coord_fixed(ratio = 1)+coord_flip()+

theme(panel.grid.major =element_blank(), # Remove major grid lines

panel.grid.minor = element_blank(),# Remove minor grid lines

# panel.border = element_blank(),# Remove border

panel.background = element_blank())# Remove background

pdf('./figures/p2_1.pdf')

p2_1

dev.off()

p3_1 <- p3 + coord_fixed(ratio = 1)+coord_flip()+

theme(panel.grid.major =element_blank(), # Remove major grid lines

panel.grid.minor = element_blank(),# Remove minor grid lines

# panel.border = element_blank(),# Remove border

panel.background = element_blank())# Remove background

pdf('./figures/p3_1.pdf')

p3_1

dev.off()

p4_1 <- p4 + coord_fixed(ratio = 1)+coord_flip()+

theme(panel.grid.major =element_blank(), # Remove major grid lines

panel.grid.minor = element_blank(),# Remove minor grid lines

# panel.border = element_blank(),# Remove border

panel.background = element_blank())# Remove background

pdf('./figures/p4_1.pdf')

p4_1

dev.off()

p5_1 <- p5 + coord_fixed(ratio = 1)+coord_flip()+

theme(panel.grid.major =element_blank(), # Remove major grid lines

panel.grid.minor = element_blank(),# Remove minor grid lines

# panel.border = element_blank(),# Remove border

panel.background = element_blank())# Remove background

pdf('./figures/p5_1.pdf')

p5_1

dev.off()

p6_1 <- p6 + coord_fixed(ratio = 1)+coord_flip()+

theme(panel.grid.major =element_blank(), # Remove major grid lines

panel.grid.minor = element_blank(),# Remove minor grid lines

# panel.border = element_blank(),# Remove border

panel.background = element_blank())# Remove background

pdf('./figures/p6_1.pdf')

p6_1

dev.off()

p_1 <- cowplot::plot_grid(p1_1, p2_1, p3_1, p4_1, p5_1, p6_1, nrow = 2)# grid plot

p_1

dev.off()

if(!dir.exists("figures"))dir.create("figures")

# tiff("batch/gene_wise.tiff", height = 10, width = 10, unit = "in",res = 300)

pdf('F:/HelixCourses/GEOdatasets_spsis1/DEG_SPSIS/figures/Volcano_batch_vertical.pdf')

# ,width = 15,height = 10)

cowplot::plot_grid(p1_1, p2_1, p3_1, p4_1, p5_1, p6_1, nrow = 2)

dev.off()

# Same project.R Code: 1863

# # library(cowplot)

# # plot_grid(plotlist=pp, nrow = 2)

setwd("F:\\HelixCourses\\GEOdatasets_spsis1\\DEG_SPSIS")

getwd()

###

save(p1, p2, p3, p4, p5, p6, file = ".\\P_batch.Rdata")

save(p1, p2, p3, p4, p5, p6, file = ".\\P_batch.Rda")

#### 7 Results ----

#### 7.1 RRA ----

rm(list=ls())

library(RobustRankAggreg)

# The principle is very simple: if a gene is ranked high and appears frequently, it is more likely to be a significant differential gene

### load data

setwd("F:\\HelixCourses\\GEOdatasets_spsis1\\DEG_SPSIS")

load("./GSE137340/GSE137340_DEG.Rda")

load(".//GSE69063/GSE69063_DEG.Rda")

load("./GSE69528/GSE69528_DEG.Rda")

load("./GSE54514/GSE54514_DEG.Rda")

load("./GSE57065/GSE57065_DEG.Rda")

load("./GSE95233/GSE95233_DEG.Rda")

# c(GSE137340_DEG, GSE69063_DEG, GSE69528_DEG, GSE54514_DEG, GSE57065_DEG, GSE95233_DEG)

# c(GSE137340_DEG_p, GSE69063_DEG_p, GSE69528_DEG_p, GSE54514_DEG_p, GSE57065_DEG_p, GSE95233_DEG_p)

GSEs <- c("GSE137340", "GSE69063", "GSE69528", "GSE54514", "GSE57065", "GSE95233")

# Filter for up-regulated and down-regulated genes in each dataset

glist_up <- list()# Create two empty lists

glist_down <- list()

library(tidyverse)

### 7.1.1 RRA Conventional ----

# for(i in 1:6){ #get Return the value of a named object

# glist_up[[i]] <- get(paste0(GSEs[i],"_DEG_p")) %>% filter(logFC > 0) %>% .[[1]]

# glist_down[[i]] <- get(paste0(GSEs[i],"_DEG_p")) %>% filter(logFC < 0) %>% .[[1]]

# }

#

# # Perform RRA analysis: # aggregateRanks is the most crucial function

# res_RRA_up <- aggregateRanks(glist_up) # Rank genes and sort them, Score is the p-value after RRA aggregation

# res_RRA_down <- aggregateRanks(glist_down)

#

# # Combine up-regulated and down-regulated results

# all_signi_result <- bind_rows(res_RRA_up %>% mutate(direction = "up"),

# res_RRA_down %>% mutate(direction = "down")) %>%

# filter(Score < 0.05) %>% # Score is the p-value after RRA aggregation

# rownames_to_column(var = "SYMBOL") %>%

# write_tsv("./RRP/all_signi_result.tsv")

#

# # Extract the top 10 up-regulated and down-regulated results separately

# top10_up <- res_RRA_up %>% # Extract the top 10

# mutate(across(.fn = as.character)) %>% # across converts each row to as.character

# dplyr::slice(1:10) %>% # Take the first 10 rows

# .[[1]] # Then take the first column

#

# top10_down <- res_RRA_down %>%

# mutate(across(.fn = as.character)) %>%

# dplyr::slice(1:10) %>%

# .[[1]]

#

# top20 <- c(top10_up, top10_down)

#

# ### Construct heatmap data

# # Filter for the top genes' symbols and logFC in each GSE

# heatmap_list <- list()

# for(i in 1:6) {

# heatmap_list[[i]] <- get(paste0(GSEs[i], "_DEG")) %>%

# dplyr::select(1, 2) %>% # Select the first two columns: take symbol and logFC

# dplyr::filter(symbol %in% top20) %>% # Filter for symbols in top20

# mutate(GSE = GSEs[i])# Add a GSE column

# }

#

# # Combine the filtered results from all GSEs into a single plotting data

# heatmap_df <- bind_rows(heatmap_list) %>% # This is long data

# # Since not every GSE contains data for the top20 genes, use bind_rows to combine data, filling blanks with NA

# pivot_wider(names_from = GSE, values_from = logFC) %>% # Convert long data to wide data

# mutate(symbol = factor(symbol, levels = top20)) %>% # Add a column

# arrange(symbol) %>% # Sort by symbol

# column_to_rownames(var = "symbol")# Change the symbol column to row name

#

# # Figure

# # install.packages('pheatmap')

# library(pheatmap)

# pdf("./RRP/heatmap_Figure.pdf", height = 8, width = 8)

# pheatmap::pheatmap(mat = heatmap_df,

# color = colorRampPalette(c("navy", "white", "firebrick3"))(100),

# cluster_rows = F,

# cluster_cols = F,

# display_numbers = T)

# dev.off()

#

# save(res_RRA_up, res_RRA_down, heatmap_list, heatmap_df, file = "./RRP/RRP.Rda")

### 7.1.2 RRA This project ----

## Filter for adj. p-value < 0.05 results

GSE137340_DEG_S <- GSE137340_DEG %>% dplyr::filter(adj.P.Val < 0.05)

GSE69063_DEG_S <- GSE69063_DEG %>% dplyr::filter(adj.P.Val < 0.05)

GSE69528_DEG_S <- GSE69528_DEG %>% dplyr::filter(adj.P.Val < 0.05)

GSE54514_DEG_S <- GSE54514_DEG %>% dplyr::filter(adj.P.Val < 0.05)

GSE57065_DEG_S <- GSE57065_DEG %>% dplyr::filter(adj.P.Val < 0.05)

GSE95233_DEG_S <- GSE95233_DEG %>% dplyr::filter(adj.P.Val < 0.05)

## Filter for |logFC|>1.2 and adj. p-value<0.05 results

GSE137340_DEG_P_F <- GSE137340_DEG %>% dplyr::filter(abs(logFC) > 1.2 , adj.P.Val < 0.05)

GSE69063_DEG_P_F <- GSE69063_DEG %>% dplyr::filter(abs(logFC) > 1.2 , adj.P.Val < 0.05)

GSE69528_DEG_P_F <- GSE69528_DEG %>% dplyr::filter(abs(logFC) > 1.2 , adj.P.Val < 0.05)

GSE54514_DEG_P_F <- GSE54514_DEG %>% dplyr::filter(abs(logFC) > 1.2 , adj.P.Val < 0.05)

GSE57065_DEG_P_F <- GSE57065_DEG %>% dplyr::filter(abs(logFC) > 1.2 , adj.P.Val < 0.05)

GSE95233_DEG_P_F <- GSE95233_DEG %>% dplyr::filter(abs(logFC) > 1.2 , adj.P.Val < 0.05)

# Filter for up-regulated and down-regulated genes

glist_up <- list()# Create two empty lists

glist_down <- list()

## Use RRA package to extract genes with adj. p-value < 0.05

GSEs

GSEs[6]

for(i in 1:6){ #get Return the value of a named object

glist_up[[i]] <- get(paste0(GSEs[i],"_DEG_S")) %>% filter(logFC > 0) %>% .[[1]]

glist_down[[i]] <- get(paste0(GSEs[i],"_DEG_S")) %>% filter(logFC < 0) %>% .[[1]]

}

# Perform RRA analysis: # aggregateRanks is the most crucial function

library(RobustRankAggreg)

res_RRA_up_P <- aggregateRanks(glist_up) # Rank genes and sort them, Score is the p-value after RRA aggregation

res_RRA_down_P <- aggregateRanks(glist_down)

# Combine up-regulated and down-regulated results

all_signi_result_P <- bind_rows(res_RRA_up_P %>% mutate(direction = "up"),

res_RRA_down_P %>% mutate(direction = "down")) %>%

filter(Score < 0.05) %>% # Score is the p-value after RRA aggregation

rownames_to_column(var = "SYMBOL") %>%

write_tsv("./RRP/all_signi_result_P.tsv")

# Use RRA package to extract genes with |logFC|>1.2 and adj. p-value<0.05

for(i in 1:6){ #get Return the value of a named object

glist_up[[i]] <- get(paste0(GSEs[i],"_DEG_P_F")) %>% filter(logFC > 0) %>% .[[1]]

glist_down[[i]] <- get(paste0(GSEs[i],"_DEG_P_F")) %>% filter(logFC < 0) %>% .[[1]]

}

# Perform RRA analysis: # aggregateRanks is the most crucial function

res_RRA_up_P_F <- aggregateRanks(glist_up) # Rank genes and sort them, Score is the p-value after RRA aggregation

res_RRA_down_P_F <- aggregateRanks(glist_down)

# Combine up-regulated and down-regulated results

all_signi_result_P_F <- bind_rows(res_RRA_up_P_F %>% mutate(direction = "up"),

res_RRA_down_P_F %>% mutate(direction = "down")) %>%

filter(Score < 0.05) %>% # Score is the p-value after RRA aggregation

rownames_to_column(var = "SYMBOL") %>%

write_tsv("./RRP/all_signi_result_P_F.tsv")

# Extract the top 10 up-regulated and down-regulated results separately

top10_up_P_F <- res_RRA_up_P_F %>% # Extract the top 10

mutate(across(.fn = as.character)) %>% # across converts each row to as.character

dplyr::slice(1:10) %>% # Take the first 10 rows

.[[1]] # Then take the first column

top10_down_P_F <- res_RRA_down_P_F %>%

mutate(across(.fn = as.character)) %>%

dplyr::slice(1:10) %>%

.[[1]]

top20_P_F <- c(top10_up_P_F, top10_down_P_F)

### Construct heatmap data

# Filter for the top genes' symbols and logFC in each GSE

heatmap_list_P_F <- list()

for(i in 1:6) {

heatmap_list_P_F[[i]] <- get(paste0(GSEs[i], "_DEG_P_F")) %>%

dplyr::select(1, 2) %>% # Select the first two columns: take symbol and logFC

dplyr::filter(symbol %in% top20_P_F) %>% # Filter for symbols in top20

mutate(GSE = GSEs[i])# Add a GSE column

}

# Combine the filtered results from all GSEs into a single plotting data

heatmap_df_P_F <- bind_rows(heatmap_list_P_F) %>% # This is long data

# Since not every GSE contains data for the top20 genes, use bind_rows to combine data, filling blanks with NA

pivot_wider(names_from = GSE, values_from = logFC) %>% # Convert long data to wide data

# b <- gather(a,key=CIBERSORT,value = Proportion,-c(group,sample)) # Convert wide data to long data

mutate(symbol = factor(symbol, levels = top20_P_F)) %>% # Add a column

arrange(symbol) %>% # Sort by symbol

column_to_rownames(var = "symbol")# Change the symbol column to row name

# Figure

# install.packages('pheatmap')

library(pheatmap)

pdf("./RRP/heatmap_Figure_P_F.pdf", height = 8, width = 8)

# pheatmap::pheatmap(mat = heatmap_df_P_F,

# color = colorRampPalette(c("navy", "white", "firebrick3"))(100),

# cluster_rows = F,

# cluster_cols = F,

# display_numbers = T)

# dev.off()

pheatmap::pheatmap(mat = heatmap_df_P_F,

color = colorRampPalette(c("ForestGreen", "white", "firebrick3"))(100),

cluster_rows = F,

cluster_cols = F,

display_numbers = T)

dev.off()

save(res_RRA_up_P_F, res_RRA_down_P_F, heatmap_list_P_F, heatmap_df_P_F, file = "./RRP/RRP_P_F.Rda")

### 7.1.3 RRA: GSEA analysis----

## Load required R packages

rm(list=ls())

library(clusterProfiler)

library(org.Hs.eg.db)

## Load data

input <- read.csv("F:\\HelixCourses\\GEOdatasets_spsis1\\DEG_SPSIS\\RRP\\GSEA_data.csv",sep=",",header=T,check.names=F)

head(input)

# geneList = input[,1]

# class(geneList)

## Sort the geneList by Score1 value

# 1: Extract the Score1 values and store them in a vector

geneList = input[,4]

# 2: Name the geneList with the gene symbols

names(geneList) = as.character(input[,1])

class(geneList)

head(geneList)

## The gene names: ENSEMBLE

# MCEMP1 S100A12 UPP1 HP IRAK3 ARG1

## The corresponding logFC values of the genes

# 15.54516 15.25414 14.32422 14.19723 13.59176 13.10018

## Sort geneList by logFC values in descending order

geneList = sort(geneList, decreasing = TRUE)

class(geneList)

head(geneList)

# F:/HelixCourses/Full Code Breakthrough in Bioinformatics Data Mining/Part 2 of the Bioinformatics System Course/Level 2/L64_Codes/6-2-GSEA_GSVA_Analysis/Step02-GSEA_Plot.R Code: P15

# fpkm_sd_sorted = order(fpkm_sd, decreasing = T)

setwd("./RRP")

##

# gmt file folder name

msigdb_GMTs <- "msigdb_v2022.1.Hs_GMTs"

# The corresponding gene name for the input gene name, ENTREZID corresponds to Entrez Gene ID

# c2.cp.reactome.v2022.1.Hs.symbols.gmt corresponds to Gene Symbol name

# Specify the GMT file used for GSEA enrichment analysis

msigdb <- "c2.cp.reactome.v2022.1.Hs.symbols.gmt"

# Read the GMT file

all_msigdb <- read.gmt(file.path(msigdb_GMTs,msigdb))

gsegmt <- GSEA(geneList, TERM2GENE=all_msigdb, verbose=FALSE)#TERM2GENE, the relationship between TERM and gene name, is generally a two-column data frame format

head(gsegmt)

# Save the Reactome pathway GSEA analysis results to a file

write.table(gsegmt,file="./GSEA_reactome pathway_result.txt",sep="\t",

quote=F,row.names = F)

save(gsegmt, file = "./gsegmt.Rda")

### 7.1.4 RRA: GSEA plot----

### Load required R packages

# See F:/HelixCourses/Full Code Breakthrough in Bioinformatics Data Mining/Part 2 of the Bioinformatics System Course/Level 2/L62_Codes/6-2-GSEA_GSVA_Analysis/Step02-GSEA_Plot.R

rm(list = ls())

# if (!requireNamespace("DOSE", quietly = TRUE)) {

# BiocManager::install("DOSE", ask = F, update = F)

# }

library(clusterProfiler)

library(ggplot2)

## Ridge plot

# Read the file:

setwd("./RRP")

# gsegmt <- read.table(".\\GSEA_reactome pathway_result.txt",sep="\t",header=T,check.names=F)

load("./gsegmt.Rda")

# load(file = "./gsegmt.Rda") # Equivalent to the above

if(!dir.exists("figures")) dir.create("figures")

# options("repos" = c(CRAN = "<url id="" type="url" status="" title="" wc="">https://mirrors.tuna.tsinghua.edu.cn/CRAN/</url> "))

# options("BioC_mirror" = "<url id="" type="url" status="" title="" wc="">http://mirrors.tuna.tsinghua.edu.cn/bioconductor/</url> ")

# if (!requireNamespace("ggridges", quietly = TRUE)) {

# BiocManager::install("ggridges", ask = F, update = F)

# }

pdf("./figures/ridgeplot.pdf", 20, 8)

ridgeplot(gsegmt, showCategory = 6) # showCategory indicates the number of categories to display

# ??ridgeplot : ??Add a function to query

dev.off()

library(ggupset)

library(enrichplot)

## GSEA plot

# KEGG_result_df <- as.data.frame(KEGG) # F:/Dr. Chen/Dr. Chen wants to lie down/xena.R Code: 1300

gsegmt_result_df <- as.data.frame(gsegmt) # Convert to data frame

write.table(gsegmt_result_df, file = "gsegmt_result_df.txt",sep="\t", quote=F, col.names=T,row.names = T)

# pp6 <- lapply(1:6, function(i) {

# anno <- gsegmt[i, c("NES", "pvalue", "p.adjust")]

# lab <- paste0(names(anno), "=", round(anno, 3), collapse="\n")

#

# gseaplot2(gsegmt, i, gsegmt[i, 2],base_size = 5) + #xlab(NULL) +ylab(NULL) + # xlab(NULL),ylab(NULL): Hide axis labels

# # base_size = 5: Indicates font size

# annotate("text", x=1000, y=0, gsegmt[i, "enrichmentScore"] *.9, size=2, label = lab, hjust=0, vjust=0)

# annotate Inside the function, "text" is used as the type of annotation, and then x and y specify the position of the text. The content of the text is specified by the label parameter. See “<url id="" type="url" status="" title="" wc="">https://blog.csdn.net/g_r_c/article/details/19673625</url> ”

# # box(): Form a complete border (2023.2.8 added)

# # hjust =0 indicates left alignment, hjust =1 indicates right alignment, hjust =0.5 indicates center alignment

# }) # hjust =0 indicates left alignment, hjust =1 indicates right alignment, hjust =0.5 indicates center alignment

#

# # # pp and pp1 are both empty, the reason is that gseaplot2 draws three plots, and annotate() does not know which plot to add annotations to

# #

# pp2 <- lapply(1:3, function(i) {

# anno <- gsegmt[i, c("NES", "pvalue", "p.adjust")]

# lab <- paste0(names(anno), "=", round(anno, 3), collapse="\n")

#

# gsearank(gsegmt, i, gsegmt[i, 2]) + xlab(NULL) +ylab(NULL) +

# annotate("text", 0, gsegmt[i, "enrichmentScore"] *.9, label = lab, hjust=0, vjust=0)

# })

# plot_grid(plotlist=pp2, nrow = 2)

# # pp2 can be plotted, the reason is that gsearank draws one plot, and annotate() does not have to worry about which plot to add annotations to

# #

# # # The following code is to break down the steps above:

# anno <- gsegmt[1, c("NES", "pvalue", "p.adjust")]

# lab <- paste0(names(anno), "=", round(anno, 3), collapse="\n")

# # pvalue_table = lab

# # P4 <- gseaplot2(gsegmt, 1, gsegmt[1, 2])

# P5_0 <- gseaplot2(gsegmt, 1, gsegmt[1, 2],base_size = 6,subplots = 1)+

# annotate("text", x=500 ,y=0.2, size=5, label = lab)

# P5_0 <- P5_0 + gseaplot2(gsegmt, 1, gsegmt[1, 2],base_size = 6,subplots = 2)+plot_layout(nrow = 2)

# P5_1 <-gseaplot2(gsegmt, 1, gsegmt[1, 2],base_size = 6,subplots = 2)

# P5_2 <-gseaplot2(gsegmt, 1, gsegmt[1, 2],base_size = 6,subplots = 3)

# P5_0 + P5_1 + P5_2 + plot_layout(ncol = 1)

# plot_grid(P5_0+P5_1+P5_2, nrow = 3)

#

# P6 <- enrichplot::gseaplot2(gsegmt, 1, gsegmt[1, 2],base_size = 6,pvalue_table = TRUE)+

# annotate("text", x=2000 , y=6,size=5, label = lab, hjust=1, vjust=1) # Draw three plots

#

# # R how to install Git: Website: <url id="" type="url" status="" title="" wc="">https://www.jianshu.com/p/4d1388c82fc0</url>

# remotes::install_git('<url id="" type="url" status="" title="" wc="">https://gitee.com/swcyo/myenrichplot/</url> ') # Install the improved gseaplot2() plotting package via GitHub

# remotes::install_git('<url id="" type="url" status="" title="" wc="">https://gitee.com/swcyo/Rtoolbox</url> ') # Install the replotGSEA() plotting package via GitHub

# remotes::install_github("swcyo/myenrichplot")

# remotes::install_local("C:/Users/admin/Desktop/Rtoolbox-master_2.zip",upgrade = F,dependencies = T)

library(myenrichplot)

pp7 <- lapply(1:6, function(i) {

myenrichplot::gseaplot2(gsegmt, i, gsegmt[i, 2], pvalue_table = T) #xlab(NULL),ylab(NULL): Hide axis labels

# gseaplot2(gsegmt, i, gsegmt[i, 2],base_size = 5) + #xlab(NULL) +ylab(NULL) + # xlab(NULL),ylab(NULL): Hide axis labels

}) # hjust =0 indicates left alignment, hjust =1 indicates right alignment, hjust =0.5 indicates center alignment

pp8 <- lapply(1:6, function(i) {

enrichplot::gseaplot2(gsegmt, i, gsegmt[i, 2], pvalue_table = T)

# gseaplot2(gsegmt, i, gsegmt[i, 2],base_size = 5) + #xlab(NULL) +ylab(NULL) + # xlab(NULL),ylab(NULL): Hide axis labels

}) # hjust =0 indicates left alignment, hjust =1 indicates right alignment, hjust =0.5 indicates center alignment

# library(cowplot)

# pdf("./figures/GSEA_C2_pathway.pdf", 20, 8)

# plot_grid(plotlist=pp7, nrow = 2)

# dev.off()

# cowplot::plot_grid(p1, p2, p3, p4, p5, p6, nrow = 2) # Same project.R Code: 1576

pdf("./figures/GSEA_C2_pathway.pdf",14,4.5)

lapply(1:6, function(i) {

myenrichplot::gseaplot2(gsegmt, i, gsegmt[i, 2], pvalue_table = T) #xlab(NULL),ylab(NULL): Hide axis labels

})

dev.off()

# pdf("./figures/Boxplots.PDF", 15, 6) # See this project Code: 52~59

# for (i in 1:8) {

# boxplot(get(GSE_expr[i]),

# las=2,

# outline=FALSE,

# main = paste0("Boxplot for ", GSEs[i]))

# }

# dev.off()

### 8.2 cibersort_GSE69063 barplot and boxplot----

## 8.2.1 cibersort_GSE69063 barplot----

rm(list=ls())

library(e1071)

library(parallel)

library(preprocessCore)

library(tidyverse)

# if(!dir.exists("CIBERSORT")) dir.create("CIBERSORT")

setwd("F:/HelixCourses/GEOdatasets_spsis1/DEG_SPSIS/CIBERSORT")

source("./CIBERSORT.R")

if(!dir.exists("CIBERSORT_GSE69063"))dir.create("CIBERSORT_GSE69063")

# Assign the file name "LM22.txt" to sig_matrix, and the program will automatically read this file in the directory

sig_matrix <- "LM22.txt" # "LM22.txt" is the annotation file for 22 immune cells

load("F:/HelixCourses/GEOdatasets_spsis1/DEG_SPSIS/GSE69063/GSE69063_processed.Rda")

# write.table(GSE69063_anno, file = "GSE69063_anno.txt",sep="\t", quote=F, col.names=T,row.names = T)

mixture_file = 'GSE69063_anno.txt' # Expression data for the GSE137340 dataset (note to adjust the first row of the table)

# perm indicates the number of calculations; the more times, the more stable the result

res_cibersort <- CIBERSORT(sig_matrix,

mixture_file,

perm=100, # perm, the number of permutation tests, default is 0; significant p-values can be calculated when greater than 100

QN=TRUE) # QN, whether to normalize the input expression matrix

# Equivalent to calculating 100 times continuously to see if the result is random in 100 times

# Generate a normalization function normalize, create a function

normalize <- function(x){# (x - minimum of x) / (maximum of x - minimum of x)

return((x-min(x))/(max(x)-min(x)))}

# Normalize CIBERSORT scores using normalize

CIBERSORT_Score <- res_cibersort

CIBERSORT_Score_GSE69063 <- normalize(CIBERSORT_Score)

save(CIBERSORT_Score_GSE69063, CIBERSORT_Score,

file = "./CIBERSORT_GSE69063/CIBERSORT_Score_GSE69063.Rdata") # Save intermediate file

load("./CIBERSORT_GSE69063/CIBERSORT_Score_GSE69063.Rdata")

CIBERSORT_Score_GSE69063 <- CIBERSORT_Score_GSE69063[,1:22]

# colSums adds up the columns

ciber.res <- CIBERSORT_Score_GSE69063[,colSums(CIBERSORT_Score_GSE69063) > 0] # Remove cells with zero abundance

# Visualization (by Teacher Achim)

mycol <- ggplot2::alpha(rainbow(ncol(ciber.res)), 0.7) # Create a rainbow color palette (with 70% transparency)

# alpha: Add transparency, the range of alpha is from 0 to 1, 1 is the original color, the larger the number, the darker the color

par(bty="o", mgp = c(2.5,0.3,0), mar = c(2.1,4.1,2.1,10.1),tcl=-.25,las = 1,xpd = F)

# las: Can only be one of 0, 1, 2, 3, used to indicate the direction of the tick mark values. 0 means always parallel to the axis; 1 means always horizontal direction; 2 means always perpendicular to the axis; 3 means always vertical direction

# tcl: Can also be used to set the length of the tick marks, but the unit is different from tck. Its default value is -0.5

# bty: The parameter value is a string type, used to limit the type of the plot border. If the value of bty is "o" (default value: i.e., borders on all four sides), "l", "7", "c", "u", or "]" among any one, the corresponding border type is similar to the shape of the letter.

# If the value of bty is "n", it means no border.

# mar: A numerical vector of the form c(bottom, left, top, right) which gives the number of lines of margin to be specified on the four sides of the plot. The default is c(5, 4, 4, 2) + 0.1.

# xpd: Logical parameter or NA, FALSE means only display text within the plot region, TRUE means only display text within the figure region, NA means display text within the entire device region;

barplot(as.matrix(t(ciber.res)),

border = NA, # No border for the bars

names.arg = rep("",nrow(ciber.res)), # No sample names on the x-axis

yaxt = "n", # Do not draw the y-axis for now

ylab = "Relative percentage", # Modify the y-axis label

col = mycol) # Use the rainbow color palette

axis(side = 2, at = c(0,0.2,0.4,0.6,0.8,1), # Add percentages to the y-axis

labels = c("0%","20%","40%","60%","80%","100%"))

legend(par("usr")[2]-2, # Adjust the legend position according to the actual plot (here -20)

par("usr")[4],

legend = colnames(ciber.res),

xpd = T,

fill = mycol,

cex = 0.7,

border = NA,

y.intersp = 1,

x.intersp = 0.2,

bty = "n")

dev.off() # Close the plot

## 8.2.2 cibersort_GSE69063 boxplot----

library(tidyverse)

a <- CIBERSORT_Score_GSE69063 %>% as.data.frame()

b <- GSE69063_targets %>% dplyr::select(1,4) # Pipeline function (Ctrl + Shift + M)

identical(rownames(a),rownames(b))# Check if the row names of the two tables are in the same order

b$group <- as.factor(b$group) # Convert the group column in data frame b to a factor type

class(b$group)

# a$group <- b$group # Add the group column from table b to table a; note that both a and b must be data frames

# Remove cells with zero abundance

ciber.res <- CIBERSORT_Score_GSE69063[,colSums(CIBERSORT_Score_GSE69063) > 0] # Remove cells with zero abundance

a <- a [,colnames(ciber.res)] %>% rownames_to_column("sample")

a$group <- b$group # Add the group column from table b to table a; note that both a and b must be data frames

library(ggsci)

library(tidyr)

library(ggpubr)

# b <- b[rownames(a),]

# identical(rownames(a),rownames(b))

# # Take the intersection of row names

# comgene <- intersect(rownames(a),rownames(b))

# a <- a[comgene,]

# class(a)# Check data type

# class(comgene)

# b <- b[comgene,]

# identical(rownames(a),rownames(b))# Check if multiple elements are identical

b <- gather(a,key=CIBERSORT,value = Proportion,-c(group,sample)) # Convert wide data to long data (code: 2119)

# pivot_wider(names_from = GSE, values_from = logFC) # Convert long data to wide data (code: 1802)

pdf("./CIBERSORT_GSE69063/boxplot_GSE69063.pdf")

ggboxplot(b, x = "CIBERSORT", y = "Proportion",

fill = "group", palette = "lancet")+

stat_compare_means(aes(group = group),

method = "wilcox.test",

label = "p.signif",

symnum.args=list(cutpoints = c(0, 0.001, 0.01, 0.05, 1),

symbols = c("***", "**", "*", "ns")))+

theme(text = element_text(size=10), # Font size of all text in the plot

axis.text.x = element_text(angle=45, hjust=1))

# hjust =0 means left-aligned, hjust =1 means right-aligned, hjust =0.5 means centered

dev.off()

CB2 <- ggboxplot(b, x = "CIBERSORT", y = "Proportion",

fill = "group", palette = "lancet")+

stat_compare_means(aes(group = group),

method = "wilcox.test",

label = "p.signif",

symnum.args=list(cutpoints = c(0, 0.001, 0.01, 0.05, 1),

symbols = c("***", "**", "*", "ns")))+

theme(text = element_text(size=10),

axis.text.x = element_text(angle=45, hjust=1))

CB2

dev.off()

### 8.3 cibersort_GSE69528 barplot and boxplot----

## 8.3.1 cibersort_GSE69528 barplot----

rm(list=ls())

library(e1071)

library(parallel)

library(preprocessCore)

library(tidyverse)

# if(!dir.exists("CIBERSORT")) dir.create("CIBERSORT")

setwd("F:/HelixCourses/GEOdatasets_spsis1/DEG_SPSIS/CIBERSORT")

source("./CIBERSORT.R")

if(!dir.exists("CIBERSORT_GSE69528"))dir.create("CIBERSORT_GSE69528")

# Assign the file name "LM22.txt" to sig_matrix, and the program will automatically read this file in the directory

sig_matrix <- "LM22.txt" # "LM22.txt" is the annotation file for 22 immune cells

load("F:/HelixCourses/GEOdatasets_spsis1/DEG_SPSIS/GSE69528/GSE69528_processed.Rda")

# write.table(GSE69528_anno, file = "GSE69528_anno.txt",sep="\t", quote=F, col.names=T,row.names = T)

mixture_file = 'GSE69528_anno.txt' # Expression data for the GSE137340 dataset (note to adjust the first row of the table)

# perm indicates the number of calculations; the more times, the more stable the result

res_cibersort <- CIBERSORT(sig_matrix,

mixture_file,

perm=100, # perm, the number of permutation tests, default is 0; significant p-values can be calculated when greater than 100

QN=TRUE) # QN, whether to normalize the input expression matrix

# Equivalent to calculating 100 times continuously to see if the result is random in 100 times

# Generate a normalization function normalize, create a function

normalize <- function(x){# (x - minimum of x) / (maximum of x - minimum of x)

return((x-min(x))/(max(x)-min(x)))}

# Normalize CIBERSORT scores using normalize

CIBERSORT_Score <- res_cibersort

CIBERSORT_Score_GSE69528 <- normalize(CIBERSORT_Score)

save(CIBERSORT_Score_GSE69528, CIBERSORT_Score,

file = "./CIBERSORT_GSE69528/CIBERSORT_Score_GSE69528.Rdata") # Save intermediate file

load("./CIBERSORT_GSE69528/CIBERSORT_Score_GSE69528.Rdata")

CIBERSORT_Score_GSE69528<- CIBERSORT_Score_GSE69528[,1:22]

# colSums adds up the columns

ciber.res <- CIBERSORT_Score_GSE69528[,colSums(CIBERSORT_Score_GSE69528) > 0] # Remove cells with zero abundance

# Visualization (by Teacher Achim)

mycol <- ggplot2::alpha(rainbow(ncol(ciber.res)), 0.7) # Create a rainbow color palette (with 70% transparency)

# alpha: Add transparency, the range of alpha is from 0 to 1, 1 is the original color, the larger the number, the darker the color

par(bty="o", mgp = c(2.5,0.3,0), mar = c(2.1,4.1,2.1,10.1),tcl=-.25,las = 1,xpd = F)

# las: Can only be one of 0, 1, 2, 3, used to indicate the direction of the tick mark values. 0 means always parallel to the axis; 1 means always horizontal direction; 2 means always perpendicular to the axis; 3 means always vertical direction

# tcl: Can also be used to set the length of the tick marks, but the unit is different from tck. Its default value is -0.5

# bty: The parameter value is a string type, used to limit the type of the plot border. If the value of bty is "o" (default value: i.e., borders on all four sides), "l", "7", "c", "u", or "]" among any one, the corresponding border type is similar to the shape of the letter.

# If the value of bty is "n", it means no border.

# mar: A numerical vector of the form c(bottom, left, top, right) which gives the number of lines of margin to be specified on the four sides of the plot. The default is c(5, 4, 4, 2) + 0.1.

# xpd: Logical parameter or NA, FALSE means only display text within the plot region, TRUE means only display text within the figure region, NA means display text within the entire device region;

barplot(as.matrix(t(ciber.res)),

border = NA, # No border for the bars

names.arg = rep("",nrow(ciber.res)), # No sample names on the x-axis

yaxt = "n", # Do not draw the y-axis for now

ylab = "Relative percentage", # Modify the y-axis label

col = mycol) # Use the rainbow color palette

axis(side = 2, at = c(0,0.2,0.4,0.6,0.8,1), # Add percentages to the y-axis

labels = c("0%","20%","40%","60%","80%","100%"))

legend(par("usr")[2]-2, # Adjust the legend position according to the actual plot (here -20)

par("usr")[4],

legend = colnames(ciber.res),

xpd = T,

fill = mycol,

cex = 0.6,

border = NA,

y.intersp = 1,

x.intersp = 0.2,

bty = "n")

dev.off() # Close the plot

## 8.3.2 cibersort_GSE69528 boxplot----

library(tidyverse)

a <- CIBERSORT_Score_GSE69528 %>% as.data.frame()

b <- GSE69528_targets %>% dplyr::select(1,4) # Pipeline function (Ctrl + Shift + M)

identical(rownames(a),rownames(b))# Check if the row names of the two tables are in the same order

b$group <- as.factor(b$group) # Convert the group column in data frame b to a factor type

class(b$group)

# a$group <- b$group # Add the group column from table b to table a; note that both a and b must be data frames

# Remove cells with zero abundance

ciber.res <- CIBERSORT_Score_GSE69528[,colSums(CIBERSORT_Score_GSE69528) > 0] # Remove cells with zero abundance

a <- a [,colnames(ciber.res)] %>% rownames_to_column("sample")

a$group <- b$group # Add the group column from table b to table a; note that both a and b must be data frames

library(ggsci)

library(tidyr)

library(ggpubr)

# b <- b[rownames(a),]

# identical(rownames(a),rownames(b))

# # Take the intersection of row names

# comgene <- intersect(rownames(a),rownames(b))

# a <- a[comgene,]

# class(a)# Check data type

# class(comgene)

# b <- b[comgene,]

# identical(rownames(a),rownames(b))# Check if multiple elements are identical

b <- gather(a,key=CIBERSORT,value = Proportion,-c(group,sample)) # Convert wide data to long data (code: 2119)

# pivot_wider(names_from = GSE, values_from = logFC) # Convert long data to wide data (code: 1802)

pdf("./CIBERSORT_GSE69528/boxplot_GSE69528.pdf")

ggboxplot(b, x = "CIBERSORT", y = "Proportion",

fill = "group", palette = "lancet")+

stat_compare_means(aes(group = group),

method = "wilcox.test",

label = "p.signif",

symnum.args=list(cutpoints = c(0, 0.001, 0.01, 0.05, 1),

symbols = c("***", "**", "*", "ns")))+

theme(text = element_text(size=10), # Font size of all text in the plot

axis.text.x = element_text(angle=45, hjust=1))

# hjust =0 means left-aligned, hjust =1 means right-aligned, hjust =0.5 means centered

dev.off()

CB3 <- ggboxplot(b, x = "CIBERSORT", y = "Proportion",

fill = "group", palette = "lancet")+

stat_compare_means(aes(group = group),

method = "wilcox.test",

label = "p.signif",

symnum.args=list(cutpoints = c(0, 0.001, 0.01, 0.05, 1),

symbols = c("***", "**", "*", "ns")))+

theme(text = element_text(size=10),

axis.text.x = element_text(angle=45, hjust=1))

CB3

dev.off()

### 8.4 cibersort_GSE54514 barplot and boxplot----

## 8.4.1 cibersort_GSE54514 barplot----

rm(list=ls())

library(e1071)

library(parallel)

library(preprocessCore)

library(tidyverse)

# if(!dir.exists("CIBERSORT")) dir.create("CIBERSORT")

setwd("F:/HelixCourses/GEOdatasets_spsis1/DEG_SPSIS/CIBERSORT")

source("./CIBERSORT.R")

if(!dir.exists("CIBERSORT_GSE54514"))dir.create("CIBERSORT_GSE54514")

# Assign the file name "LM22.txt" to sig_matrix, and the program will automatically read this file in the directory

sig_matrix <- "LM22.txt" # "LM22.txt" is the annotation file for 22 immune cells

load("F:/HelixCourses/GEOdatasets_spsis1/DEG_SPSIS/GSE54514/GSE54514_processed.Rda")

# write.table(GSE54514_anno, file = "GSE54514_anno.txt",sep="\t", quote=F, col.names=T,row.names = T)

mixture_file = 'GSE54514_anno.txt' # Expression data for the GSE137340 dataset (note to adjust the first row of the table)

# perm indicates the number of calculations; the more times, the more stable the result

res_cibersort <- CIBERSORT(sig_matrix,

mixture_file,

perm=100, # perm, the number of permutation tests, default is 0; significant p-values can be calculated when greater than 100

QN=TRUE) # QN, whether to normalize the input expression matrix

# Equivalent to calculating 100 times continuously to see if the result is random in 100 times

# Generate a normalization function normalize, create a function

normalize <- function(x){# (x - minimum of x) / (maximum of x - minimum of x)

return((x-min(x))/(max(x)-min(x)))}

# Normalize CIBERSORT scores using normalize

CIBERSORT_Score <- res_cibersort

CIBERSORT_Score_GSE54514 <- normalize(CIBERSORT_Score)

save(CIBERSORT_Score_GSE54514, CIBERSORT_Score,

file = "./CIBERSORT_GSE54514/CIBERSORT_Score_GSE54514.Rdata") # Save intermediate file

load("./CIBERSORT_GSE54514/CIBERSORT_Score_GSE54514.Rdata")

CIBERSORT_Score_GSE54514<- CIBERSORT_Score_GSE54514[,1:22]

# colSums adds up the columns

ciber.res <- CIBERSORT_Score_GSE54514[,colSums(CIBERSORT_Score_GSE54514) > 0] # Remove cells with zero abundance

# Visualization (by Teacher Achim)

mycol <- ggplot2::alpha(rainbow(ncol(ciber.res)), 0.7) # Create a rainbow color palette (with 70% transparency)

# alpha: Add transparency, the range of alpha is from 0 to 1, 1 is the original color, the larger the number, the darker the color

par(bty="o", mgp = c(2.5,0.3,0), mar = c(2.1,4.1,2.1,10.1),tcl=-.25,las = 1,xpd = F)

# las: Can only be one of 0, 1, 2, 3, used to indicate the direction of the tick mark values. 0 means always parallel to the axis; 1 means always horizontal direction; 2 means always perpendicular to the axis; 3 means always vertical direction

# tcl: Can also be used to set the length of the tick marks, but the unit is different from tck. Its default value is -0.5

# bty: The parameter value is a string type, used to limit the type of the plot border. If the value of bty is "o" (default value: i.e., borders on all four sides), "l", "7", "c", "u", or "]" among any one, the corresponding border type is similar to the shape of the letter.

# If the value of bty is "n", it means no border.

# mar: A numerical vector of the form c(bottom, left, top, right) which gives the number of lines of margin to be specified on the four sides of the plot. The default is c(5, 4, 4, 2) + 0.1.

# xpd: Logical parameter or NA, FALSE means only display text within the plot region, TRUE means only display text within the figure region, NA means display text within the entire device region;

barplot(as.matrix(t(ciber.res)),

border = NA, # No border for the bars

names.arg = rep("",nrow(ciber.res)), # No sample names on the x-axis

yaxt = "n", # Do not draw the y-axis for now

ylab = "Relative percentage", # Modify the y-axis label

col = mycol) # Use the rainbow color palette

axis(side = 2, at = c(0,0.2,0.4,0.6,0.8,1), # Add percentages to the y-axis

labels = c("0%","20%","40%","60%","80%","100%"))

legend(par("usr")[2]-2, # Adjust the legend position according to the actual plot (here -20)

par("usr")[4],

legend = colnames(ciber.res),

xpd = T,

fill = mycol,

cex = 0.7,

border = NA,

y.intersp = 1,

x.intersp = 0.2,

bty = "n")

dev.off() # Close the plot

## 8.4.2 cibersort_GSE54514 boxplot----

library(tidyverse)

a <- CIBERSORT_Score_GSE54514 %>% as.data.frame()

b <- GSE54514_targets # Pipeline function (Ctrl + Shift + M)

identical(rownames(a),rownames(b))# Check if the row names of the two tables are in the same order

b$group <- as.factor(b$group) # Convert the group column in data frame b to a factor type

class(b$group)

# a$group <- b$group # Add the group column from table b to table a; note that both a and b must be data frames

# Remove cells with zero abundance

ciber.res <- CIBERSORT_Score_GSE54514[,colSums(CIBERSORT_Score_GSE54514) > 0] # Remove cells with zero abundance

a <- a [,colnames(ciber.res)] %>% rownames_to_column("sample")

a$group <- b$group # Add the group column from table b to table a; note that both a and b must be data frames

library(ggsci)

library(tidyr)

library(ggpubr)

# b <- b[rownames(a),]

# identical(rownames(a),rownames(b))

# # Take the intersection of row names

# comgene <- intersect(rownames(a),rownames(b))

# a <- a[comgene,]

# class(a)# Check data type

# class(comgene)

# b <- b[comgene,]

# identical(rownames(a),rownames(b))# Check if multiple elements are identical

b <- gather(a,key=CIBERSORT,value = Proportion,-c(group,sample)) # Convert wide data to long data (code: 2119)

# pivot_wider(names_from = GSE, values_from = logFC) # Convert long data to wide data (code: 1802)

pdf("./CIBERSORT_GSE54514/boxplot_GSE54514.pdf")

ggboxplot(b, x = "CIBERSORT", y = "Proportion",

fill = "group", palette = "lancet")+

stat_compare_means(aes(group = group),

method = "wilcox.test",

label = "p.signif",

symnum.args=list(cutpoints = c(0, 0.001, 0.01, 0.05, 1),

symbols = c("***", "**", "*", "ns")))+

theme(text = element_text(size=10), # Font size of all text in the plot

axis.text.x = element_text(angle=45, hjust=1))

# hjust =0 means left-aligned, hjust =1 means right-aligned, hjust =0.5 means centered

dev.off()

CB4 <- ggboxplot(b, x = "CIBERSORT", y = "Proportion",

fill = "group", palette = "lancet")+

stat_compare_means(aes(group = group),

method = "wilcox.test",

label = "p.signif",

symnum.args=list(cutpoints = c(0, 0.001, 0.01, 0.05, 1),

symbols = c("***", "**", "*", "ns")))+

theme(text = element_text(size=10),

axis.text.x = element_text(angle=45, hjust=1))

CB4

dev.off()

### 8.5 cibersort_GSE57065 barplot and boxplot----

## 8.5.1 cibersort_GSE57065 barplot----

rm(list=ls())

library(e1071)

library(parallel)

library(preprocessCore)

library(tidyverse)

# if(!dir.exists("CIBERSORT")) dir.create("CIBERSORT")

setwd("F:/HelixCourses/GEOdatasets_spsis1/DEG_SPSIS/CIBERSORT")

source("./CIBERSORT.R")

if(!dir.exists("CIBERSORT_GSE57065"))dir.create("CIBERSORT_GSE57065")

# Assign the file name "LM22.txt" to sig_matrix, and the program will automatically read this file in the directory

sig_matrix <- "LM22.txt" # "LM22.txt" is the annotation file for 22 immune cells

load("F:/HelixCourses/GEOdatasets_spsis1/DEG_SPSIS/GSE57065/GSE57065_processed.Rda")

# write.table(GSE57065_anno, file = "GSE57065_anno.txt",sep="\t", quote=F, col.names=T,row.names = T)

mixture_file = 'GSE57065_anno.txt' # Expression data for the GSE137340 dataset (note to adjust the first row of the table)

# perm indicates the number of calculations; the more times, the more stable the result

res_cibersort <- CIBERSORT(sig_matrix,

mixture_file,

perm=100, # perm, the number of permutation tests, default is 0; significant p-values can be calculated when greater than 100

QN=TRUE) # QN, whether to normalize the input expression matrix

# Equivalent to calculating 100 times continuously to see if the result is random in 100 times

# Generate a normalization function normalize, create a function

normalize <- function(x){# (x - minimum of x) / (maximum of x - minimum of x)

return((x-min(x))/(max(x)-min(x)))}

# Normalize CIBERSORT scores using normalize

CIBERSORT_Score <- res_cibersort

CIBERSORT_Score_GSE57065 <- normalize(CIBERSORT_Score)

save(CIBERSORT_Score_GSE57065, CIBERSORT_Score,

file = "./CIBERSORT_GSE57065/CIBERSORT_Score_GSE57065.Rdata") # Save intermediate file

load("./CIBERSORT_GSE57065/CIBERSORT_Score_GSE57065.Rdata")

CIBERSORT_Score_GSE57065<- CIBERSORT_Score_GSE57065[,1:22]

# colSums adds up the columns

ciber.res <- CIBERSORT_Score_GSE57065[,colSums(CIBERSORT_Score_GSE57065) > 0] # Remove cells with zero abundance

# Visualization (by Teacher Achim)

mycol <- ggplot2::alpha(rainbow(ncol(ciber.res)), 0.7) # Create a rainbow color palette (with 70% transparency)

# alpha: Add transparency, the range of alpha is from 0 to 1, 1 is the original color, the larger the number, the darker the color

par(bty="o", mgp = c(2.5,0.3,0), mar = c(2.1,4.1,2.1,10.1),tcl=-.25,las = 1,xpd = F)

# las: Can only be one of 0, 1, 2, 3, used to indicate the direction of the tick mark values. 0 means always parallel to the axis; 1 means always horizontal direction; 2 means always perpendicular to the axis; 3 means always vertical direction

# tcl: Can also be used to set the length of the tick marks, but the unit is different from tck. Its default value is -0.5

# bty: The parameter value is a string type, used to limit the type of the plot border. If the value of bty is "o" (default value: i.e., borders on all four sides), "l", "7", "c", "u", or "]" among any one, the corresponding border type is similar to the shape of the letter.

# If the value of bty is "n", it means no border.

# mar: A numerical vector of the form c(bottom, left, top, right) which gives the number of lines of margin to be specified on the four sides of the plot. The default is c(5, 4, 4, 2) + 0.1.

# xpd: Logical parameter or NA, FALSE means only display text within the plot region, TRUE means only display text within the figure region, NA means display text within the entire device region;

barplot(as.matrix(t(ciber.res)),

border = NA, # No border for the bars

names.arg = rep("",nrow(ciber.res)), # No sample names on the x-axis

yaxt = "n", # Do not draw the y-axis for now

ylab = "Relative percentage", # Modify the y-axis label

col = mycol) # Use the rainbow color palette

axis(side = 2, at = c(0,0.2,0.4,0.6,0.8,1), # Add percentages to the y-axis

labels = c("0%","20%","40%","60%","80%","100%"))

legend(par("usr")[2]-2, # Adjust the legend position according to the actual plot (here -20)

par("usr")[4],

legend = colnames(ciber.res),

xpd = T,

fill = mycol,

cex = 0.7,

border = NA,

y.intersp = 1,

x.intersp = 0.2,

bty = "n")

dev.off() # Close the plot

## 8.5.2 cibersort_GSE57065 boxplot----

library(tidyverse)

a <- CIBERSORT_Score_GSE57065 %>% as.data.frame() # Pipeline function (Ctrl + Shift + M)

b <- GSE57065_targets

identical(rownames(a),rownames(b)) # Check if the row names of the two tables are in the same order

b$group <- as.factor(b$group) # Convert the group column in data frame b to a factor type

class(b$group)

# a$group <- b$group # Add the group column from table b to table a; note that both a and b must be data frames

# Remove cells with zero abundance

ciber.res <- CIBERSORT_Score_GSE57065[,colSums(CIBERSORT_Score_GSE57065) > 0] # Remove cells with zero abundance

a <- a [,colnames(ciber.res)] %>% rownames_to_column("sample")

a$group <- b$group # Add the group column from table b to table a; note that both a and b must be data frames

library(ggsci)

library(tidyr)

library(ggpubr)

# b <- b[rownames(a),]

# identical(rownames(a),rownames(b))

# # Take the intersection of row names

# comgene <- intersect(rownames(a),rownames(b))

# a <- a[comgene,]

# class(a)# Check data type

# class(comgene)

# b <- b[comgene,]

# identical(rownames(a),rownames(b))# Check if multiple elements are identical

b <- gather(a,key=CIBERSORT,value = Proportion,-c(group,sample)) # Convert wide data to long data (code: 2119)

# pivot_wider(names_from = GSE, values_from = logFC) # Convert long data to wide data (code: 1802)

pdf("./CIBERSORT_GSE57065/boxplot_GSE57065.pdf")

ggboxplot(b, x = "CIBERSORT", y = "Proportion",

fill = "group", palette = "lancet")+

stat_compare_means(aes(group = group),

method = "wilcox.test",

label = "p.signif",

symnum.args=list(cutpoints = c(0, 0.001, 0.01, 0.05, 1),

symbols = c("***", "**", "*", "ns")))+

theme(text = element_text(size=10), # Font size of all text in the plot

axis.text.x = element_text(angle=45, hjust=1))

# hjust =0 means left-aligned, hjust =1 means right-aligned, hjust =0.5 means centered

dev.off()

CB5 <- ggboxplot(b, x = "CIBERSORT", y = "Proportion",

fill = "group", palette = "lancet")+

stat_compare_means(aes(group = group),

method = "wilcox.test",

label = "p.signif",

symnum.args=list(cutpoints = c(0, 0.001, 0.01, 0.05, 1),

symbols = c("***", "**", "*", "ns")))+

theme(text = element_text(size=10),

axis.text.x = element_text(angle=45, hjust=1))

CB5

dev.off()

### 8.6 cibersort_GSE95233 barplot and boxplot----

## 8.6.1 cibersort_GSE95233 barplot----

rm(list=ls())

# options("BioC_mirror" = "http://mirrors.tuna.tsinghua.edu.cn/bioconductor/ ")

# if (!requireNamespace("e1071", quietly = TRUE)) {

# BiocManager::install("e1071", ask = F, update = F)

# }

library(e1071)

library(parallel)

library(preprocessCore)

library(tidyverse)

# if(!dir.exists("CIBERSORT")) dir.create("CIBERSORT")

setwd("F:/HelixCourses/GEOdatasets_spsis1/DEG_SPSIS/CIBERSORT")

source("./CIBERSORT.R")

if(!dir.exists("CIBERSORT_GSE95233"))dir.create("CIBERSORT_GSE95233")

# Assign the file name "LM22.txt" to sig_matrix, and the program will automatically read this file in the directory

sig_matrix <- "LM22.txt" # "LM22.txt" is the annotation file for 22 immune cells

load("F:/HelixCourses/GEOdatasets_spsis1/DEG_SPSIS/GSE95233/GSE95233_processed.Rda")

# write.table(GSE95233_anno, file = "GSE95233_anno.txt",sep="\t", quote=F, col.names=T,row.names = T)

mixture_file = 'GSE95233_anno.txt' # Expression data for the GSE137340 dataset (note to adjust the first row of the table)

# perm indicates the number of calculations; the more times, the more stable the result

res_cibersort <- CIBERSORT(sig_matrix,

mixture_file,

perm=100, # perm, the number of permutation tests, default is 0; significant p-values can be calculated when greater than 100

QN=TRUE) # QN, whether to normalize the input expression matrix

# Equivalent to calculating 100 times continuously to see if the result is random in 100 times

# Generate a normalization function normalize, create a function

normalize <- function(x){# (x - minimum of x) / (maximum of x - minimum of x)

return((x-min(x))/(max(x)-min(x)))}

# Normalize CIBERSORT scores using normalize

CIBERSORT_Score <- res_cibersort

CIBERSORT_Score_GSE95233 <- normalize(CIBERSORT_Score)

save(CIBERSORT_Score_GSE95233, CIBERSORT_Score, file = "./CIBERSORT_GSE95233/CIBERSORT_Score_GSE95233.Rdata") # Save intermediate file

load("./CIBERSORT_GSE95233/CIBERSORT_Score_GSE95233.Rdata")

CIBERSORT_Score_GSE95233<- CIBERSORT_Score_GSE95233[,1:22]

# colSums adds up the columns

ciber.res <- CIBERSORT_Score_GSE95233[,colSums(CIBERSORT_Score_GSE95233) > 0] # Remove cells with zero abundance

# Visualization (by Teacher Achim)

mycol <- ggplot2::alpha(rainbow(ncol(ciber.res)), 0.7) # Create a rainbow color palette (with 70% transparency)

# alpha: Add transparency, the range of alpha is from 0 to 1, 1 is the original color, the larger the number, the darker the color

par(bty="o", mgp = c(2.5,0.3,0), mar = c(2.1,4.1,2.1,10.1),tcl=-.25,las = 1,xpd = F)

# las: Can only be one of 0, 1, 2, 3, used to indicate the direction of the tick mark values. 0 means always parallel to the axis; 1 means always horizontal direction; 2 means always perpendicular to the axis; 3 means always vertical direction

# tcl: Can also be used to set the length of the tick marks, but the unit is different from tck. Its default value is -0.5

# bty: The parameter value is a string type, used to limit the type of the plot border. If the value of bty is "o" (default value: i.e., borders on all four sides), "l", "7", "c", "u", or "]" among any one, the corresponding border type is similar to the shape of the letter.

# If the value of bty is "n", it means no border.

# mar: A numerical vector of the form c(bottom, left, top, right) which gives the number of lines of margin to be specified on the four sides of the plot. The default is c(5, 4, 4, 2) + 0.1.

# xpd: Logical parameter or NA, FALSE means only display text within the plot region, TRUE means only display text within the figure region

# xpd: Logical parameter or NA, FALSE means only display text within the plot region,

# TRUE means only display text within the figure region, NA means display text within the entire device region

barplot(as.matrix(t(ciber.res)),

border = NA, # No border for the bars

names.arg = rep("",nrow(ciber.res)), # No sample names on the x-axis

yaxt = "n", # Do not draw the y-axis for now

ylab = "Relative percentage", # Modify the y-axis label

col = mycol) # Use the rainbow color palette

axis(side = 2, at = c(0,0.2,0.4,0.6,0.8,1), # Add percentages to the y-axis

labels = c("0%","20%","40%","60%","80%","100%"))

legend(par("usr")[2]-2, # Adjust the legend position according to the actual plot (here -20)

par("usr")[4],

legend = colnames(ciber.res),

xpd = TRUE,

fill = mycol,

cex = 0.7,

border = NA,

y.intersp = 1,

x.intersp = 0.2,

bty = "n")

dev.off() # Close the plot

## 8.6.2 cibersort_GSE95233 boxplot----

library(tidyverse)

a <- CIBERSORT_Score_GSE95233 %>% as.data.frame()

b <- GSE95233_targets

identical(rownames(a),rownames(b)) # Check if the row names of the two tables are in the same order

b$group <- as.factor(b$group) # Convert the group column in data frame b to a factor type

class(b$group)

# a$group <- b$group # Add the group column from table b to table a; note that both a and b must be data frames

# Remove cells with zero abundance

ciber.res <- CIBERSORT_Score_GSE95233[,colSums(CIBERSORT_Score_GSE95233) > 0] # Remove cells with zero abundance

a <- a [,colnames(ciber.res)] %>% rownames_to_column("sample")

a$group <- b$group # Add the group column from table b to table a; note that both a and b must be data frames

library(ggsci)

library(tidyr)

library(ggpubr)

# b <- b[rownames(a),]

# identical(rownames(a),rownames(b))

# # Take the intersection of row names

# comgene <- intersect(rownames(a),rownames(b))

# a <- a[comgene,]

# class(a)# Check data type

# class(comgene)

# b <- b[comgene,]

# identical(rownames(a),rownames(b))# Check if multiple elements are identical

b <- gather(a,key=CIBERSORT,value = Proportion,-c(group,sample)) # Convert wide data to long data (code: 2119)

# pivot_wider(names_from = GSE, values_from = logFC) # Convert long data to wide data (code: 1802)

pdf("./CIBERSORT_GSE95233/boxplot_gse95233.pdf")

ggboxplot(b, x = "CIBERSORT", y = "Proportion",

fill = "group", palette = "lancet")+

stat_compare_means(aes(group = group),

method = "wilcox.test",

label = "p.signif",

symnum.args=list(cutpoints = c(0, 0.001, 0.01, 0.05, 1),

symbols = c("***", "**", "*", "ns")))+

theme(text = element_text(size=10), # Font size of all text in the plot

axis.text.x = element_text(angle=45, hjust=1))

# hjust =0 means left-aligned, hjust =1 means right-aligned, hjust =0.5 means centered

dev.off()

CB6 <- ggboxplot(b, x = "CIBERSORT", y = "Proportion",

fill = "group", palette = "lancet")+

stat_compare_means(aes(group = group),

method = "wilcox.test",

label = "p.signif",

symnum.args=list(cutpoints = c(0, 0.001, 0.01, 0.05, 1),

symbols = c("***", "**", "*", "ns")))+

theme(text = element_text(size=10),

axis.text.x = element_text(angle=45, hjust=1))

CB6

dev.off()

#### 9 GO|KEGG ----

rm(list = ls())

setwd("F:/HelixCourses/GEOdatasets_spsis1/DEG_SPSIS")

# options("repos" = c(CRAN = "https://mirrors.tuna.tsinghua.edu.cn/CRAN/ "))

# options("BioC_mirror" = "http://mirrors.tuna.tsinghua.edu.cn/bioconductor/ ")

# BiocManager::install("clusterProfiler")

# BiocManager::install("org.Hs.eg.db")

devtools::install_local("tinyarray-master.zip",upgrade = FALSE,dependencies = TRUE)

library(tinyarray)

library(clusterProfiler)

library(org.Hs.eg.db)

library(tidyverse)

if(!dir.exists("GO_KEGG"))dir.create("GO_KEGG")

input <- read_tsv("F:/HelixCourses/GEOdatasets_spsis1/DEG_SPSIS/RRP/all_signi_result_P_F.tsv")

head(input)

# Convert Gene Symbol to Entrez ID

gene.df <- bitr(input$SYMBOL,

fromType = "SYMBOL",

toType = c("ENSEMBL", "ENTREZID"),

# drop = TRUE, # Remove NA and unmatched gene names

OrgDb = org.Hs.eg.db)

gene.df_1 <- bitr(input$SYMBOL,

fromType = "SYMBOL",

toType = "ENTREZID",

# drop = TRUE, # Remove NA and unmatched gene names

OrgDb = org.Hs.eg.db)

# rm(gene1)

gene1 <- gene.df$ENTREZID

gene <- gene1[!duplicated(gene1)]

class(gene)

### Start GO and KEGG analysis

# GO enrichment analysis

ego <- enrichGO(gene = gene,

OrgDb = org.Hs.eg.db,

ont = "ALL", # Default is "MF", ALL means all

pvalueCutoff =0.05,

qvalueCutoff =0.05,

readable = TRUE)

head(ego)

table(ego$ONTOLOGY)

ego_result_df <- as.data.frame(ego)

library(enrichplot)

??dotplot

enrichplot::dotplot(ego,x = "GeneRatio", color = "p.adjust", size = "Count", # Default parameters

showCategory =5,# Show only the top 5

split="ONTOLOGY") + # Split by ONTOLOGY type

facet_grid(ONTOLOGY~., scale='free') # Split the plot by ONTOLOGY type

dev.off()

# Write GO enrichment results to a file

# write.table(ego,file="./RRP/GO_result.txt",sep="\t", quote=FALSE,row.names = FALSE)

# KEGG enrichment analysis

kk <- enrichKEGG(gene = gene,

organism = "hsa",

# keyType = "kegg",

pvalueCutoff =0.9999,

qvalueCutoff =0.9999)

head(kk)

# Write KEGG enrichment results to a file

# write.table(kk,file="./RRP/KEGG_result.txt",sep="\t",quote=FALSE,row.names = FALSE)

# Save results in Rda format

save(ego, kk, file = "./RRP/clusterProfiler_result.Rda")

### Start visualization

# Load GO and KEGG results

load(file = "./RRP/clusterProfiler_result.Rda")

## Bar plot

## Create directories (data and figures)

# if(!dir.exists("data")) dir.create("data")

pdf("./GO_KEGG/GO_barplot.pdf", 20, 8)

barplot(ego, showCategory = 10,color = "pvalue")

dev.off()

pdf("./GO_KEGG/KEGG_barplot.pdf", 20, 8)

barplot(kk, showCategory = 10,color = "pvalue")

dev.off()

# Dot plot

pdf("./GO_KEGG/GO_dotplot.pdf", 20, 8)

dotplot(ego, showCategory = 10,color = "pvalue")

dev.off()

# Faceted Dot Plot

pdf("./GO_KEGG/GO_facet_dotplot.pdf", width = 20, height = 8)

dotplot(ego,

x = "GeneRatio", color = "p.adjust", size = "Count", # Default parameters (x and color can be changed based on ego content)

showCategory = 5, # Display top 5 categories

split = "ONTOLOGY") + # Split by ONTOLOGY type

facet_grid(ONTOLOGY ~ ., scale = 'free') # Facet by ONTOLOGY type with free scales

dev.off()

pdf("./GO_KEGG/KEGG_dotplot.pdf", width = 20, height = 8)

dotplot(kk, showCategory = 10, color = "pvalue")

dev.off()

####

#### 10 WGCNA ----

### 10 WGCNA

#### 10.1 Data Processing

### 10.1.1 Loading Data (GSE69528) ###

rm(list = ls())

input <- load("F:\\Decoding Helix Courses\\GEOdatasets_spsis1\\DEG_SPSIS\\GSE69528\\GSE69528_processed.Rda")

input

dim(GSE69528_anno)

## 10.1.1 Selecting Genes by Standard Deviation ###

# # Calculate standard deviation for each gene

# GSE69528_anno_sd = apply(GSE69528_anno, 1, sd) # 1 for rows, 2 for columns

#

# ## Sort genes by standard deviation in descending order

# GSE69528_anno_sd_sorted = order(GSE69528_anno_sd, decreasing = TRUE)

# # F:/Decoding Helix Courses/Full Code Breakthrough Bioinformatics Data Mining/Level 2/L62_Codes/6-2-GSEA_GSVA_Analysis/Step01-GSEA_Analysis.R Code: P34

# # geneList = sort(geneList, decreasing = TRUE)

#

# ## Select top 5000 genes with the highest standard deviation

# GSE69528_anno_num = GSE69528_anno_sd_sorted[1:5000]

# ## Extract genes from the expression matrix

# GSE69528_anno_filter = GSE69528_anno[GSE69528_anno_num,]

### 10.1.1 Using All Data ###

## Transpose the expression matrix

WGCNA_matrix = t(GSE69528_anno) # Rows become samples, columns become genes

dim(WGCNA_matrix)

## Save the filtered data

if (!dir.exists("WGCNA")) dir.create("WGCNA")

save(WGCNA_matrix, file = "WGCNA/WGCNA_matrix.Rda")

### 10.1.2 Removing Samples with Many Missing Values ###

rm(list = ls())

# Load the expression matrix

load(file = "WGCNA/WGCNA_matrix.Rda")

# Load WGCNA package

library(WGCNA)

# Check if there are too many missing samples or genes

datExpr0 = WGCNA_matrix

dim(datExpr0)

gsg = goodSamplesGenes(datExpr0, verbose = 3)

# Determine if filtering is needed, TRUE means no filtering, FALSE means filtering

gsg$allOK

# When gsg$allOK is TRUE, the following code does not run; if FALSE, filter samples

if (!gsg$allOK)

{

# Print removed gene and sample names

if (sum(!gsg$goodGenes) > 0)

printFlush(paste("Removing genes:", paste(names(datExpr0)[!gsg$goodGenes], collapse = ", ")));

if (sum(!gsg$goodSamples) > 0)

printFlush(paste("Removing samples:", paste(rownames(datExpr0)[!gsg$goodSamples], collapse = ", ")));

# Extract retained genes and samples

datExpr0 = datExpr0[gsg$goodSamples, gsg$goodGenes]

}

### 10.1.3 Identifying and Removing Outlier Samples via Sample Clustering ###

sampleTree = hclust(dist(datExpr0), method = "average") # Perform hierarchical clustering using average linkage

# Plot the sample clustering dendrogram to identify outliers

sizeGrWindow(30, 9)

pdf(file = "WGCNA/figures/sampleClustering.pdf", width = 30, height = 9)

par(cex = 0.6)

par(mar = c(0, 4, 2, 0))

plot(sampleTree, main = "Sample clustering to detect outliers", sub = "", xlab = "", cex.lab = 1.5,

cex.axis = 1.5, cex.main = 2)

# Draw a red line at height h to indicate where to cut

abline(h = 140, col = "red") # Cut at height 140 based on the dendrogram

dev.off()

# Remove outlier samples based on the cut height

clust = cutreeStatic(sampleTree, cutHeight = 140, minSize = 10)

table(clust)

# clust

# 0 1

# 0 is to be removed, 1 is to be kept

# Extract samples belonging to cluster 1

keepSamples = (clust == 1)

datExpr = datExpr0[keepSamples, ]

dim(datExpr)

# Record the number of genes and samples for later visualization

nGenes = ncol(datExpr) # Number of genes

nSamples = nrow(datExpr) # Number of samples

save(datExpr, nGenes, nSamples, file = "WGCNA/WGCNA_input.Rda")

#### 10.2 Step-by-Step WGCNA

# Clear the environment

rm(list = ls())

# Load necessary packages

library(WGCNA)

# Enable multi-threading

enableWGCNAThreads()

# Load the expression matrix

load("WGCNA/WGCNA_input.Rda")

### 10.2.1 Selecting Soft Threshold ###

sizeGrWindow(9, 5)

par(mfrow = c(1, 2))

powers = c(c(1:10), seq(from = 12, to = 20, by = 2))

# powers = c(1:20) # Power exponent range from 1 to 20

# RpowerTable_1 = pickSoftThreshold(datExpr, powerVector = powers) is equivalent to the following

RpowerTable = pickSoftThreshold(datExpr, powerVector = powers)[[2]]

sft = pickSoftThreshold(datExpr, powerVector = powers, verbose = 5)

# If R^2 does not reach 0.85 or above, choose an empirical beta value.

# For nSamples < 20, soft threshold at 9; for 20 <= nSamples < 30, choose 8; for 30 <= nSamples < 40, choose 7

# Visualize results to determine the soft threshold

cex1 = 0.7

pdf(file = "WGCNA/figures/softThresholding.pdf", width = 14)

par(mfrow = c(1, 2))

plot(RpowerTable[, 1], -sign(RpowerTable[, 3]) * RpowerTable[, 2], xlab = "Soft Threshold (power)", ylab = "Scale Free Topology Model Fit, signed R^2", type = "n", main = paste("Scale independence"))

text(RpowerTable[, 1], -sign(RpowerTable[, 3]) * RpowerTable[, 2], labels = powers, cex = cex1, col = "red")

abline(h = 0.85, col = "red")

plot(RpowerTable[, 1], RpowerTable[, 5], xlab = "Soft Threshold (power)", ylab = "Mean Connectivity", type = "n", main = paste("Mean connectivity"))

text(RpowerTable[, 1], RpowerTable[, 5], labels = powers, cex = cex1, col = "red")

dev.off()

## Verify Scale-Free Network: Ensure the soft threshold is reasonable

# softpower = 10

softpower = sft$powerEstimate

ADJ = abs(cor(datExpr, use = "p"))^softpower # cor(): Calculate pairwise correlations

head(ADJ)[, 1:6]

# "p" indicates pairwise deletion; cor can only calculate correlation coefficients, need cor.mtset to calculate p-values

# na.rm = TRUE parameter; remove missing values during calculation for valid results

k = as.vector(apply(ADJ, 2, sum, na.rm = TRUE))

pdf(file = "WGCNA/figures/scaleFree.pdf", width = 14)

par(mfrow = c(1, 2))

hist(k)

scaleFreePlot(k, main = "Check scale free topology")

dev.off()

### 10.2.2 Calculate Adjacency, Topological, and Dissimilarity Matrices ###

## Calculate adjacency matrix

adjacency = adjacency(datExpr, power = softpower)

adjacency[1:6, 1:6]

## Calculate TOM (Topological Overlap Matrix)

TOM = TOMsimilarity(adjacency)

TOM[1:6, 1:6]

## Calculate dissimilarity

dissTOM = 1 - TOM

dissTOM[1:6, 1:6]

# The above code is found on P4642~P4643

### 10.2.3 Construct Gene Hierarchical Clustering Tree and Network Modules ###

## 10.2.3.1 Construct Gene Hierarchical Clustering Tree

# Preliminary module clustering analysis: (Cluster genes based on dissimilarity)

library(flashClust)

geneTree = flashClust(as.dist(dissTOM), method = "average")

# The above two lines are equivalent to geneTree = hclust(as.dist(dissTOM), method = "average")

# Plot the hierarchical clustering tree

pdf(file = "WGCNA/figures/GeneClusterTOM.pdf")

plot(geneTree, xlab = "", sub = "", main = "Gene clustering on TOM-based", labels = FALSE, hang = 0.04)

dev.off()

## 10.2.3.2 Construct Network Modules

# Build preliminary gene modules

# Set minimum module size to 50 genes

minModuleSize = 50

# Dynamically cut the tree to identify network modules

dynamicMods = cutreeDynamic(dendro = geneTree, # hclust result from clustering

distM = dissTOM, # Dissimilarity matrix

deepSplit = 0,

pamRespectsDendro = FALSE,

minClusterSize = minModuleSize) # Set minimum genes per module to 30

table(dynamicMods)

## 10.2.3.3 Plot Hierarchical Clustering Tree and Network Modules

# Convert labels to colors

# standardColors(20)

dynamicColors = labels2colors(dynamicMods) # Map numerical labels to module colors

table(dynamicColors) # View which modules and colors genes are clustered into

pdf(file = "WGCNA/figures/DynamicTreeCut.pdf", width = 9, height = 5)

plotDendroAndColors(dendro = geneTree,

colors = dynamicColors,

groupLabels = "Dynamic Tree Cut",

dendroLabels = FALSE, hang = 0.03,

addGuide = TRUE,

main = "Gene dendrogram and module colors")

dev.off()

# Rename the merged modules

moduleColors = mergedColors

colorOrder = c("grey", standardColors(50))

moduleLabels = match(moduleColors, colorOrder) - 1

MEs = mergedMEs

MEDiss2 = 1 - cor(MEs) # Calculate and convert module dissimilarity

METree2 = flashClust(as.dist(MEDiss2), method = "average") # Cluster the merged modules

# Plot clustering results

pdf(file = "WGCNA/figures/MECombined.pdf", width = 12, height = 5)

par(mfrow = c(1, 2))

plot(METree1, xlab = "", sub = "", main = "Clustering of ME before combined") # METree1 is from dynamic tree cut modules

abline(h = MEDissThres, col = "red") # Dissimilarity threshold at 0.25

plot(METree2, xlab = "", sub = "", main = "Clustering of ME after combined") # METree2 is after merging

dev.off()

pdf(file = "WGCNA/figures/MergedDynamics.pdf", width = 10, height = 4)

plotDendroAndColors(dendro = geneTree, # Cut tree

colors = cbind(dynamicColors, mergedColors), # Combine both methods' module colors

groupLabels = c("Dynamic Tree Cut", "Merged Dynamics"),

dendroLabels = FALSE,

hang = 0.03,

addGuide = TRUE,

guideHang = 0.05,

main = "Gene Dendrogram and module colors")

dev.off()

# Number of genes in each module

write.table(table(moduleColors), "WGCNA/MEgeneCount.txt", quote = FALSE, row.names = FALSE)

# Save network information

moduleColors = mergedColors

# standardColors(100)

colorOrder = c("grey", standardColors(50))

moduleLabels = match(moduleColors, colorOrder) - 1 # match: Match two vectors, return the position of the second vector in the first

MEs = mergedMEs

save(MEs, moduleLabels, softpower, moduleColors, geneTree, file = "WGCNA/Step_by_step_buildnetwork.rda")

## Plot Module Correlations

rm(list = ls())

library(WGCNA)

load("WGCNA/WGCNA_input.Rda")

load(file = "WGCNA/Step_by_step_buildnetwork.rda")

MEs = orderMEs(MEs)

sizeGrWindow(5, 7.5)

pdf(file = "WGCNA/figures/moduleCor.pdf", width = 5, height = 7.5)

par(cex = 0.9)

plotEigengeneNetworks(MEs, "", marDendro = c(0, 4, 1, 2), marHeatmap = c(3, 4, 1, 2), cex.lab = 0.8, xLabelsAngle = 90)

dev.off()

# Enable multi-threading for WGCNA

enableWGCNAThreads()

## TOMplot

dissTOM = 1 - TOMsimilarityFromExpr(datExpr, power = softpower) # Consistent with earlier dissimilarity calculation

head(dissTOM)[, 1:6]

# The above code is found on P4514~P4519

nSelect = 400

# Randomly select 400 genes for visualization, set seed for reproducibility

set.seed(10)

select = sample(nGenes, size = nSelect)

selectTOM = dissTOM[select, select] # Extract TOM matrix for selected genes

# Re-cluster the selected genes

selectTree = hclust(as.dist(selectTOM), method = "average")

mergedColors = moduleColors

selectColors = mergedColors[select] # Extract module colors for the 400 selected genes

# Open a plotting window

sizeGrWindow(9, 9)

pdf(file = "WGCNA/figures/TOMplot.pdf", width = 9, height = 9)

# Enhance the TOM plot by exponentiating

plotDiss = selectTOM^7

diag(plotDiss) = NA # Set diagonal to NA

TOMplot(plotDiss, selectTree, selectColors,

main = "Network heatmap plot, selected genes")

dev.off()

## Adjust color scheme for better visualization

??colorpanel

library(gplots)

myheatcol = colorpanel(250, 'red', "orange", 'lemonchiffon')

pdf(file = "TOM_plot0.pdf")

plotDiss = selectTOM^7

diag(plotDiss) = NA

TOMplot(plotDiss, selectTree, col = myheatcol, selectColors, main = "Network heatmap plot, selected genes")

dev.off()

### 10.3 Clinical Phenotype Module Construction ###

# Clear environment variables

rm(list = ls())

# Load necessary packages

library(WGCNA)

# Load expression matrix

load("WGCNA/WGCNA_input.Rda")

load("GSE69528/GSE69528_processed.Rda")

## 10.3.1 Load Clinical Information

# clinical = read.table("data/clinical.txt", stringsAsFactors = TRUE, header = TRUE, row.names = 1, na.strings = "", sep = "\t")

# clinical <- GSE69528_targets[c(1,4)]

library(tidyverse)

clinical <- GSE69528_targets %>% rownames_to_column()

clinical1 <- clinical[c(2, 5)] %>% mutate("sepsis" = group) %>%

column_to_rownames("sample_id") %>%

set_names("Control", "Sepsis")

datExpr <- as.data.frame(datExpr)

clinical2 <- clinical1[rownames(datExpr), ] %>% as.data.frame()

# write.table(clinical2, file = "WGCNA/clinical2.txt", row.names = TRUE, col.names = TRUE, sep = "\t", quote = FALSE)

clinical = read.table("WGCNA/clinical2.txt", stringsAsFactors = FALSE, header = TRUE, row.names = 1, na.strings = "", sep = "\t")

# View clinical information

head(clinical)

str(clinical)

# Preprocess the expression matrix

# datTraits = as.data.frame(do.call(cbind, lapply(clinical, as.factor)))

# rownames(datTraits) = rownames(clinical)

datTraits <- clinical

dim(datTraits)

head(datTraits)

## 10.3.2 Cluster Samples

head(datExpr)[, 1:5]

sampleTree2 = hclust(dist(datExpr), method = "average")

plot(sampleTree2)

## 10.3.3 Convert Clinical Information to Colors

# Convert clinical information to colors,

## 10.3.3 Convert sample clinical information to colors

# Convert clinical information to colors, white represents low, red represents high, gray represents missing

traitColors = numbers2colors(datTraits, signed = FALSE)

# Step03-Step_by_Step_for_WGCNA.R code: 78

# dynamicColors = labels2colors(dynamicMods) # Map numeric labels to modules

table(traitColors)

pdf(file = "WGCNA/figures/Sample_dendrogram_and_trait_heatmap.pdf", width = 24);

## 10.3.4 Sample clustering plot and sample trait heatmap

plotDendroAndColors(sampleTree2,

traitColors,

groupLabels = names(datTraits),

main = "Sample dendrogram and trait heatmap")

dev.off()

#### 10.3.5 Network analysis

###### Relationship between gene modules and clinical information

# Load the constructed network

load(file = "WGCNA/Step_by_step_buildnetwork.rda")

# 10.3.5.1 Sort the module eigengenes matrix

MEs=orderMEs(MEs)

# 10.3.5.2 Calculate the correlation between module eigengenes matrix and sample information matrix.

moduleTraitCor=cor(MEs, datTraits, use="p")

# write.table(file="data/Step04-modPhysiological.cor.xls",moduleTraitCor,sep="\t",quote=F)

moduleTraitPvalue=corPvalueStudent(moduleTraitCor, nSamples)

# write.table(file="data/Step04-modPhysiological.p.xls",moduleTraitPvalue,sep="\t",quote=F)

# 10.3.5.3 Visualize the correlation matrix and p-values using labeledHeatmap().

pdf(file="WGCNA/figures/Module_trait_relationships.pdf",width=9,height=7)

textMatrix=paste(signif(moduleTraitCor,2),"\n(",signif(moduleTraitPvalue,1),")",sep="")

head(textMatrix)

dim(textMatrix)=dim(moduleTraitCor)

# Heatmap of module-trait relationships

labeledHeatmap(Matrix=moduleTraitCor,# Core parameter: correlation matrix between modules and phenotypes

xLabels=colnames(datTraits),

yLabels=names(MEs),

ySymbols=names(MEs),

colorLabels=FALSE,

colors=blueWhiteRed(50),

textMatrix=textMatrix,

setStdMargins=FALSE,

cex.text=0.5,

cex.lab=0.5,

zlim=c(-1,1),

main=paste("Module-trait relationships"))

dev.off()

### 10.4 Batch output of trait-module scatter plots and module genes ###

# Detailed analysis of different modules and gene traits

## Matrix 1: Construct correlation coefficients and p-values between each gene and module

modNames = substring(names(MEs), 3) # Extract column names of MEs starting from the third character

geneModuleMembership = as.data.frame(cor(datExpr, MEs, use = "p")) # Calculate correlation coefficients

#### Check which genes are most correlated with a specific trait

a <- geneModuleMembership

head(a)

a <- a %>% rownames_to_column()

MMPvalue = as.data.frame(corPvalueStudent(as.matrix(geneModuleMembership), nSamples)) # Calculate p-values of correlations

names(geneModuleMembership) = paste("MM", modNames, sep="") # Rename columns of geneModuleMembership

names(MMPvalue) = paste("p.MM", modNames, sep="") # Rename columns of MMPvalue

## Matrix 2: Construct correlation coefficients and p-values between each gene and clinical traits

traitNames=names(datTraits) # Get column names

geneTraitSignificance = as.data.frame(cor(datExpr, datTraits, use = "p")) # use = "p": pairwise deletion in cor() function

GSPvalue = as.data.frame(corPvalueStudent(as.matrix(geneTraitSignificance), nSamples))

names(geneTraitSignificance) = paste("GS.", traitNames, sep="")

names(GSPvalue) = paste("p.GS.", traitNames, sep="")

## Extract key module core genes: Method 1

# Extract genes with GS.Sepsis > 0.2 and MM.blue > 0.8

FilterGenes= abs(geneTraitSignificance$GS.Sepsis)> .2 & abs(geneModuleMembership$MMblue)>.8

Genes <- colnames(datExpr)

FilterGenes_name <- Genes[FilterGenes] # Extract gene names where logical condition is TRUE

write.table(FilterGenes_name,

file ="F:\\Decoding Helix Courses\\GEOdatasets_spsis1\\DEG_SPSIS\\WGCNA\\Module and gene\\FilterGenes.txt",

sep="\t",row.names=TRUE,col.names=TRUE,quote=FALSE)

## Extract key module core genes: Method 2

# Select MMblue module: |MM|>0.8 & |GS|>0.2

hub<- abs(geneModuleMembership$MMblue)>0.8 & abs(geneTraitSignificance$GS.Sepsis)>0.2

table(hub)

# FALSE TRUE

# 10892 616

identical(FilterGenes,hub)

hub_name <- dimnames(data.frame(datExpr))[[2]][hub]

identical(FilterGenes_name,hub_name)

## Upon inspection, FilterGenes_name and hub_name differ because:

## hub_name contains "." in some gene names, while FilterGenes_name contains "-" in some gene names

## Therefore, use: gsub('\\.', '-', x); gsub('[.]', '-', x); gsub(".", "-", x, fixed = TRUE)

hub_name <- gsub('\\.', '-', hub_name)

identical(FilterGenes_name,hub_name)

# hub<-as.data.frame(dimnames(data.frame(datExpr))[[2]][hub])

# write.csv(hub, "./WGCNA/Module and gene/hubgene_GSMM_blue_sepsis.csv")

write.table(hub_name,

file ="F:\\Decoding Helix Courses\\GEOdatasets_spsis1\\DEG_SPSIS\\WGCNA\\Module and gene\\hubgene_GSMM_blue_sepsis.txt",

sep="\t",row.names=TRUE,col.names=TRUE,quote=FALSE)

# Different |MM| thresholds: |MM|>0.85 & |GS|>0.2

hub1<- abs(geneModuleMembership$MMblue)>0.85 & abs(geneTraitSignificance$GS.Sepsis)>0.2

table(hub1)

hub1<-as.data.frame(dimnames(data.frame(datExpr))[[2]][hub1])

# write.csv(hub1, "./WGCNA/Module and gene/hubgene1_GSMM_blue_sepsis.csv")

write.table(hub1,

file ="F:\\Decoding Helix Courses\\GEOdatasets_spsis1\\DEG_SPSIS\\WGCNA\\Module and gene\\hubgene1_GSMM_blue_sepsis.txt",

sep="\t",row.names=FALSE,col.names=TRUE,quote=FALSE)

# It was found that 41 genes do not belong to this module: See "WGCNA（6）：筛选 hub gene" in browser bookmarks

# Handling method as follows:

module = "blue"

column = match(module, modNames)

moduleGenes = moduleColors==module

blue_module<-as.data.frame(dimnames(data.frame(datExpr))[[2]][moduleGenes])

names(blue_module)="genename"

MM<-abs(geneModuleMembership[moduleGenes,column])

GS<-abs(geneTraitSignificance[moduleGenes, 2])

blue_MMGS<-as.data.frame(cbind(MM,GS))

rownames(blue_MMGS)=blue_module$genename

hub_b<-abs(blue_MMGS$MM)>0.8&abs(blue_MMGS$GS)>0.2

table(hub_b)

# FALSE TRUE

# 1755 424

blue_hub_b <- subset(blue_MMGS, abs(blue_MMGS$MM)>0.8 & abs(blue_MMGS$GS)>0.2)

write.csv(blue_hub_b,

file ="F:\\Decoding Helix Courses\\GEOdatasets_spsis1\\DEG_SPSIS\\WGCNA\\Module and gene\\hubgene_MMGS_blue.csv")

setwd("F:/Decoding Helix Courses/GEOdatasets_spsis1/DEG_SPSIS/WGCNA/figures/Module and clinical character map")

### 10.4.1 Batch output of trait-module scatter plots ###

for (trait in traitNames){

traitColumn=match(trait,traitNames)

for (module in modNames){

column = match(module, modNames)

moduleGenes = moduleColors==module

if (nrow(geneModuleMembership[moduleGenes,]) > 1){

outPdf=paste(trait, "_", module,".pdf",sep="")

pdf(file=outPdf,width=7,height=5) # Adjust figure dimensions

par(mfrow = c(1,1))

verboseScatterplot(abs(geneModuleMembership[moduleGenes, column]),

abs(geneTraitSignificance[moduleGenes, traitColumn]),

xlab = paste("Module Membership in", module, "module"),

ylab = paste("Gene significance for ",trait),

main = paste("Module membership vs. gene significance\n"),

cex.main = 1.2, cex.lab = 1.2, cex.axis = 1.2, col = module)

abline(v=0.8,h=0.2,col="red") # Highlight the region for extracting core genes

dev.off()

}

}

}

### 10.4.2 Batch output of genes in each module ###

for (mod in 1:nrow(table(moduleColors)))

{

modules = names(table(moduleColors))[mod]

probes = colnames(datExpr)

inModule = (moduleColors == modules)

modGenes = probes[inModule]

write.table(modGenes, file =paste0(modules,".txt"),sep="\t",row.names=FALSE,col.names=FALSE,quote=FALSE)

}

### 10.5 GO/KEGG analysis of genes in the blue module ###

rm(list=ls())

library(tinyarray)

library(clusterProfiler)

library(org.Hs.eg.db)

library(tidyverse)

setwd("F:/Decoding Helix Courses/GEOdatasets_spsis1/DEG_SPSIS/WGCNA")

if(!dir.exists("GO_KEGG"))dir.create("GO_KEGG")

input <- read.table(".\\figures\\Module and clinical character map\\blue.txt",

stringsAsFactors = FALSE, header = FALSE,

# row.names = 1,

na.strings = "",

sep = "\t")

head(input)

# Convert Gene Symbols to Entrez ID

gene.df <- bitr(input$V1,

fromType = "SYMBOL",

toType = c("ENSEMBL", "ENTREZID"),

# drop = TRUE, # Remove NA and genes that cannot be matched

OrgDb = org.Hs.eg.db)

# gene.df_1 <- bitr(input$SYMBOL,

# fromType = "SYMBOL",

# toType = "ENTREZID",

# # drop = TRUE, # Remove NA and genes that cannot be matched

# OrgDb = org.Hs.eg.db)

# rm(gene1)

gene1 <- gene.df$ENTREZID

gene <- gene1[!duplicated(gene1)]

class(gene)

head(gene)

### Start GO and KEGG analysis

# GO enrichment analysis

ego <- enrichGO(gene = gene,

OrgDb = org.Hs.eg.db,

ont = "ALL", # Default is "MF", ALL includes all

pvalueCutoff =0.05,

qvalueCutoff =0.05,

readable = TRUE)

head(ego)

table(ego$ONTOLOGY)

ego_result_df <- as.data.frame(ego) # Convert to data frame

dotplot(ego,x = "GeneRatio", color = "p.adjust", size = "Count", # Default parameters

showCategory =5,# Show top 5

split="ONTOLOGY") + # Split by ONTOLOGY type

facet_grid(ONTOLOGY~., scale='free') # Facet by ONTOLOGY type

dev.off()

# Save GO enrichment results to file

# write.table(ego,file="./RRP/GO_result.txt",sep="\t", quote=F,row.names = F)

# KEGG enrichment analysis

kk <- enrichKEGG(gene = gene,

organism = "hsa",

# keyType = "kegg",

pvalueCutoff =0.9999,

qvalueCutoff =0.9999)

head(kk)

# Save results as Rda format

save(ego, kk, file = "./GO_KEGG/clusterProfiler_result.Rda")

# Paginated dot plot

pdf("./GO_KEGG/GO_facet_dotplot.pdf", 20, 8)

dotplot(ego,

x = "GeneRatio", color = "p.adjust", size = "Count", # Default parameters (x and color can be changed based on ego content)

showCategory =5, # Show top 5

split="ONTOLOGY") + # Split by ONTOLOGY type

facet_grid(ONTOLOGY~., scale='free') # Facet by ONTOLOGY type

dev.off()

pdf("./GO_KEGG/KEGG_dotplot.pdf", 20, 8)

dotplot(kk, showCategory = 10,color = "pvalue")

dev.off()

### Intersection of results ###

rm(list=ls())

input <- read.table('F:\\Decoding Helix Courses\\GEOdatasets_spsis1\\DEG_SPSIS\\RRP\\all_signi_result_P_F.tsv')

FilterGenes <- read.table("F:\\Decoding Helix Courses\\GEOdatasets_spsis1\\DEG_SPSIS\\WGCNA\\Module and gene\\FilterGenes.txt")

hub <- read.csv("F:\\Decoding Helix Courses\\GEOdatasets_spsis1\\DEG_SPSIS\\WGCNA\\Module and gene\\hubgene_MMGS_blue.csv")

identical(hub$x,FilterGenes$x)

Comgene <- as.data.frame(intersect(hub$X,input$V1))

input_up <- read.table(".\\Venn\\all_signi_result_P_F_up.txt")

input_down <- read.table(".\\Venn\\all_signi_result_P_F_down.txt")

Comgene_up <- as.data.frame(intersect(hub$X,input_up$V1))

Comgene_down <- as.data.frame(intersect(hub$X,input_down$V1))

write.table(Comgene,

file ="F:\\Decoding Helix Courses\\GEOdatasets_spsis1\\DEG_SPSIS\\WGCNA\\Venn\\Comgene.txt",

sep="\t",

row.names=FALSE,

col.names=FALSE,

quote=FALSE)

write.table(Comgene_up,

file ="F:\\Decoding Helix Courses\\GEOdatasets_spsis1\\DEG_SPSIS\\WGCNA\\Venn\\Comgene_up.txt",

sep="\t",

row.names=FALSE,

col.names=FALSE,

quote=FALSE)

write.table(Comgene_down,

file ="F:\\Decoding Helix Courses\\GEOdatasets_spsis1\\DEG_SPSIS\\WGCNA\\Venn\\Comgene_down.txt",

sep="\t",

row.names=FALSE,

col.names=FALSE,

quote=FALSE)

#### 11 STRING analysis ----

# Perform STRING analysis using an online tool

rm(list = ls())

library(tidyverse)

setwd("F:/Decoding Helix Courses/GEOdatasets_spsis1/DEG_SPSIS/STRING/ELF1_miRNA")

miRNA <- read.table(".\\venn_ELF1_miRNA_3com.txt",header = TRUE)

lncRNA_miRNA <- read.table("lncRNA_miRNA_interaction_tiqu.txt",

header = TRUE,sep="\t",check.names=FALSE)

com_miRNA <- intersect(miRNA$GENE,lncRNA_miRNA$miRNAname)

com_lncRNA_miRNA <- lncRNA_miRNA %>% filter(lncRNA_miRNA$miRNAname %in% com_miRNA) %>% arrange(miRNAname,geneName)

com_lncRNA_miRNA_1 <- lncRNA_miRNA[lncRNA_miRNA$miRNAname %in% com_miRNA, ] %>% arrange(miRNAname,geneName)

identical(com_lncRNA_miRNA,com_lncRNA_miRNA_1)

a <- unique(com_lncRNA_miRNA$miRNAname)

# Extract duplicates

com_lncRNA_miRNA_dup <- com_lncRNA_miRNA[!duplicated(com_lncRNA_miRNA),]

# duplicated() identifies the first occurrence of duplicates in a vector or data frame and returns a logical vector indicating which elements (rows) are duplicates.

com_lncRNA_miRNA_dup_LINC <- com_lncRNA_miRNA_dup[duplicated(com_lncRNA_miRNA_dup$geneName)==TRUE,]

b <- table(com_lncRNA_miRNA_dup_LINC$geneName) %>% as.data.frame() %>% arrange(.,desc(Freq))

NEAT1_miRNA <- com_lncRNA_miRNA_dup_LINC[com_lncRNA_miRNA_dup_LINC$geneName=="NEAT1",]

XIST_miRNA <- com_lncRNA_miRNA_dup_LINC[com_lncRNA_miRNA_dup_LINC$geneName=="XIST",]

c <- intersect(lncRNA_miRNA_NEAT1$miRNAname,lncRNA_miRNA_XIST$miRNAname)

# Save data

write.table(b, file = "./lncRNA_miRNA_mRNA.txt",

sep = "\t",row.names = FALSE,col.names = TRUE,quote = FALSE)

write.table(lncRNA_miRNA_NEAT1, file = "./NEAT1_miRNA.txt",

sep = "\t",row.names = FALSE,col.names = TRUE,quote = FALSE)

#### 12 Validation analysis ----

rm(list = ls())

setwd("F:/Decoding Helix Courses/GEOdatasets_spsis1/DEG_SPSIS")

library(GEOquery)

library(readxl)

library(tidyverse)

# ## Read GSE13015 data

# GSE13015_sm<- getGEO(filename = "./Validated dataset/GSE13015/GSE13015-GPL6

# destdir = ".",

# getGPL = F )

# GSE13015_expr <- exprs(GSE13015_sm)

# head(GSE13015_expr)[, 1:6]

# GSE13015_pd <- pData(GSE13015_sm)

#

# ### create file

# setwd("F:/Decoding Helix Course/GEOdatasets_spsis1/DEG_SPSIS/Validated dataset/GSE13015")

# # dir.create("GSE13015")

# if(!dir.exists("GSE13015"))dir.create("GSE13015")

# getwd()

#

# ### save data

# save(GSE13015_sm, GSE13015_expr, GSE13015_pd,

# file = ".\\GSE13015.Rda")

# # load(".\\GSE13015_data.Rda")

#

# ### organize sample information

# GSE13015_targets <- GSE13015_pd %>% dplyr::select(2,1,14) %>%

# # dplyr::select(1, 2) %>%

# # separate(title, into = paste0("x", 1:4), sep = "_", remove = F) %>%

# # filter(!(x4 %in% c("D02", "D03"))) %>% # "F:/Decoding Helix Course/R language basics_7 days/Lesson 3/Lesson 3 live broadcast" code: P264

# mutate(sample_id = geo_accession,#add several columns

# #file_name = paste0(sample_id, ".CEL.gz"),

# sample_name = title,

# # sample_name = str_c(x1, x2, x3, sep = "_"),# Join multiple strings into a single string.

# # patient_id = str_extract(x2,"\\d+"),

# # patient_id = x3 ,

# patient_id = str_sub(GSE13015_pd$characteristics_ch1.4, start = 10, end = -1),

# #str_sub----Extract and replace substrings from a character vector. Extract from the last but one to the last three characters

# #GSE_num = "GSE69063",

# # The following line of code is shown in: "F:/Dr. Chen's medical records/Dr. Chen wants to be lazy/GSE84402" code: P240~P241

# # group_list <- ifelse(str_detect(pdata$source_name_ch1, "hepatocellular carcinoma"), "tumor", "normal")

# # group = ifelse(str_sub(source_name_ch1, 1, 1) == "P","Sepsis","Control")) %>%

# group = ifelse(str_detect(GSE13015_pd$characteristics_ch1.4, "Control"), "Control", "Sepsis")) %>%

# arrange(group,sample_id) %>%

# dplyr::select(sample_id:group) #%>%

# # dplyr::select(2,3,4,1)

#

# GSE13015_expr <- GSE13015_expr[ , GSE13015_targets$sample_id] %>% as.data.frame()

#

# ### View Data

# range(GSE13015_expr)

# exp <- log2(GSE13015_expr+1)

# range(expr)

# ### Error, indicating this method is not feasible

#

# ### Check how the original data was processed

# GSE13015_sm@phenoData@data$data_processing[1]

#

# #### Start from raw data extraction

# # Original data still contains negative values, so this dataset is abandoned

# Read GSE28750 data

GSE28750_sm<- getGEO(filename = "./Validated dataset/GSE28750/GSE28750_series_matrix.txt.gz",

destdir = ".",

getGPL = F )

GSE28750_expr <- exprs(GSE28750_sm)

head(GSE28750_expr)[, 1:6]

GSE28750_pd <- pData(GSE28750_sm)

### create file

setwd("F:/Decoding Helix Course/GEOdatasets_spsis1/DEG_SPSIS/Validated dataset/GSE28750")

# dir.create("GSE28750")

# if(!dir.exists("GSE28750"))dir.create("GSE28750")

getwd()

### save data

save(GSE28750_sm, GSE28750_expr, GSE28750_pd,

file = ".\\GSE28750.Rda")

# load(".\\GSE28750.Rda")

### organize sample information

GSE28750_targets <- GSE28750_pd %>% #dplyr::select(2,1,14) %>%

dplyr::select(2, 1) %>%

separate(title, into = paste0("x", 1:4), sep = "_", remove = F) %>%

# filter(!(x2 == "Post")) %>%

mutate(sample_id = geo_accession,#add several columns

#file_name = paste0(sample_id, ".CEL.gz"),

sample_name = title,

# sample_name = str_c(x1, x2, x3, sep = "_"),# Join multiple strings into a single string.

# patient_id = str_extract(x2,"\\d+"),

# patient_id = x3 ,

# patient_id = str_sub(GSE28750_pd$characteristics_ch1.4, start = 10, end = -1),

patient_id = str_c(x2, x3, sep = "_"),

#str_sub----Extract and replace substrings from a character vector. Extract from the last but one to the last three characters

#GSE_num = "GSE69063",

# The following line of code is shown in: "F:/Dr. Chen's medical records/Dr. Chen wants to be lazy/GSE84402" code: P240~P241

# group_list <- ifelse(str_detect(pdata$source_name_ch1, "hepatocellular carcinoma"), "tumor", "normal")

# group = ifelse(str_sub(source_name_ch1, 1, 1) == "P","Sepsis","Control")) %>%

# group = ifelse(str_detect(GSE28750_pd$characteristics_ch1.4, "Control"), "Control", "Sepsis")) %>%

group = ifelse(x1 == "Control", "Control", "Sepsis")) %>%

arrange(group,sample_id) %>%

dplyr::select(sample_id:group) #%>%

# dplyr::select(2,3,4,1)

GSE28750_expr <- GSE28750_expr[ , GSE28750_targets$sample_id] %>% as.data.frame()

### View Data

range(GSE28750_expr)

# exp <- log2(GSE28750_expr+1)

# range(expr)

### Evaluate data

if(!dir.exists("figures")) dir.create("figures")

pdf('./figures/GSE28750_boxplot.pdf',width = 8,height = 8)

# boxplot(GSE29450_rma, las = 2, col = rep(c("blue", "red"), each = 10))

# boxplot(GSE95233_expr, las=2, outline=FALSE,

# col = rep(c("blue", "red"), c(22,51)),

# main = "GSE95233_expr")

boxplot(GSE28750_expr, las=2, outline=FALSE,

# col = rep(c("blue", "red"), c(22,51)),

main = "GSE28750_expr")

dev.off()

source("F:\\Decoding Helix Course\\GEOdatasets_spsis1\\DEG_SPSIS\\Custom_Functions.R")

# BiocManager::install("genefilter", ask = F, update = F)

if (!requireNamespace("genefilter", quietly = TRUE))

BiocManager::install("genefilter", ask = F, update = F)

targets <- GSE28750_targets

pdf('./figures/GSE28750_PCA_new.pdf',width = 8,height = 8)

# PCA_new(GSE28750_expr, show_name = F) #default take top 500 genes with largest differences: i.e. ntop = 500

PCA_new(GSE28750_expr,

ntop = nrow(GSE28750_expr),

show_name = F)

# From the above two PCA plots, we can see that different numbers of genes have a big impact on the results

dev.off()

## Check how the original data was processed

GSE28750_sm@phenoData@data$data_processing[1]

# GSE28750_pd$data_processing[1] #this code is equivalent to the above code

## normalization

# # RMA normalized: original data standardization

# #Series matrix files txt or soft files data standardization

library(limma)

GSE28750_expr_n = normalizeBetweenArrays(GSE28750_expr)

boxplot(GSE28750_expr_n, las=2, outline=FALSE, main = "GSE28750_expr_n")

dev.off()

PCA_new(GSE28750_expr_n,

ntop = nrow(GSE28750_expr_n),

# ntop = 500, #number of top-ranked genes

# show_name = T,

group = GSE28750_targets$group)

dev.off()

# # From the above data processing method and boxplot/PCA plots, we know that GSE69528_expr doesn't need further standardization

# GSE69528_expr_n <- GSE69528_expr #this step is to keep all datasets consistent

### annotation

GSE28750_sm@annotation

## GPL570

# chooseCRANmirror()

# chooseBioCmirror()

# pkgs <- c("hgu133plus2.db","illuminaHumanv3.db")

# pkgs_in(pkgs)

library(hgu133plus2.db)

library(illuminaHumanv3.db)

ls("package:hgu133plus2.db")

p2s <- toTable(hgu133plus2SYMBOL) #%>%

# set_names("probe_id", "symbol")#rename columns

head(p2s)

## Custom function for chip annotation

annotate_expr <- function(expr, p2s, fun = mean){#three parameters: p2s has fixed column names

library(dplyr)

library(tibble)

# expr

expr <- expr %>%

as.data.frame() %>%

tibble::rownames_to_column("probe_id")#row names become a column called probe_id

# annotate

expr_annotated <- p2s %>%

dplyr::inner_join(expr, by = "probe_id") %>% #intersection of expr and p2s

na.omit() %>% #remove NA

dplyr::select(-probe_id) %>% #delete probe_id column

stats::aggregate(. ~ symbol, data =., FUN = fun) %>% #for multiple probes corresponding to the same symbol, take mean of these probes

tibble::column_to_rownames("symbol")#symbol column becomes row names

return(expr_annotated)#return this expr_annotated, then assign to annotate_expr

}

## Chip annotation

GSE28750_anno <- annotate_expr(GSE28750_expr_n, p2s)

head(GSE28750_anno)[, 1:3]

## save data

save(GSE28750_expr_n, GSE28750_targets, GSE28750_anno,

file = ".\\GSE28750_processed.Rda")

### Differential analysis DEGs

library(limma)

## for design

design <- model.matrix(~ group, data = GSE28750_targets)

colnames(design)

colnames(design) <- c("Control", "SepsisvsControl")

head(design)

# fit <- lmFit(expr, design)

# fit <- eBayes(fit)#Bayesian

# ## DEG results

# results <- summary(decideTests(fit, lfc = 0.5))

# results

## arrayWeights

expr <- GSE28750_anno

aw <- arrayWeights(expr, design = design)

aw

barplot(aw)

# fit <- lmFit(expr_anno, design)#linear fitting

fit <- lmFit(expr, design, weights = aw)

fit <- eBayes(fit)#Bayesian

## DEG results

results <- summary(decideTests(fit, lfc = 0.5))

results

# number = Inf: all differential analysis results; SepsisvsControl: represents the type to be compared

res_DEG <- topTable(fit, coef = "SepsisvsControl", number = Inf)

# res_DEG_sig <- topTable(fit, coef = "SepsisvsControl", n = Inf, p.value = 0.05, lfc = 1)#here p is adjusted p

res_DEG_sig <- topTable(fit, coef = "SepsisvsControl", n = Inf, p.value = 0.05, lfc = 1.2)

GSE28750_DEG1 <- topTable(fit, coef = 2, n = Inf)

identical(res_DEG, GSE28750_DEG1)

GSE28750_DEG <- topTable(fit, coef = 2, n = Inf) %>% # coef = 1 when there's no contrast.matrix()

rownames_to_column(var = "symbol")

# Filter results with |logFC|>0.5 and p<0.05 significant res

GSE28750_DEG_p <- GSE28750_DEG %>%

dplyr::filter(abs(logFC) > 1.2 , P.Value < 0.05)

### save data

save(GSE28750_DEG, GSE28750_DEG_p,

file = ".\\GSE28750_DEG.Rda")

### Extract genes for analysis ----

rm(list = ls())

load(".\\Validated dataset\\GSE28750\\GSE28750_DEG.Rda")

load(".\\Validated dataset\\GSE28750\\GSE28750_processed.Rda")

### Extract key gene expression

# df <- c("GPR84","HK3","MCEMP1","S100A12",'CLEC5A',"CLEC4D")

df <- c("GPR84","HK3","S100A12",'CLEC5A',"CLEC4D")

library(tidyverse)

GSE28750_keygene <- GSE28750_anno %>% rownames_to_column(var = "symbol")

GSE28750_keygene <- GSE28750_keygene %>%

filter(GSE28750_keygene$symbol %in% df ) %>%

column_to_rownames("symbol")

# GSE28750_keygene_1 <- GSE28750_anno %>% rownames_to_column(var = "symbol")

# GSE28750_keygene_1 <- GSE28750_keygene_1 %>% filter(GSE28750_keygene_1$symbol %in% df)

# identical(GSE28750_keygene,GSE28750_keygene_1)

### Extract key gene differential statistics

GSE28750_DEG_keygene <- GSE28750_DEG %>%

filter(GSE28750_DEG$symbol %in% df) %>% column_to_rownames("symbol")

identical(colnames(GSE28750_keygene),GSE28750_targets$sample_id)

### Save data

write.table(GSE28750_keygene, file = ".\\Validated dataset\\GSE28750\\GSE28750_keygene.txt",

sep = "\t",row.names = T,col.names = T,quote = F)

write.table(GSE28750_DEG_keygene, file = ".\\Validated dataset\\GSE28750\\GSE28750_DEG_keygene.txt",

sep = "\t",row.names = T,col.names = T,quote = F)

### Draw grouped violin plots ###

## Data organization

library(tidyverse)

GSE28750_keygene_t <- t(GSE28750_keygene) %>% as.data.frame() %>% rownames_to_column("sample_id")

GSE28750_targets_extract <- GSE28750_targets %>% dplyr::select(1,4)

identical(GSE28750_keygene_t$sample_id,GSE28750_targets_extract$sample_id)

GSE28750_keygene_merge <- inner_join(GSE28750_keygene_t,GSE28750_targets_extract,by="sample_id")

GSE28750_violin_data <- gather(GSE28750_keygene_merge,

category, value,-c(group,sample_id)) #wide to long format conversion code: 2119

# library(reshape2)

# GSE28750_keygene_merge$group <- as.factor(GSE28750_keygene_merge$group)

# GSE28750_violin_data_1 <- reshape2::melt(GSE28750_keygene_merge, id.vars=c('sample_id','group'),

# variable.name='gene',

# measure.vars=c("GPR84","HK3","MCEMP1","S100A12",'CLEC5A',"CLEC4D"),

# value.name='value')

# # id.vars: identifier variables (remain on columns, unchanged)

# # measure.vars: variables to be melted into one column

# # variable.name: name for the new column, defaults to "variable" if not specified

# # value.name: name for the column containing the values

fix(GSE28750_violin_data)

# See https://www.jianshu.com/p/8c3f74b55154; https://zhuanlan.zhihu.com/p/524095571;

pdf('F:\\Decoding Helix Courses\\GEOdatasets_sepsis1\\DEG_Sepsis\\Validated datasets\\GSE28750\\figures\\GSE28750_vio.pdf',10,5)

ggplot(GSE28750_violin_data, aes(x=category, y=value, fill=group))+

geom_violin(position = position_dodge(width=0.8),

# trim = FALSE,

scale = 'area')+

geom_boxplot(position = position_dodge(width = 0.8),

outlier.size = 0, width = 0.1, show.legend = FALSE)+

scale_fill_manual(values = c('Sepsis'='orange','Control'='blue'))+

# geom_text(inherit.aes = F,data = FDR_text_table, aes(x=gene, y=height, label=annotations), size=2.5, fontface='bold')+

# geom_segment(inherit.aes = F,data=segment_table,aes(x=xsl,xend=xel,y=ysl,yend=yel))+

# geom_segment(inherit.aes = F,data=segment_table,aes(x=xsr,xend=xer,y=ysr,yend=yer))+

# geom_segment(inherit.aes = F,data=segment_table,aes(x=xsh,xend=xeh,y=ysh,yend=yeh))+

theme_bw()+theme(panel.grid = element_line(colour = 'white'), plot.title = element_text(hjust = 0.5))+

ylab('normalized_value')+xlab('genes')+ylim(3,18)+

ggtitle('Violin plot of multiple genes')

dev.off()

GSE28750_vio <- ggplot(GSE28750_violin_data, aes(x=category, y=value, fill=group))+

geom_violin(position = position_dodge(width=0.8),

# trim = FALSE,

scale = 'area')+

geom_boxplot(position = position_dodge(width =0.8),

outlier.size = 0, width = 0.1, show.legend = FALSE)+

scale_fill_manual(values = c('Sepsis'='orange','Control'='blue'))+

# geom_text(inherit.aes = F,data = FDR_text_table, aes(x=gene, y=height, label=annotations), size=2.5, fontface='bold')+

# geom_segment(inherit.aes = F,data=segment_table,aes(x=xsl,xend=xel,y=ysl,yend=yel))+

# geom_segment(inherit.aes = F,data=segment_table,aes(x=xsr,xend=xer,y=ysr,yend=yer))+

# geom_segment(inherit.aes = F,data=segment_table,aes(x=xsh,xend=xeh,y=ysh,yend=yeh))+

theme_bw()+theme(panel.grid = element_line(colour = 'white'), plot.title = element_text(hjust = 0.5))+

ylab('normalized_value')+xlab('genes')+ylim(3,18)+

ggtitle('Violin plot of multiple genes')

GSE28750_vio

dev.off()

#### 13 ceRNA ----

##########################################

# See F:\\Decoding Helix Courses\\GEOdatasets_sepsis1\\DEG_Sepsis\\starbase_prediction.R for miRNA-lncRNA and lncRNA-miRNA sections

##########################################

# #Read original lncRNA and miRNA interaction data

# mir_lnc=read.table("starbase/Human/lncRNA_miRNA_interaction.txt", sep="\t", stringsAsFactors=F, header=T)

# #Extract only miRNAname and geneName columns and remove duplicate rows

# mir_lnc=unique(mir_lnc[,c("miRNAname","geneName")])

# #View first six rows

# head(mir_lnc)

# #Convert dataframe to list with lncRNA names as list names

# lnc_mir_list=unstack(mir_lnc,miRNAname~geneName)

# #View first six items in the list

# head(lnc_mir_list)

# #Convert dataframe to list with miRNA names as list names

# mir_lnc_list=unstack(mir_lnc,geneName~miRNAname)

# #View first six items in the list

# head(mir_lnc_list)

# #Set random seed for reproducibility (commented out)

# # set.seed(123)

# # #Randomly select 100 miRNAs to simulate candidate miRNAs

# # mir_candidate2=sample(names(mir_lnc_list),100)

# mir_candidate2 <- c("hsa-miR-4505",

# "hsa-miR-574-5p",

# "hsa-miR-4710",

# "hsa-miR-5589-5p",

# "hsa-miR-5004-5p",

# "hsa-miR-765",

# "hsa-miR-3189-3p",

# "hsa-miR-4534",

# "hsa-miR-4306",

# "hsa-miR-185-5p",

# "hsa-miR-4729",

# "hsa-miR-5696",

# "hsa-miR-6835-3p",

# "hsa-miR-4644",

# "hsa-miR-548e-5p",

# "hsa-miR-6780a-5p",

# "hsa-miR-8085",

# "hsa-miR-4446-3p")

# #Get subset of the list to get miRNA-lncRNA interactions, then convert to dataframe

# mir_target2=stack(mir_lnc_list[mir_candidate2])

# #Rename dataframe columns

# names(mir_target2)=c("lncRNA","miRNA")

# table(mir_target2$miRNA)

# write.table(mir_target2, file = ".\\STRING\\mir_target2.txt",

# sep = "\t",row.names = F,col.names = T,quote = F)

#### Extract lincRNAs corresponding to miRNAs commonly predicted by three databases for E2F1 ----

rm(list = ls())

#Read original lncRNA and miRNA interaction data

mir_lnc=read.table("starbase/Human/lncRNA_miRNA_interaction.txt", sep="\t", stringsAsFactors=F, header=T)

#Extract only miRNAname and geneName columns and remove duplicate rows

mir_lnc=unique(mir_lnc[,c("miRNAname","geneName")])

# These two steps are equivalent to the above step

# mir_lnc_1 <- mir_lnc[,c("miRNAname","geneName")]

# mir_lnc_2 <- unique(mir_lnc_1)

#View first six rows

head(mir_lnc)

#Convert dataframe to list with lncRNA names as list names

lnc_mir_list=unstack(mir_lnc,miRNAname~geneName)

head(lnc_mir_list) #View first six items in the list

#Convert dataframe to list with miRNA names as list names

mir_lnc_list=unstack(mir_lnc,geneName~miRNAname)

head(mir_lnc_list) #View first six items in the list

#Get miRNAs commonly predicted by three databases from venn website analysis tool

mir_candidate1 <- read.table("F:/Decoding Helix Courses/GEOdatasets_sepsis1/DEG_Sepsis/STRING/E2F1/venn_E2F1_miRNA_3com.txt",

sep="\t", stringsAsFactors=F, header=F)

mir_candidate2 <- mir_candidate1$V1

#Get subset of the list to get miRNA-lncRNA interactions, then convert to dataframe

mir_target2=stack(mir_lnc_list[mir_candidate2])

#Rename dataframe columns

names(mir_target2)=c("lncRNA","miRNA")

table(mir_target2$miRNA)

a <- table(mir_target2$"lncRNA")

# b <- table(mir_target2$lncRNA)

# identical(a,b)

library(tidyverse)

lncRNA_rank <- a %>% as.data.frame() %>% arrange(.,desc(Freq))

# b <- table(com_lncRNA_miRNA_dup_LINC$geneName) %>% as.data.frame() %>% arrange(.,desc(Freq))

write.table(lncRNA_rank, file = ".\\STRING\\E2F1\\lncRNA_rank_E2F1.txt",

sep = "\t",row.names = F,col.names = T,quote = F)

### Extract top 15 lncRNAs with most miRNA connections

lncRNA_15 <- read.table("F:/Decoding Helix Courses/GEOdatasets_sepsis1/DEG_Sepsis/STRING/E2F1/lncRNA_rank_E2F1(15).txt",

sep="\t", stringsAsFactors=F, header=F)

mir_target3 <- mir_target2[mir_target2$lncRNA %in% lncRNA_15$V1,]

write.table(mir_target3, file = ".\\STRING\\E2F1\\lncRNA(15)_mirRNA.txt",

sep = "\t",row.names = F,col.names = T,quote = F)

##### 14 Correlation ----

#### 1. Correlation between core genes ----

keygene <- read.table(".\\Validated datasets\\GSE28750\\GSE28750_keygene.txt",sep="\t",header=T,check.names=F,row.names = 1) #Read gene expression file

fix(keygene) #View data

keygene=t(keygene) #Transpose the data

keygene <- keygene[ ,-2]#Remove CLEC5A

keygene_cor <- cor(keygene) #Calculate correlation analysis

library(corrplot)

pdf('./Correlation/keygene_four/GSE28750_keygene_cor.pdf',width = 8,height = 8)

col3 <- colorRampPalette(c("#6D9EC1", "white", "#E46726"))

# See: F:/Decoding Helix Courses/Complete Code Breakthrough Bioinformatics Data Mining/Bioinformatics System Course Lower Section Code/Level 3/L71_Codes/3.phenotype/cell cycle/cell_cycle.R code: 74

# col3 <- colorRampPalette(c("blue", "white", "red")) #Custom gradient colors corrgram can also specify See "R Language Practical" First Edition P259

# See: F:/Decoding Helix Courses/Complete Code Breakthrough Bioinformatics Data Mining/Bioinformatics System Course Lower Section Code/Level 3/L71_Codes/4.cross/cross/cross.R code: 27~34

# color1 <- colorRampPalette(c("blue","red"))

# corrplot(cor,

# method = "circle",

# order = "hclust", #Clustering

# addCoef.col = "black",

# addrect = 2, #Number of rectangles drawn on the graph according to hierarchical clustering, only valid when order = "hclust"

# col = color1(100))#Add colors, 100 indicates uniformity of gradient colors, smaller values make boundaries clearer

# dev.off()

cor.plot <- corrplot(corr = keygene_cor,

col=col3(10),

type="upper",

tl.pos="d", #tl.pos=”d” specifies text label (variable name) position on the diagonal

#tl.pos=”n” #means no text labels

# col="black",

tl.cex = 0.6) #Plot upper right Method defaults to "circle"

#tl.cex = 0.75 ＃Text label (variable name) size, default is 0.75 times

cor.plot <- corrplot(corr = keygene_cor,

add=TRUE,

type="lower",

# col=col3(10), #col=col3(10) #Color specified gradient colors 10 gradient levels

# method="color",

method="number",

col="black",

# addCoef.col="black",

diag=FALSE,

tl.pos="n",

cl.pos="n",

number.cex = 0.7) #Plot lower left Method "color" #Size of correlation coefficient text in the graph, default is 0.7 times

dev.off()

#### 2. Correlation between genes and immune cells ----

rm(list=ls())

library(e1071)

library(parallel)

library(preprocessCore)

# if(!dir.exists("CIBERSORT")) dir.create("CIBERSORT")

setwd("F:/Decoding Helix Courses/GEOdatasets_sepsis1/DEG_Sepsis/Correlation")

source("./CIBERSORT.R")

if(!dir.exists("CIBERSORT_GSE28750"))dir.create("CIBERSORT_GSE28750")

#Assign "LM22.txt" to sig_matrix, then the program will automatically read this file in this directory

sig_matrix <- "LM22.txt" # "LM22.txt" is the annotation file for 22 immune cells

load("./GSE28750_processed.Rda")

library(tidyverse)

GSE28750_anno <- GSE28750_anno %>% rownames_to_column("Gene")

write.table(GSE28750_anno, file = "GSE28750_anno.txt",sep="\t", quote=F, col.names=T,row.names = F)

mixture_file = 'GSE28750_anno.txt' # GSE137340 dataset expression data (note to adjust the first row of the table)

# GSE28750_anno <- GSE28750_anno %>% rownames_to_column("Gene")

# #If the above step is not done, the first row will shift left when saving. So adjust the format after saving.

# write.table(GSE28750_anno, file = "GSE28750_anno.txt",sep="\t", quote=F, col.names=T,row.names = T)

# mixture_file = 'GSE137340_anno.txt' # GSE137340 dataset expression data (note to adjust the first row of the table)

# perm indicates number of permutations, more times means more stable results

res_cibersort <- CIBERSORT(sig_matrix,

mixture_file,

perm=100, #perm, number of permutation tests, default is 0, >100 can calculate significance p-values

QN=TRUE) #QN, whether to standardize the input expression matrix

#Equivalent to calculating 100 times continuously to see if the results are random

# Create a normalization function

normalize <- function(x){# (x-min(x))/(X's max-x's min)

return((x-min(x))/(max(x)-min(x)))}

# Use normalize to standardize CIBERSORT scores

CIBERSORT_Score <- res_cibersort

CIBERSORT_Score_GSE28750 <- normalize(CIBERSORT_Score)

save(CIBERSORT_Score_GSE28750, CIBERSORT_Score,

file = "./CIBERSORT_GSE28750/CIBERSORT_Score_GSE28750.Rdata") #Save intermediate files

load("./CIBERSORT_GSE28750/CIBERSORT_Score_GSE28750.Rdata")

CIBERSORT_Score_GSE28750 <- CIBERSORT_Score_GSE28750[,1:22]

#colSums sums columns

ciber.res <- CIBERSORT_Score_GSE28750[,colSums(CIBERSORT_Score_GSE28750) > 0] #Remove cells with all zero abundance

### Core gene expression levels

keygene <- read.table("F:\\Decoding Helix Courses\\GEOdatasets_sepsis1\\DEG_Sepsis\\Validated datasets\\GSE28750\\GSE28750_keygene.txt",

sep="\t",header=T,check.names=F,row.names = 1) #Read gene expression file

# fix(keygene) #View data

keygene=t(keygene) #Transpose the data

keygene <- keygene[ ,-2] #Remove CLEC5A

category, value,-c(group,sample_id)) # wide to long format conversion code: 2119

# library(reshape2)

# GSE28750_keygene_merge$group <- as.factor(GSE28750_keygene_merge$group)

# GSE28750_violin_data_1 <- reshape2::melt(GSE28750_keygene_merge, id.vars=c('sample_id','group'),

# variable.name='gene',

# measure.vars=c("GPR84","HK3","MCEMP1","S100A12",'CLEC5A',"CLEC4D"),

# value.name='value')

# # id.vars: identifier variables (remain on columns, unchanged)

# # measure.vars: variables to be melted into one column

# # variable.name: name for the new column, defaults to "variable" if not specified

# # value.name: name for the column containing the values

fix(GSE28750_violin_data)

# See https://www.jianshu.com/p/8c3f74b55154; https://zhuanlan.zhihu.com/p/524095571;

pdf('F:\\Decoding Helix Courses\\GEOdatasets_sepsis1\\DEG_Sepsis\\Validated datasets\\GSE28750\\figures\\GSE28750_vio.pdf',10,5)

ggplot(GSE28750_violin_data, aes(x=category, y=value, fill=group))+

geom_violin(position = position_dodge(width=0.8),

# trim = FALSE,

scale = 'area')+

geom_boxplot(position = position_dodge(width = 0.8),

outlier.size = 0, width = 0.1, show.legend = FALSE)+

scale_fill_manual(values = c('Sepsis'='orange','Control'='blue'))+

# geom_text(inherit.aes = F,data = FDR_text_table, aes(x=gene, y=height, label=annotations), size=2.5, fontface='bold')+

# geom_segment(inherit.aes = F,data=segment_table,aes(x=xsl,xend=xel,y=ysl,yend=yel))+

# geom_segment(inherit.aes = F,data=segment_table,aes(x=xsr,xend=xer,y=ysr,yend=yer))+

# geom_segment(inherit.aes = F,data=segment_table,aes(x=xsh,xend=xeh,y=ysh,yend=yeh))+

theme_bw()+theme(panel.grid = element_line(colour = 'white'), plot.title = element_text(hjust = 0.5))+

ylab('normalized_value')+xlab('genes')+ylim(3,18)+

ggtitle('Violin plot of multiple genes')

dev.off()

GSE28750_vio <- ggplot(GSE28750_violin_data, aes(x=category, y=value, fill=group))+

geom_violin(position = position_dodge(width=0.8),

# trim = FALSE,

scale = 'area')+

geom_boxplot(position = position_dodge(width =0.8),

outlier.size = 0, width = 0.1, show.legend = FALSE)+

scale_fill_manual(values = c('Sepsis'='orange','Control'='blue'))+

# geom_text(inherit.aes = F,data = FDR_text_table, aes(x=gene, y=height, label=annotations), size=2.5, fontface='bold')+

# geom_segment(inherit.aes = F,data=segment_table,aes(x=xsl,xend=xel,y=ysl,yend=yel))+

# geom_segment(inherit.aes = F,data=segment_table,aes(x=xsr,xend=xer,y=ysr,yend=yer))+

# geom_segment(inherit.aes = F,data=segment_table,aes(x=xsh,xend=xeh,y=ysh,yend=yeh))+

theme_bw()+theme(panel.grid = element_line(colour = 'white'), plot.title = element_text(hjust = 0.5))+

ylab('normalized_value')+xlab('genes')+ylim(3,18)+

ggtitle('Violin plot of multiple genes')

GSE28750_vio

dev.off()

#### 15 ROC Analysis ----

rm(list = ls())

library(tidyverse)

setwd("F:/Decoding Helix Courses/GEOdatasets_spsis1/DEG_SPSIS")

if(!dir.exists("ROC")) dir.create("ROC")

setwd("F:/Decoding Helix Courses/GEOdatasets_spsis1/DEG_SPSIS/ROC")

# load("F:\\Decoding Helix Courses\\GEOdatasets_spsis1\\DEG_SPSIS\\Validated dataset\\GSE28750\\GSE28750_DEG.Rda")

load("F:\\Decoding Helix Courses\\GEOdatasets_spsis1\\DEG_SPSIS\\GSE54514\\GSE54514_processed.Rda")

## Extract key genes for analysis:

GetFactors <- c("GPR84","HK3","MCEMP1","S100A12",'CLEC5A',"CLEC4D")

# GetFactors <- c("GPR84","HK3","S100A12",'CLEC5A',"CLEC4D")

## Merge key gene expression files

GSE54514_targets_sur <- GSE54514_targets %>%

mutate(sur=(ifelse(str_detect(sample_name, "survivor"),

ifelse(str_detect(sample_name, "non"),"nonsurvivor","survivor"),"normal")))

# GSE54514_targets_sur_factor <- GSE54514_targets_sur %>% rownames_to_column("sample") %>%

# mutate(fustatus=factor(ifelse(GSE54514_targets_sur$sur == "nonsurvivor","1","0"),

# levels=c('1','0')))

# Notes on converting character to factor type:

# Character type needs to be converted to numeric first before converting to factor

# Or convert to character factor type; e.g.: level(normal,tumor)

GSE54514_targets_sur_factor <- GSE54514_targets_sur %>% rownames_to_column("sample") %>%

mutate(fustatus=ifelse(GSE54514_targets_sur$sur == "nonsurvivor","1","0"))

GSE54514_targets_sur_factor$fustatus <- as.numeric(GSE54514_targets_sur_factor$fustatus)

GSE54514_targets_sur_factor$fustatus <- as.factor(GSE54514_targets_sur_factor$fustatus)

GSE54514_anno_GetFactors <- GSE54514_anno[GetFactors,]

identical(GSE54514_targets_sur_factor$sample,colnames(GSE54514_anno_GetFactors))

GSE54514_anno_GetFactors <- GSE54514_anno_GetFactors %>% t() %>% as.data.frame() %>% rownames_to_column("sample")

GSE54514_anno_GetFactors_factor <- merge(GSE54514_anno_GetFactors,GSE54514_targets_sur_factor,by="sample")

GSE54514_anno_GetFactors_factor <- GSE54514_anno_GetFactors_factor %>% column_to_rownames("sample")

# Univariate analysis:

GetFactors1 <- c("GPR84","HK3","S100A12",'CLEC5A',"CLEC4D")

XNames <- colnames(GSE54514_anno_GetFactors_factor)[c(1,2,4,5,6)] ## N_category is in the seventh row

YNames <- colnames(GSE54514_anno_GetFactors_factor)[13]

df_train_all <- GSE54514_anno_GetFactors_factor

df_train <- df_train_all[!df_train_all$group=="Control",]

# df_train <- df_train_all

save(df_train , df_train_all,

file = ".\\df_train.Rda")

source('../Lesson1_Function/UniLog.R', encoding = "utf-8") ## Load R function

## We created a function below that can obtain univariate analysis results. Note: This uses an object-oriented approach.

UniVar <- lapply(XNames,function(x){

UniLog(df = df_train,

x = x,

y = YNames)

})

library(plyr)

UniVar <- ldply(UniVar,data.frame) ## ldply is a function that performs the same operation on multiple objects, mainly for convenience.

UniVar

# Multivariate analysis:

# Here we decide to include P < 0.2 based on the univariate results of the simulated data into the multivariate model.

GetFactors <- UniVar$characteristics[which(UniVar$Pval < 0.2)] %>% as.character()

GetFactors

# save(GetFactors,file = './Lesson1_Results/Tmp_GetFactors.RData') ## Save results as RData file for future loading.

## Logistic regression for multivariate data ##

# fml: is the formula

fml <- as.formula(paste('fustatus', ## Outcome variable Y

paste(GetFactors, collapse='+'), sep=" ~ ")) ##

# glm: is the logistic regression function

mod.full <- glm(fml, ## Key glm function

data = df_train, ## Training set name

family = binomial()) ## Logistic regression method

summary(mod.full) # View results

## Stepwise regression for multivariate data ## Stepwise regression considers multicollinearity

library(MASS)

# Create a null model first

mod.none <- glm(fustatus~1, # First create a null model

data = df_train,

family = binomial())

mod.full <- glm(fml,

data = df_train,

family = binomial())

stepAIC(mod.full) ## Method I: AIC, the smaller the better

# N_category ~ T_category + HPV_status

#

# Df Deviance AIC

# <none> 213.89 219.89

# - HPV_status 1 226.14 230.14 Removing HPV increases AIC from 219 to 230, so we shouldn't remove HPV

# - T_category 1 229.09 233.09

mod_step <- step(mod.none,scope = formula(mod.full),direction = 'forward',trace = F)

MultiSum <- summary(mod_step)

MultiSum

## Table

MultiName <- as.character(UniVar$characteristics[UniVar$Pval < 0.2])

tmp_Multi <- MultiSum$coefficients %>% data.frame()

MR <- tmp_Multi[MultiName, ]$Estimate %>% round(.,2)

MP <- tmp_Multi[MultiName, ]$Pr...z.. %>% round(.,4)

MulLog <- data.frame('characteristics' = MultiName,

'Rval' = MR,

'Pval' = MP)

Final_Results <- merge.data.frame(UniVar,MulLog, by = "characteristics", all = T, sort = T) # Merge univariate and multivariate

Final_Results

View(Final_Results)

setwd("F:/Decoding Helix Courses/GEOdatasets_spsis1/DEG_SPSIS/ROC")

write.table(Final_Results, file = "Final_Results.txt",sep = "\t",row.names = F,col.names = T,quote = F)

## Build Logistic regression formula ####

## This code (explaining univariate and multivariate logistic regression) is the same as

## (univariate and multivariate analysis where the dependent variable is binary distributed)

rm(list = ls())

setwd("F:/Decoding Helix Courses/GEOdatasets_spsis1/DEG_SPSIS/ROC")

load("./df_train.Rda")

df_train_all <- df_train_all[ ,-3]

df_train <- df_train[ ,-3]

# GetFactors <- c("GPR84","HK3","S100A12",'CLEC5A',"CLEC4D","CLEC5A")

# GetFactors <- c("S100A12")

# GetFactors <- c("HK3")

# GetFactors <- c("CLEC4D")

# GetFactors <- c("GPR84")

GetFactors <- c("CLEC5A")

fml <- as.formula(paste('fustatus', ## Outcome variable Y in the formula

'~', ## Tilde symbol

paste(GetFactors,collapse = '+'),collapse = ' ')) ## Predictors X connected by "+" First use + then ~

# fml_1 <- as.formula(fustatus~CLEC5A)

# identical(fml,fml_1)

library(rms)

# df_train <- df_train_all

ddist <- datadist(df_train) ## Environment variable setting, necessary for later nomogram creation

options(datadist = 'ddist')

#### Step I Logistic regression ###

## Method I ##

# lrm(): simple linear regression; glm is more powerful than lm, can do discrete outcome models, etc.;

# glm is the generalized linear regression function, of course you can also use it for linear regression

# lm is the linear regression function, cannot fit generalized linear regression models

mod.bi <- lrm(fml, ## Input logistic regression formula fml lrm——Logistic Regression Model

data = df_train) ## Define dataset

#summary(mod.bi)

mod.bi# View regression coefficients Coef and p-values

## ROC

# # The following two codes are for multivariate indicators

# fml <- as.formula(paste('fustatus', ## Outcome variable Y in the formula

# '~', ## Tilde symbol

# paste(GetFactors,collapse = '+'),collapse = ' ')) ## Predictors X connected by "+"

# fit2 <- glm(fml,x=T,y=T,data = df_train, family = "binomial") ## Establish logistic regression model

library("pROC") ## Load package

mycol <- rainbow(10) ## Define some colors, you can set your own

# pdf(paste0('Try_log','_Plot5_ROC.pdf'),height=12,width=12)

auc.out <- c()

x <- plot.roc(df_train$fustatus , # y-value, i.e. outcome variable

# fit2$fitted.values,# Different from univariate ROC

# df_train$GPR84,# x-value, i.e. whether drinking

# df_train$HK3,

df_train$CLEC5A,

# df_train$CLEC5A,

# df_train$S100A12,

ylim=c(0,1), # Y-axis range

xlim=c(1,0), # X-axis range

# smooth=T, # Draw smooth curve

ci=TRUE, # Calculate 95%CI confidence interval

main='Receiver Operating Characteristic', # Define a graph name

# print.thres="best", # Write the threshold on the graph where sensitivity + specificity is maximized

col=mycol[2],# Line color

lwd=2, # Line thickness

legacy.axes=T, ## X-axis is "1-specificity", from 0 to 1

newpage = F) # Don't start a new page (for plotting)

ci.lower <- round(as.numeric(x$ci[1]),3) # Lower bound of confidence interval

ci.upper <- round(as.numeric(x$ci[3]),3) # Upper bound of confidence interval

auc.ci <- c('CLEC5A',round(as.numeric(x$auc),3),paste(ci.lower,ci.upper,sep="-")) # Format for outputting 95%CI

auc.out <- rbind(auc.out,auc.ci) # Combine together

### Output AUC, AUC CI to file

auc.out <- as.data.frame(auc.out)

colnames(auc.out) <- c('Factors',"AUC","AUC CI")

### Draw legend

legend.name <- paste('CLEC5A',"AUC",auc.out$AUC,sep=" ") # Legend we want to output

legend("bottomright", # Bottom right of the graph

legend=legend.name, # Output text

col = mycol[2:length(df)], # Color definition

lwd = 2, # Line thickness

bty="n",

cex = 0.8) # Don't draw border

dev.off()

#### 16 Core Gene Correlation Heatmap ####

#### 16.1 Correlation between core genes and immune/inflammatory genes ----

rm(list = ls())

library(tidyverse)

setwd("F:/Decoding Helix Courses/GEOdatasets_spsis1/DEG_SPSIS")

## Read expression files of immune and inflammatory genes

gene_immu <- read.csv(".\\Validated dataset\\GSE28750\\GeneCards-immune regulators.csv",

sep=",",header=T,check.names=F)

gene_imfla <- read.csv(".\\Validated dataset\\GSE28750\\GeneCards-Inflammatory factor.csv",

sep=",",header=T,check.names=F)

gene_inter <- intersect(gene_immu$"Gene Symbol",gene_imfla$"Gene Symbol")

## Sort gene_immu by Relevance score and extract genes with `Relevance score` > 20

gene_immu <- gene_immu %>% arrange("Relevance score")

gene <- gene_immu$`Gene Symbol`[which(gene_immu$`Relevance score` > 20)] %>% as.character()

load(".\\Validated dataset\\GSE28750\\GSE28750_DEG.Rda")

load(".\\Validated dataset\\GSE28750\\GSE28750_processed.Rda")

gene_com <- intersect(gene,GSE28750_DEG_p$symbol)

## Sort gene_imfla by Relevance score and extract genes with `Relevance score` > 20

gene_imfla <- gene_imfla %>% arrange("Relevance score")

gene1 <- gene_imfla$`Gene Symbol`[which(gene_imfla$`Relevance score` > 20)] %>% as.character()

gene1_com <- intersect(gene1,GSE28750_DEG_p$symbol)

com <- intersect(gene_com,gene1_com)

## Calculate correlation between core genes and immune/inflammatory genes:

# Extract expression of core genes in GSE28750

keygene <- read.table("F:\\Decoding Helix Courses\\GEOdatasets_spsis1\\DEG_SPSIS\\Validated dataset\\GSE28750\\GSE28750_keygene.txt",

sep="\t",header=T,check.names=F,row.names = 1) # Read gene expression file

keygene <- keygene[-2,]

# Extract expression of immune genes in GSE28750

GSE28750_anno <- GSE28750_anno %>% rownames_to_column("sample")

GSE28750_anno_immu <- GSE28750_anno %>% filter(GSE28750_anno$sample %in% gene_com)

# Extract expression of inflammatory genes in GSE28750

GSE28750_anno_imfla <- GSE28750_anno %>% filter(GSE28750_anno$sample %in% gene1_com)

## Data format conversion

keygene=t(keygene) # Transpose the data

GSE28750_anno_immu <- GSE28750_anno_immu %>% column_to_rownames("sample")

GSE28750_anno_immu <- t(GSE28750_anno_immu)

GSE28750_anno_imfla <- GSE28750_anno_imfla %>% column_to_rownames("sample")

GSE28750_anno_imfla <- t(GSE28750_anno_imfla)

identical(rownames(GSE28750_anno_immu),rownames(GSE28750_anno_imfla))

identical(rownames(GSE28750_anno_immu),rownames(keygene))

## Calculate correlation using corr.test()

library(psych)

# See webpage: https://www.jianshu.com/p/d86ddf8fd48f

cor_t <- corr.test(keygene, GSE28750_anno_immu, method = "pearson",adjust="none")

## Extract correlation coefficients and p-values

cor_t_r <- cor_t$r

cor_t_p <- cor_t$p

# As above, cor_t_r == cor == merge_cor_1

# But cor_t_p is not equal to cor_res_2

cmt <- cor_t_r

# cmt <- cmt %>% as.data.frame() %>% arrange(CLEC4D) # arrange: means ascending order

cmt <- t(cmt)

# cmt <- cmt %>% as.data.frame() %>% arrange(desc(Clec4D)) # After adding desc, it means descending order

cmt <- cmt %>% as.data.frame() %>% arrange(Clec4D)

cmt <- t(cmt)

pmt <- cor_t_p[ ,colnames(cmt)]

identical(colnames(pmt),colnames(cmt))

pmt_1 <- cor_t_p

# Determine significance

if (!is.null(pmt)){

ssmt <- pmt< 0.01

pmt[ssmt] <-'**'

smt <- pmt >0.01& pmt <0.05

pmt[smt] <- '*'

pmt[!ssmt&!smt]<- ''

} else {

pmt <- F

}

# Visualization

# mycol <- ggplot2::alpha(rainbow(ncol(cor_t_r)), 0.7) # Create color palette (70% transparency)

# col3 <- colorRampPalette(c("blue", "white", "red")) # Custom gradient colors, corrgram can also specify, see "R Language Practical" First Edition P259

library(pheatmap)

pdf("./Correlation/keygene_four/keygene_immu.PDF", 15, 6)

col3 <- colorRampPalette(c("#6D9EC1", "white", "#E46726"))

pheatmap(cmt,scale = "none",

cluster_row = F, cluster

library(pheatmap)

pdf("./Correlation/keygene_four/keygene_immu.PDF", width = 15, height = 6)

col3 <- colorRampPalette(c("#6D9EC1", "white", "#E46726"))

pheatmap(cmt, scale = "none",

cluster_row = FALSE, cluster_col = FALSE,

border = NA,

display_numbers = pmt, fontsize_number = 12, number_color = "white",

color = col3(10),

cellwidth = 20, cellheight = 20)

dev.off()

## Calculate correlation using corr.test()

library(psych)

# See webpage: https://www.jianshu.com/p/d86ddf8fd48f

cor_t <- corr.test(keygene, GSE28750_anno_imfla, method = "pearson", adjust = "none")

## Extract correlation coefficients and p-values

cor_t_r <- cor_t$r

cor_t_p <- cor_t$p

# As shown above, cor_t_r == cor == merge_cor_1

# But cor_t_p is not equal to cor_res_2

cmt <- cor_t_r

# cmt <- cmt %>% as.data.frame() %>% arrange(Clec4D) # arrange: means ascending order

cmt <- t(cmt)

cmt <- cmt %>% as.data.frame() %>% arrange(Clec4D) # After adding desc, it means descending order

cmt <- t(cmt)

pmt <- cor_t_p[, colnames(cmt)]

# Determine significance

if (!is.null(pmt)) {

ssmt <- pmt < 0.01

pmt[ssmt] <- '**'

smt <- pmt > 0.01 & pmt < 0.05

pmt[smt] <- '*'

pmt[!ssmt & !smt] <- ''

} else {

pmt <- FALSE

}

# Visualization

# mycol <- ggplot2::alpha(rainbow(ncol(cor_t_r)), 0.7) # Create color palette (70% transparency)

# col3 <- colorRampPalette(c("blue", "white", "red")) # Custom gradient colors, corrgram can also specify, see "R Language Practical" First Edition P259

library(pheatmap)

pdf("./Correlation/keygene_four/keygene_imfla.PDF", width = 15, height = 6)

col3 <- colorRampPalette(c("#6D9EC1", "white", "#E46726"))

pheatmap(cmt, scale = "none",

cluster_row = FALSE, cluster_col = FALSE,

border = NA,

display_numbers = pmt, fontsize_number = 12, number_color = "white",

color = col3(10),

cellwidth = 20, cellheight = 20)

dev.off()

#### 16.2 Correlation between core genes and metabolic genes ----

rm(list = ls())

library(tidyverse)

setwd("F:/Decoding Helix Courses/GEOdatasets_spsis1/DEG_SPSIS")

## Read metabolic gene expression file

gene_meta <- read.csv(".\\Validated dataset\\GSE28750\\GeneCards-metabolic_genes.csv",

sep = ",", header = TRUE, check.names = FALSE)

## Sort gene_immu by Relevance score and extract genes with `Relevance score` > 20

# Note: This seems to be using gene_meta instead of gene_immu

gene_meta_1 <- gene_meta %>% arrange("Relevance score")

gene_meta_2 <- gene_meta %>% arrange(desc(gene_meta$`Relevance score`))

identical(gene_meta, gene_meta_1)

identical(gene_meta_1, gene_meta_2)

gene <- gene_meta$`Gene Symbol`[which(gene_meta$`Relevance score` > 20)] %>% as.character()

load(".\\Validated dataset\\GSE28750\\GSE28750_DEG.Rda")

load(".\\Validated dataset\\GSE28750\\GSE28750_processed.Rda")

gene_com <- intersect(gene, GSE28750_DEG_p$symbol)

## Calculate correlation between core genes and metabolic genes:

# Extract expression of core genes in GSE28750

keygene <- read.table("F:\\Decoding Helix Courses\\GEOdatasets_spsis1\\DEG_SPSIS\\Validated dataset\\GSE28750\\GSE28750_keygene.txt",

sep = "\t", header = TRUE, check.names = FALSE, row.names = 1) # Read gene expression file

keygene <- keygene[-2, ]

# Extract expression of metabolic genes in GSE28750

GSE28750_anno <- GSE28750_anno %>% rownames_to_column("sample")

GSE28750_anno_meta <- GSE28750_anno %>% filter(GSE28750_anno$sample %in% gene_com)

## Data format conversion

keygene <- t(keygene) # Transpose the data

GSE28750_anno_meta <- GSE28750_anno_meta %>% column_to_rownames("sample")

GSE28750_anno_meta <- t(GSE28750_anno_meta)

identical(rownames(GSE28750_anno_meta), rownames(keygene))

## Calculate correlation using corr.test()

library(psych)

# See webpage: https://www.jianshu.com/p/d86ddf8fd48f

cor_t <- corr.test(keygene, GSE28750_anno_meta, method = "pearson", adjust = "none")

## Extract correlation coefficients and p-values

cor_t_r <- cor_t$r

cor_t_p <- cor_t$p

# As shown above, cor_t_r == cor == merge_cor_1

# But cor_t_p is not equal to cor_res_2

cmt <- cor_t_r

# cmt <- cmt %>% as.data.frame() %>% arrange(Clec4D) # arrange: means ascending order

cmt <- t(cmt)

# cmt <- cmt %>% as.data.frame() %>% arrange(desc(Clec4D)) # After adding desc, it means descending order

cmt <- cmt %>% as.data.frame() %>% arrange(Clec4D)

cmt <- t(cmt)

pmt <- cor_t_p[, colnames(cmt)]

identical(colnames(pmt), colnames(cmt))

pmt_1 <- cor_t_p

# Determine significance

if (!is.null(pmt)) {

ssmt <- pmt < 0.01

pmt[ssmt] <- '**'

smt <- pmt > 0.01 & pmt < 0.05

pmt[smt] <- '*'

pmt[!ssmt & !smt] <- ''

} else {

pmt <- FALSE

}

# Visualization

# mycol <- ggplot2::alpha(rainbow(ncol(cor_t_r)), 0.7) # Create color palette (70% transparency)

# col3 <- colorRampPalette(c("blue", "white", "red")) # Custom gradient colors, corrgram can also specify, see "R Language Practical" First Edition P259

library(pheatmap)

pdf("./Correlation/keygene_four/keygene_meta.PDF", width = 15, height = 6)

col3 <- colorRampPalette(c("#6D9EC1", "white", "#E46726"))

pheatmap(cmt, scale = "none",

cluster_row = FALSE, cluster_col = FALSE,

border = NA,

display_numbers = pmt, fontsize_number = 12, number_color = "white",

color = col3(10),

cellwidth = 20, cellheight = 20)

dev.off()

library(pheatmap)

pdf("./Correlation/keygene_four/keygene_immu.PDF", width = 15, height = 6)

col3 <- colorRampPalette(c("#6D9EC1", "white", "#E46726"))

pheatmap(cmt, scale = "none",

cluster_row = FALSE, cluster_col = FALSE,

border = NA,

display_numbers = pmt, fontsize_number = 12, number_color = "white",

color = col3(10),

cellwidth = 20, cellheight = 20)

dev.off()

## Calculate correlation using corr.test()

library(psych)

# See webpage: https://www.jianshu.com/p/d86ddf8fd48f

cor_t <- corr.test(keygene, GSE28750_anno_imfla, method = "pearson", adjust = "none")

## Extract correlation coefficients and p-values

cor_t_r <- cor_t$r

cor_t_p <- cor_t$p

# As shown above, cor_t_r == cor == merge_cor_1

# But cor_t_p is not equal to cor_res_2

cmt <- cor_t_r

# cmt <- cmt %>% as.data.frame() %>% arrange(Clec4D) # arrange: means ascending order

cmt <- t(cmt)

cmt <- cmt %>% as.data.frame() %>% arrange(Clec4D) # After adding desc, it means descending order

cmt <- t(cmt)

pmt <- cor_t_p[, colnames(cmt)]

# Determine significance

if (!is.null(pmt)) {

ssmt <- pmt < 0.01

pmt[ssmt] <- '**'

smt <- pmt > 0.01 & pmt < 0.05

pmt[smt] <- '*'

pmt[!ssmt & !smt] <- ''

} else {

pmt <- FALSE

}

# Visualization

# mycol <- ggplot2::alpha(rainbow(ncol(cor_t_r)), 0.7) # Create color palette (70% transparency)

# col3 <- colorRampPalette(c("blue", "white", "red")) # Custom gradient colors, corrgram can also specify, see "R Language Practical" First Edition P259

library(pheatmap)

pdf("./Correlation/keygene_four/keygene_imfla.PDF", width = 15, height = 6)

col3 <- colorRampPalette(c("#6D9EC1", "white", "#E46726"))

pheatmap(cmt, scale = "none",

cluster_row = FALSE, cluster_col = FALSE,

border = NA,

display_numbers = pmt, fontsize_number = 12, number_color = "white",

color = col3(10),

cellwidth = 20, cellheight = 20)

dev.off()

#### 16.2 Correlation between core genes and metabolic genes ----

rm(list = ls())

library(tidyverse)

setwd("F:/Decoding Helix Courses/GEOdatasets_spsis1/DEG_SPSIS")

## Read metabolic gene expression file

gene_meta <- read.csv(".\\Validated dataset\\GSE28750\\GeneCards-metabolic_genes.csv",

sep = ",", header = TRUE, check.names = FALSE)

## Sort gene_immu by Relevance score and extract genes with `Relevance score` > 20

# Note: This seems to be using gene_meta instead of gene_immu

gene_meta_1 <- gene_meta %>% arrange("Relevance score")

gene_meta_2 <- gene_meta %>% arrange(desc(gene_meta$`Relevance score`))

identical(gene_meta, gene_meta_1)

identical(gene_meta_1, gene_meta_2)

gene <- gene_meta$`Gene Symbol`[which(gene_meta$`Relevance score` > 20)] %>% as.character()

load(".\\Validated dataset\\GSE28750\\GSE28750_DEG.Rda")

load(".\\Validated dataset\\GSE28750\\GSE28750_processed.Rda")

gene_com <- intersect(gene, GSE28750_DEG_p$symbol)

## Calculate correlation between core genes and metabolic genes:

# Extract expression of core genes in GSE28750

keygene <- read.table("F:\\Decoding Helix Courses\\GEOdatasets_spsis1\\DEG_SPSIS\\Validated dataset\\GSE28750\\GSE28750_keygene.txt",

sep = "\t", header = TRUE, check.names = FALSE, row.names = 1) # Read gene expression file

keygene <- keygene[-2, ]

# Extract expression of metabolic genes in GSE28750

GSE28750_anno <- GSE28750_anno %>% rownames_to_column("sample")

GSE28750_anno_meta <- GSE28750_anno %>% filter(GSE28750_anno$sample %in% gene_com)

## Data format conversion

keygene <- t(keygene) # Transpose the data

GSE28750_anno_meta <- GSE28750_anno_meta %>% column_to_rownames("sample")

GSE28750_anno_meta <- t(GSE28750_anno_meta)

identical(rownames(GSE28750_anno_meta), rownames(keygene))

## Calculate correlation using corr.test()

library(psych)

# See webpage: https://www.jianshu.com/p/d86ddf8fd48f

cor_t <- corr.test(keygene, GSE28750_anno_meta, method = "pearson", adjust = "none")

## Extract correlation coefficients and p-values

cor_t_r <- cor_t$r

cor_t_p <- cor_t$p

# As shown above, cor_t_r == cor == merge_cor_1

# But cor_t_p is not equal to cor_res_2

cmt <- cor_t_r

# cmt <- cmt %>% as.data.frame() %>% arrange(Clec4D) # arrange: means ascending order

cmt <- t(cmt)

# cmt <- cmt %>% as.data.frame() %>% arrange(desc(Clec4D)) # After adding desc, it means descending order

cmt <- cmt %>% as.data.frame() %>% arrange(Clec4D)

cmt <- t(cmt)

pmt <- cor_t_p[, colnames(cmt)]

identical(colnames(pmt), colnames(cmt))

pmt_1 <- cor_t_p

# Determine significance

if (!is.null(pmt)) {

ssmt <- pmt < 0.01

pmt[ssmt] <- '**'

smt <- pmt > 0.01 & pmt < 0.05

pmt[smt] <- '*'

pmt[!ssmt & !smt] <- ''

} else {

pmt <- FALSE

}

# Visualization

# mycol <- ggplot2::alpha(rainbow(ncol(cor_t_r)), 0.7) # Create color palette (70% transparency)

# col3 <- colorRampPalette(c("blue", "white", "red")) # Custom gradient colors, corrgram can also specify, see "R Language Practical" First Edition P259

library(pheatmap)

pdf("./Correlation/keygene_four/keygene_meta.PDF", width = 15, height = 6)

col3 <- colorRampPalette(c("#6D9EC1", "white", "#E46726"))

pheatmap(cmt, scale = "none",

cluster_row = FALSE, cluster_col = FALSE,

border = NA,

display_numbers = pmt, fontsize_number = 12, number_color = "white",

color = col3(10),

cellwidth = 20, cellheight = 20)

dev.off()
