# Supplementary material for: Identification of hub genes and prediction of the ceRNA network in adult sepsis
Source: PeerJ. 2025 Aug 13;13:e19619. doi: 10.7717/peerj.19619 (PMC12357545; doi:10.7717/peerj.19619)
Supplement: Supplemental Information 13 [file peerj-13-19619-s013.docx]

| **S1 Table. Primers for RT-qPCR** | | |
| --- | --- | --- |
| Gene | Primer | Sequence (5’ -> 3’) |
| GPR84 (human) | Forward | GTGCTGGGCTATCGTTATGTT |
|  | Reverse | GAATCGGGTACGGAGCTTGG |
| S100A12 (human) | Forward | AGCATCTGGAGGGAATTGTCA |
|  | Reverse | GCAATGGCTACCAGGGATATGAA |
| HK3 (human) | Forward | GGACAGGAGCACCCTCATTTC |
|  | Reverse | CCTCCGAATGGCATCTCTCAG |
| CLEC4D (human) | Forward | CTGATACCTTCGGTTATTGCTGT |
|  | Reverse | GCACTCCTGTGCCTCTCTTAC |
| CLEC5A (human) | Forward | AGGTGGCGTTGGATCAACAA |
|  | Reverse | TTAGGCCAATGGTCGCACAG |
| ELF1 (human) | Forward | ATGGCTGCTGTTGTCCAACAGAAC |
|  | Reverse | CTAAAAAGAGTTGGGTTCCAGCAGTTC |
| 18s rRNA (human) | Forward | AGTCCCTGCCCTTTGTACACA |
|  | Reverse | CGATCCGAGGGCCTCACTA |
| ELF1 (mouse) | Forward | TGTCCAACAGAACGACCTAGT |
|  | Reverse | ACACAAGCTAGACCAGCATAAC |
| 18s rRNA (mouse) | Forward | AGTCCCTGCCCTTTGTACACA |
|  | Reverse | CGATCCGAGGGCCTCACTA |
