# Supplementary material for: Identification of hub genes and prediction of the ceRNA network in adult sepsis
Source: PeerJ. 2025 Aug 13;13:e19619. doi: 10.7717/peerj.19619 (PMC12357545; doi:10.7717/peerj.19619)
Supplement: Supplemental Information 14 [file peerj-13-19619-s014.docx]

| **S2 Table. Results of GSEA analysis of 2763 statistically changed genes** | | | | |
| --- | --- | --- | --- | --- |
| ID | NES | pvalue | qvalue | state |
| neutrophil degranulation | 3.104 | 1.00E-10 | 2.01E-08 | activated |
| innate immune system | 2.604 | 1.00E-10 | 2.01E-08 | activated |
| diseases of immune system | 2.3 | 5.65E-06 | 0.000453 | activated |
| toll like receptor cascades | 2.186 | 4.47E-05 | 0.002239 | activated |
| antigen processing cross presentation | 2.124 | 0.000219 | 0.008002 | activated |
| toll like receptor TLR1 TLR2 cascade | 2.093 | 0.000314 | 0.010508 | activated |
| interleukin 1 signaling | 1.994 | 0.001213 | 0.025597 | activated |
| rab geranylgeranylation | 1.964 | 0.000962 | 0.02422 | activated |
| interleukin 1 family signaling | 1.947 | 0.001484 | 0.028349 | activated |
| regulation of insulin like growth factor igf transport and uptake by insulin like growth factor binding proteins igfbps | 1.943 | 0.001136 | 0.025306 | activated |
| o linked glycosylation | 1.899 | 0.001093 | 0.025306 | activated |
| nucleotide binding domain leucine rich repeat containing receptor nlr signaling pathways | 1.868 | 0.001368 | 0.027425 | activated |
| processing of capped intron containing pre mRNA | -1.88 | 0.000864 | 0.02422 | inhibited |
| metabolism of RNA | -1.897 | 1.50E-05 | 0.001001 | inhibited |
| eukaryotic translation elongation | -1.962 | 0.001947 | 0.035494 | inhibited |
| mRNA splicing | -2.007 | 0.000633 | 0.019543 | inhibited |
| TCR signaling | -2.011 | 0.000966 | 0.02422 | inhibited |
| rRNA processing | -2.098 | 0.000121 | 0.004856 | inhibited |
| eukaryotic translation initiation | -2.331 | 8.57E-05 | 0.003821 | inhibited |
| PD 1 signaling | -2.482 | 4.01E-05 | 0.002239 | inhibited |
| Co-stimulation by the CD28 family | -2.734 | 2.02E-06 | 0.000203 | inhibited |
| generation of second messenger molecules | -2.959 | 3.88E-08 | 5.19E-06 | inhibited |
